# Urinary Collagen Peptides Predict Mortality

**DOI:** 10.1002/pmic.70131

**Published:** 2026-04-09

**Authors:** Maria Frantzi, Felix Keller, Agnieszka Latosinska, Joachim Beige, Alexandre Mebazaa, Anaïs Caillard, Dewei An, Paul Perco, Joost P. Schanstra, Lorenzo Catanese, Ralph Wendt, Harald Rupprecht, Jan A. Staessen, Antonia Vlahou, Harald Mischak, Justyna Siwy

**Affiliations:** ^1^ Department of Biomarker Research Mosaiques Diagnostics GmbH Hannover Germany; ^2^ Department of Internal Medicine IV (Nephrology and Hypertension) Medical University of Innsbruck Innsbruck Austria; ^3^ Department of Internal Medicine II Martin‐Luther‐University Halle‐Wittenberg Halle an der Saale Germany; ^4^ Renal Unit Kuratorium for Dialysis and Transplantation (KfH) Neu‐Isenburg Germany; ^5^ Department of Nephrology St. Georg Klinikum Leipzig Leipzig Germany; ^6^ Department of Anaesthesiology and Intensive Care Hospital Saint Louis‐Lariboisière Paris France; ^7^ Department of Cardiovascular Medicine Shanghai Institute of Hypertension Shanghai Key Laboratory of Hypertension National Research Centre For Translational Medicine State Key Laboratory of Medical Genomics Ruijin Hospital Shanghai Jiao Tong University School of Medicine Shanghai China; ^8^ Computational Biology Department Delta 4 GmbH, Vienna Austria; ^9^ Institut National de La Santé et De La Recherche Médicale (INSERM), UMR 1297 Institute of Metabolic and Cardiovascular Disease Toulouse France; ^10^ University of Toulouse Toulouse France; ^11^ Department of Nephrology Angiology and Rheumatology, Klinikum Bayreuth GmbH Bayreuth Germany; ^12^ Department of Nephrology Medizincampus Oberfranken Friedrich‐Alexander‐University Erlangen‐Nürnberg Erlangen Germany; ^13^ Kuratorium for Dialysis and Transplantation (KfH) Bayreuth Germany; ^14^ Non‐Profit Research Institute Alliance for the Promotion of Preventive Medicine Mechelen Belgium; ^15^ Biomedical Research Group Faculty of Medicine University of Leuven Leuven Belgium; ^16^ Center of Systems Biology Biomedical Research Foundation of the Academy of Athens Athens Greece

**Keywords:** collagen, extracellular matrix, fibrosis, mass spectrometry, mortality, urinary peptides

## Abstract

Organ fibrosis caused by the presence of excessive extracellular matrix (ECM) is related to mortality. Urinary peptide signatures were reported to be predictive of death in SARS‐CoV‐2 and chronic kidney disease. Such signatures were composed for 68% of collagen fragments. In this study, we examined whether urinary collagen peptides, potentially representing organ fibrosis, could predict mortality in patients with critical and non‐critical conditions. Urinary proteomic data from 1012 patients with follow‐up information from the CRIT‐COV‐U study were investigated for the association of collagen peptides with short‐term mortality. Independent datasets from 9193 patients were used for validation, including 1719 patients sampled at intensive care unit (ICU) admission and 7474 patients with other diseases (outside the ICU). A total of 607 collagen peptides were significantly associated with mortality. A classifier based on 210 collagen peptides (COL210) predicting mortality was developed and validated in patients in the ICU (ICU_HR_: 2.64; 95% CI:1.71‐4.10; *p*<0.001) and outside the ICU (Non‐ICU_HR_: 2.16 95% CI: 1.47–3.17; *p*<0.001), showing strong associations to mortality regardless underlying conditions. This study demonstrates a link between the presence of ECM fragments in urine, specifically collagens, and increased mortality risk. Such a non‐invasive collagen‐based predictor of mortality may serve as a basis for proteomics‐guided targeted intervention.

**Abbreviations**
BMI: body mass index
CKD: chronic kidney disease
ECM: extracellular matrix
eGFR: estimated glomerular filtration rate
HF: heart failure
ICU: intensive care unit
IFTA: interstitial fibrosis and tubular atrophy
IPF: idiopathic pulmonary fibrosis
IQR: interquartile range
MMP: matrix metalloproteinases
SGLT2: sodium glucose cotransporter 2
SVM: support vector machine

## 1 Introduction

Extracellular matrix (ECM) is the non‐cellular key component of the cellular microenvironment with various crucial functions [1]. As a highly dynamic structure, it continuously undergoes remodeling that, under physiological conditions, is a critical mechanism to regulate cell differentiation, establish and maintain stem cell niches, branch morphogenesis, but also support angiogenesis and wound repair, when necessary [2, 3]. Through these processes, ECM plays a fundamental role in preserving tissue architecture and function, but when improperly regulated, aberrant ECM remodeling can lead to fibrotic tissue formation.

**Significance of the Study**

Fibrosis, characterized by excessive extracellular matrix (ECM) deposition, is a central feature in chronic diseases and organ failure and is correlated with increased mortality. A literature search revealed a shift from static histological assessments of ECM deposition towards more dynamic approaches based on biomarkers of collagen turnover, which provide real‐time and potentially actionable insights into disease progression and predict adverse outcomes, including death. Key biomarkers include products of collagen synthesis, but also fragments generated through collagen degradation. These biomarkers have individually shown correlations with fibrosis severity, disease progression and survival. This study extends the growing evidence supporting the relevance of collagen turnover in mortality by focusing on collagen degradation markers across diverse fibrotic diseases, independent of the underlying pathology. It additionally explores the integration of collagen degradation fragments into risk model, potentially improving predictive accuracy and advancing precision medicine. Our study demonstrates that urinary collagen degradation markers integrated into a machine learning model (COL210) can identify “vulnerable” individuals across different fibrotic backgrounds, highlighting the potential utility for improved risk stratification and targeted interventions.

Fibrosis is characterized by the excessive deposition and remodeling of ECM, particularly collagens [4]. This process leads to progressive tissue stiffening and scarring, represents a hallmark of chronic diseases and is a critical driver of increased mortality across diverse pathological conditions, including idiopathic pulmonary fibrosis (IPF) [5, 6, 7, 8, 9, 10, 11], chronic kidney disease (CKD) [12, 13, 14], liver disease [15], heart failure (HF) [16, 17, 18, 19], and cancer [20, 21, 22]. In the context of organ fibrosis, the excessive ECM deposition impairs organ function by disrupting normal cellular architecture and interfering with cellular processes such as nutrient and oxygen diffusion [23]. Central drivers of fibrosis include fibroblast activation, myofibroblast differentiation, and an inflammatory response, often triggered by chronic injury or inflammation [24, 25]. These processes further promote a pro‐fibrotic environment, which amplifies fibrosis [25].

While increased ECM synthesis has traditionally been the primary focus of fibrosis research, it represents only one arm of ECM turnover. Degradation appears equally relevant, yet remains comparatively underexplored. A literature search conducted on 10/12/2024 using the MeSH terms “collagen” AND “fibrosis” AND “mortality” OR “death” OR “failure”, revealed a shift from static histological measures of ECM deposition to dynamic assessment, including analysis of specific biomarkers of collagen degradation, which may be linked to disease progression and predictive of adverse outcomes, such as mortality, especially in fibrotic diseases [6, 12, 15, 16, 19]. Dynamic biomarkers of collagen turnover can broadly be categorized into indicators of collagen synthesis, such as the pro‐peptides generated during collagen assembly [15] and degradation markers, collagen fragments from the mature collagen fiber, generated by for example matrix metalloproteinases (MMP) [26].

Previous reports from our group have revealed that peptide profiles in urine detected using capillary electrophoresis coupled to mass spectrometry (CE‐MS) are indicative of an individual's risk for disease progression and/or death [27]. This observation was initially linked to specific diseases, including CKD [28, 29], HF [16], SARS‐CoV‐2 infection [30], or cancer progression and metastasis [31]. Moreover, a classifier primarily generated to predict disease course after SARS‐CoV‐2 infection (COV50 classifier) was also predictive of mortality even in the absence of SARS‐CoV‐2 infection, indicating a pre‐existing vulnerability [32]. Similarly, urinary peptidomic profiles were predictive of mortality risk post‐acute SARS‐CoV‐2 infection [33]. These classifiers largely consist of naturally excreted urinary collagen fragments that result from collagen degradation, not from collagen synthesis. The observation that these degradation‐derived collagen peptides are associated with mortality across various conditions [32, 34] has led to the hypothesis that a solely collagen peptide‐based model may depict mortality risk that is not restricted to a specific underlying pathology or morbidity [35, 36, 37]. Based on this hypothesis, in this study we focus on urinary collagen peptides, representing a collagen degradation fingerprint, aiming to evaluate possible commonalities of chronic pathological conditions as depicted by altered collagen fragment excretion in urine. If specific collagen peptide profiles are associated with mortality, this fact could be exploited to guide intervention with existing therapeutic options or by introducing novel biologically driven targets to manipulate ECM turnover.

## 2 Materials and Methods

### 2.1 Study Population and Design

A total of four distinct cohorts were included in the study. The development cohort was used to identify collagen peptide biomarkers in urine associated with mortality and to develop a predictive classifier. Two additional, independent cohorts were employed to validate the classifier and assess its performance in predicting mortality across different datasets.

**FIGURE 1:**
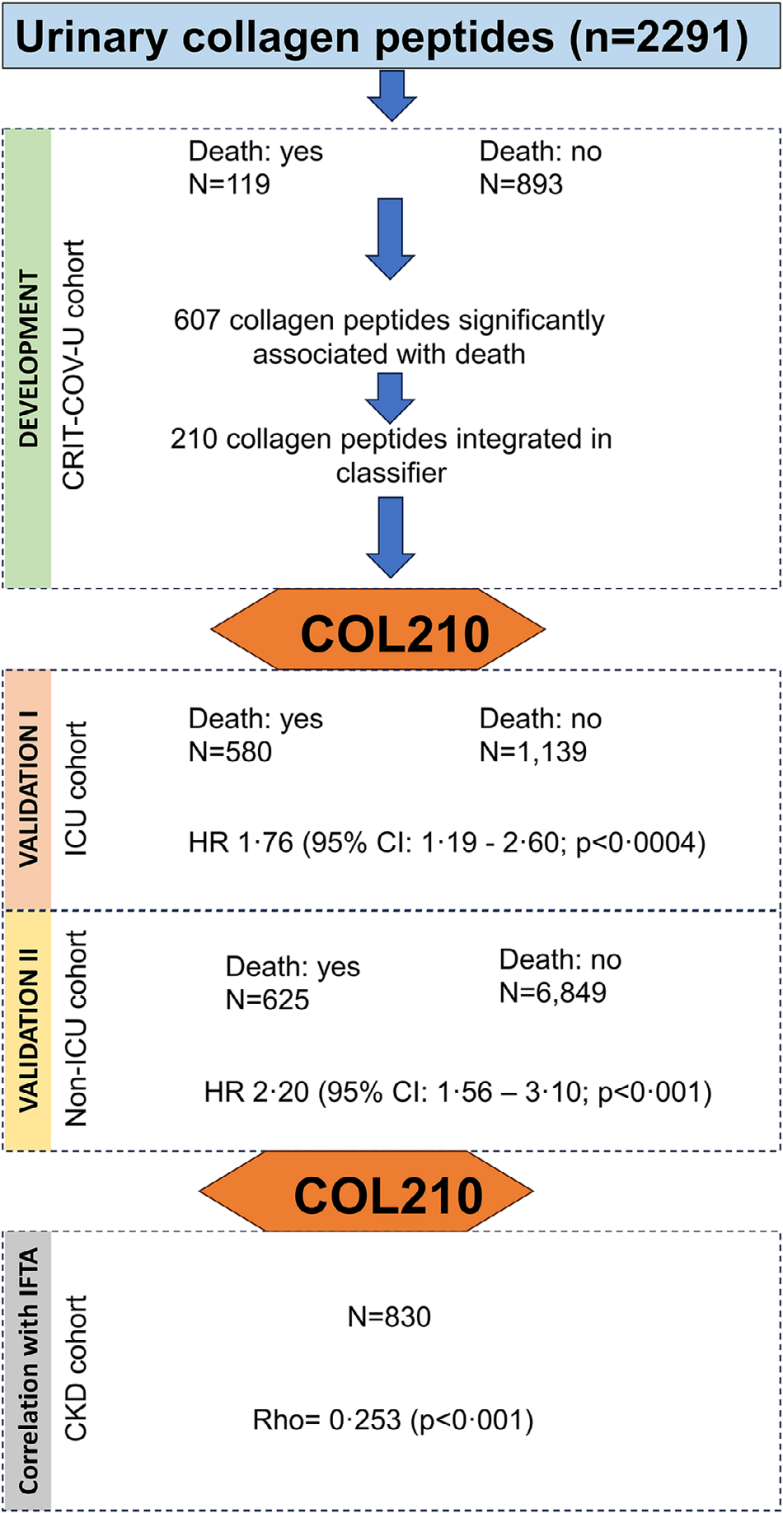
Study design.

Furthermore, based on the hypothesis that collagen peptides associated with mortality may also reflect underlying fibrosis, a fourth cohort of CKD patients with available data on fibrosis severity (interstitial fibrosis and tubular atrophy, IFTA) was analyzed to examine the correlation between classifier scores and the degree of fibrosis. The study design is illustrated in Figure 1.

**CRIT‐COV‐U cohort**: Data of the entire CRIT‐COV‐U cohort (*n* = 1012) were used as the development cohort [30]. Urinary collagen peptide abundance was analyzed in 893 patients who survived the acute infection and 119 patients who died within 28 days after infection. This cohort was also utilized for machine learning modeling and optimization of the COL210 predictive model.

**ICU cohort**: Data from 1719 patients from the medical, surgical, or mixed ICUs at 14 university hospitals from the FROG‐ICU study were considered [38]. These data were used as an independent validation cohort. Inclusion criteria were mechanical ventilation or administration of vasoactive agents for at least 24 h. Exclusion criteria were age under 18, severe head injury with a Glasgow Coma Scale below eight, brain death or persistent vegetative state, pregnancy or breastfeeding, transplantation in the past 12 months, moribund status, and lack of social security coverage. All CE‐MS datasets were complemented with 1‐year follow‐up and information on relevant co‐variables (age, body mass index (BMI), sex, blood pressure, estimated glomerular filtration rate (eGFR), presence of diabetes, kidney, cardiovascular disease, and hypertension). No pre‐selection criteria were applied. Patient characteristics are summarized in Table S1.

**Non‐ICU**: The second validation of the COL210 model was performed in a non‐ICU population using 7474 datasets extracted from the human urinary proteome database [39, 40]. These datasets were selected from previous studies including more than 50 individuals and with available follow‐up information by applying the same criteria as above regarding the availability of co‐variables. These datasets included patients with HF, diabetes, kidney diseases, or cancer, with patient characteristics provided in Table S2.

**CKD cohort**: To explore the association between urinary collagen peptides and kidney fibrosis, data from CKD patients with available histological assessments of fibrosis severity (IFTA) were used [41]. Table S3 includes the characteristics of this cohort.

#### 2.1.1 Urinary Mass Spectrometry Datasets

Urinary peptidomic profiles were acquired using CE–MS. The urinary proteome is well characterized and reference standards are available [42, 43]. Proteome analysis was performed on urine samples collected at study inclusion and bio‐banked until assayed. No preservatives were added. Detailed information on urine sample preparation, proteome analysis by CE‐MS, data processing, and sequencing of the urinary peptides allowing identification of parental proteins is available in previous publications [30, 43, 44, 45]. Briefly, samples were thawed after storage at −20°C or below. Storage duration varied across studies and may not have been specifically reported; however, previous biomarker studies using samples collected over approximately 10–15 years and stored frozen at −20°C or below until analysis have demonstrated the long‐term stability of urinary peptides during storage [46]. 700 µL of urine were mixed with 700 µL of 2 M urea and 10 mM NH_4_OH containing 0.02% SDS, followed by ultrafiltration using a Centristat centrifugal filter device with a 20 kDa molecular weight cut‐off (Sartorius, Göttingen, Germany). During this step, high‐molecular‐weight proteins were removed from the sample. The peptide‐containing fraction (<20 kDa) was subsequently passed through a PD‐10 gel filtration column (GE Healthcare Bio‐Sciences, Uppsala, Sweden) to remove urea, electrolytes, and salts, followed by lyophilization and storage at 4°C until measurement.

For CE–MS analysis, lyophilized samples were reconstituted in 10 µL of HPLC‐grade water and injected into the CE–MS system at 2 psi for 99s, corresponding to an injection volume of approximately 280nL. Analyses were performed using a P/ACE MDQ capillary electrophoresis system (Beckman Coulter, Fullerton, CA) coupled to a MicroTOF mass spectrometer (Bruker Daltonics, Bremen, Germany). The running buffer consisted of 20% acetonitrile in HPLC‐grade water supplemented with 0.94% formic acid. Electrospray ionization was applied using an interface from Agilent Technologies (Palo Alto, CA), operated at a potential of −4.0 to −4.5 kV. Mass spectra were acquired over an *m*/*z* range of 350–3000 and accumulated every 3s.

The acquired raw MS data were processed using the proprietary software MosaFinder software (version1.4). Data calibration was performed using 3151 internal standards as reference points for both mass and migration time, applying global linear regression for mass correction and local linear regression for migration time adjustment. To normalize the signal intensities, 29 collagen fragments, known to remain unaffected by the disease, were used as internal standards [47]. The study was performed for datasets that pass the established quality control criteria, including among others: at least 800 peptides had to be detected, with a minimum MS resolution of 8000, within a migration time window of at least 10 min. Following calibration, the average deviation in migration time relative to the reference standards was required to remain below 0.35 min [45].

Candidate biomarkers were sequenced using CE–MS/MS or liquid chromatography (LC)–MS/MS analysis, as described previously [30, 48]. Analyses were performed using either an Ultimate 3000 nano‐flow LC system (Dionex/LC Packings, USA) or a P/ACE MDQ CE system (Beckman Coulter, Fullerton, CA), both coupled to a Q Exactive Plus mass spectrometer (Thermo Fisher Scientific, Waltham, MA, USA). Full‐scan MS spectra (*m*/*z* 300–2000) were acquired in the Orbitrap, followed by data‐dependent selection of ions for fragmentation. Data files were searched against the UniProt human non‐redundant database using Proteome Discoverer version 2.4 with the SEQUEST search engine.

All datasets were from previously published studies and fully anonymized. These datasets were retrieved from the Human Urinary Proteome Database [40].

#### 2.1.2 Survival Endpoints

In the CRIT‐COV‐U cohort all participants were followed up until recovery, hospital discharge, or death. In this acute phase after infection, the vital status was recorded.

In the FROG‐ICU study, information on vital status was recorded 3, 6 and 12 months after ICU discharge, as previously described [49]. For the non‐ICU patients, vital status and outcome were assessed as described in the specific original studies [16, 28, 29, 36, 37, 44, 50, 51, 52, 53, 54, 55, 56, 57, 58, 59, 60].

#### 2.1.3 Statistics

In respect to descriptive statistics for the ICU and non‐ICU samples, as shown in Tables S1 and S2, median and first and third quartiles (IQR) were assessed for continuous variables, and absolute (N) and relative frequencies (%) for categorical variables. Hypotheses of no differences in scale or distribution of patient characteristics between the death and non‐death groups were tested with Wilcoxon–Mann–Whitney tests for continuous and with *χ* ^2^‐homogeneity tests for categorical variables. For the definition of the mortality‐associated peptides, peptides found in less than 10% of cases or controls were excluded. The collagen fragments abundance levels between the death and non‐death group of the CRIT‐COV‐U cohort were compared using the Wilcoxon–Mann–Whitney tests, as the peptidomics data were not normally distributed, followed by Benjamini–Hochberg‐based false discovery rate (FDR) correction.

Mortality per person‐time stratified by age and COL210 groups was estimated as the ratio of the number of the deceased to the sum of all patients’ observation times within each group, scaled to 100 person‐years. Unadjusted longitudinal risk of mortality was estimated as cumulative incidence using the Kaplan‐Meier estimator for non‐ICU and ICU patients. In the latter group, estimates were carried out over the whole observation time and separately within ICU and after ICU survival. Participants were categorized into four predefined age groups ((15–45], (45–60], (60–75], and (75–100]). Adjusted Cox regressions stratified by age groups were performed for non‐ICU and ICU patients separately. Models were adjusted for sex, presence of kidney disease, cardiovascular disease, hypertension, mean arterial pressure, and eGFR. To formally assess whether the association between COL210 and mortality differed by age group, interaction analyses were conducted using Cox models including a multiplicative interaction term between COL210 and age group. For a non‐ICU cohort, Cox regressions were also fitted within major underlying comorbidities. Cancer was not considered due to the low number of subjects and events in the cohort. To describe the distribution of COL210 between deceased and surviving subjects within each study, boxplots were used. The relationship between time until death and the COL210 score (as derived based on the machine learning classification) is displayed in a scatter plot, indicating ICU and non‐ICU groups by color and a local average smoother is applied to assess the relationship of COL210 on failure time. For statistical testing, a type 1 error of 5% was allowed, and all hypotheses tested were two‐sided. The hazard ratios (HR) were compared using *z*‐test. All analyses were carried out using R (version 4.2.2, R Core Team, Vienna, Austria) [61].

### 2.2 Generation of the COL210 Classifier

Abundance levels of urinary collagen peptides were modelled through a machine learning approach based on support vector machine (SVM) algorithms using MosaCluster proprietary software (version 1.6.5). The SVM model was trained in the CRIT‐COV‐U cohort. All peptides demonstrating a significant difference (p<0.05) between death and non‐death groups were initially included in the classifier as potential biomarkers. Feature selection was then performed using a leave‐one‐out (take‐one‐out) recursive procedure, in which the classifier was repeatedly re‐trained while systematically reducing the feature set. Features were removed step by step until the best performance was reached, stopping at the point where further reduction led to a decline in predictive accuracy.

The SVM binary classification model (any death event vs. survivors) was optimized (C and gamma) using leave‐one‐out cross‐validation. The optimal C and gamma parameters were: 10,200 and 0.000004, respectively with radial basis function kernel.

The COL210 classifier score was transformed into an estimate of the number of days to a 50% risk of death using a previously established approach [62]. The analysis was performed separately for ICU and non‐ICU cohort. The relationship between the COL210 score and the estimated time to a 50% risk of death was then assessed using regression analysis to establish the following predictive model: ICU: Log(Days to 50% risk of death, ICU cohort) = 2.6667–0.1238*COL210 score and Log(Days to 50% risk of death, non‐ICU cohort) = 3.7257‐0.1751*COL210 score (Figure S1).

### 2.3 Ethical Approval

All datasets were from previously published studies and fully anonymized. The Ethics Committee of the German‐Saxonian Board of Physicians (Dresden, Germany; number EKBR88/20.1) and the Institutional Review Boards of the recruiting sites provided ethical approval for the CRIT‐COV‐U. For the ICU cohort, the study was approved by our Institutional Review Board (IRB) (Comité de Protection des Personnes—Ile de France IV, IRB n°00003835 and Commission d’éthique biomédicale hospitalo‐facultaire de l'hôpital de Louvain, IRB n°B403201213352). For the CKD cohort the local ethics committee of the Friedrich‐Alexander Universität Erlangen‐Nürnberg provided approval for the nephrological biobank of the Klinikum Bayreuth (ethic approval code 264_20 B) and the urinary proteomics analysis (ethic approval code 221_20 B). Approval from the Ethics Committee of the Saxonian Board of Physicians, Dresden, Germany, was obtained for the study center Klinikum St. Georg Leipzig (ethic approval code EK‐BR‐14/20‐1). In addition, ethical review and approval are not required for this study due to all data being fully anonymized, based on the opinion of the ethics committee of the Hannover Medical School, Germany (no. 3116‐2016).

## 3 Results

### 3.1 Collagen Profiles in Urine are Predictive of Survival

Using CE‐MS peptidomics datasets from a prospective multicenter cohort of 1012 subjects (the CRIT‐COV‐U cohort) [30], we first investigated whether urinary collagen peptides detected in this cohort (*n* = 2291) are significantly different in abundance in urine from patients who survived the acute disease phase (*n* = 893) in comparison to those who died within 28 days (*n* = 119) after infection. This analysis resulted in the identification of 607 urinary collagen peptides significantly associated with death (FDR‐adjusted *p*<0.05, Table S4). Further investigation of correlations among peptide abundances, restricted to peptides detected in at least 50% of samples, demonstrated a high degree of internal consistency, supporting the overall quality and robustness of the dataset. Across 28,920 unique peptide pairs, the median Spearman's correlation coefficient was 0.25 (95% CI: −0.02 to 0.60). Of the 25,117 statistically significant pairs, 99% (24,929 pairs) exhibited positive correlations (Figure S2). At the level of single collagen peptides, of the peptides significantly associated with mortality, 208 (23.3%) were derived from COL1A1, 121 reduced, and 87 increased in abundance. Notably, the most significant COL1A1 peptides (top significant based on adjusted *p*‐value) were reduced in abundance and correspond to relatively large peptides (with molecular weight >3,000 Da). The second most frequent were fragments of COL3A1, with also high number of peptides reduced in abundance (51 of 96 mortality‐associated COL3A1 peptides). These were followed by fragments of COL1A2 (*n* = 23; 15 reduced in abundance) and COL2A1 (*n* = 31; 22 reduced in abundance). Most of the 607 collagen peptides originated from fibril‐forming collagens (*n* = 459), fibril‐associated collagens with interrupted triple helices (*n* = 49), and network‐forming collagens (*n* = 41), which were the three most highly represented groups (Figure S3A). We identified, for each class, the three proteins with the highest number of assigned peptides and mapped their locations along the corresponding collagen chains. In most cases, the urinary peptides originate from the central regions of mature collagen chains, indicating that they arise from collagen degradation pathways (Figure S4). Prediction of the proteases responsible for generating the collagen peptides associated with mortality indicated a predominant involvement of MMP, with MMP25, MMP13, and MMP9 being the three most prominent proteases based on the number of associated cleavage sites in both analyses (Table S5).

The 607 significant collagen peptides were combined into an SVM classifier. The classifier was trained and optimized using a take‐one‐out procedure in the 1012 CRIT‐COV‐U data. The classifier generated, termed COL210, included finally 210 different collagen fragments.

The peptides included in the model generally reflected the characteristics observed across the full set of 607 mortality‐associated peptides. Specifically, correlation analyses among the highly frequent model peptides were consistent with those of the complete peptide set (Figure S2B). Furthermore, the three most represented structural groups were fibril‐forming collagens (*n* = 171), fibril‐associated collagens with interrupted triple helices (*n* = 15), and network‐forming collagens (*n* = 10) (Figure S3B), and the top three proteases remained unchanged (Table S5).

### 3.2 COL210 Externally Validated in Predicting Survival in Patients Under Critical Condition

**FIGURE 2:**
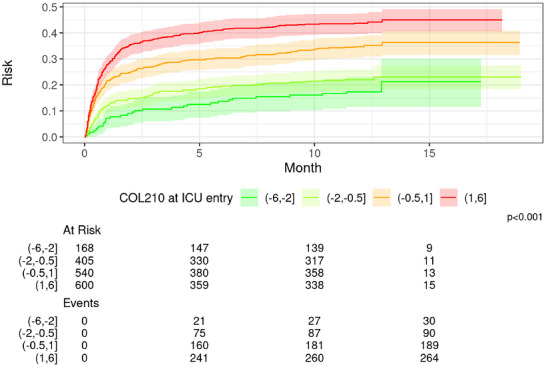
Survival curve depicting overall mortality in ICU group stratified into quartiles based on the COL210 score.

**FIGURE 3:**
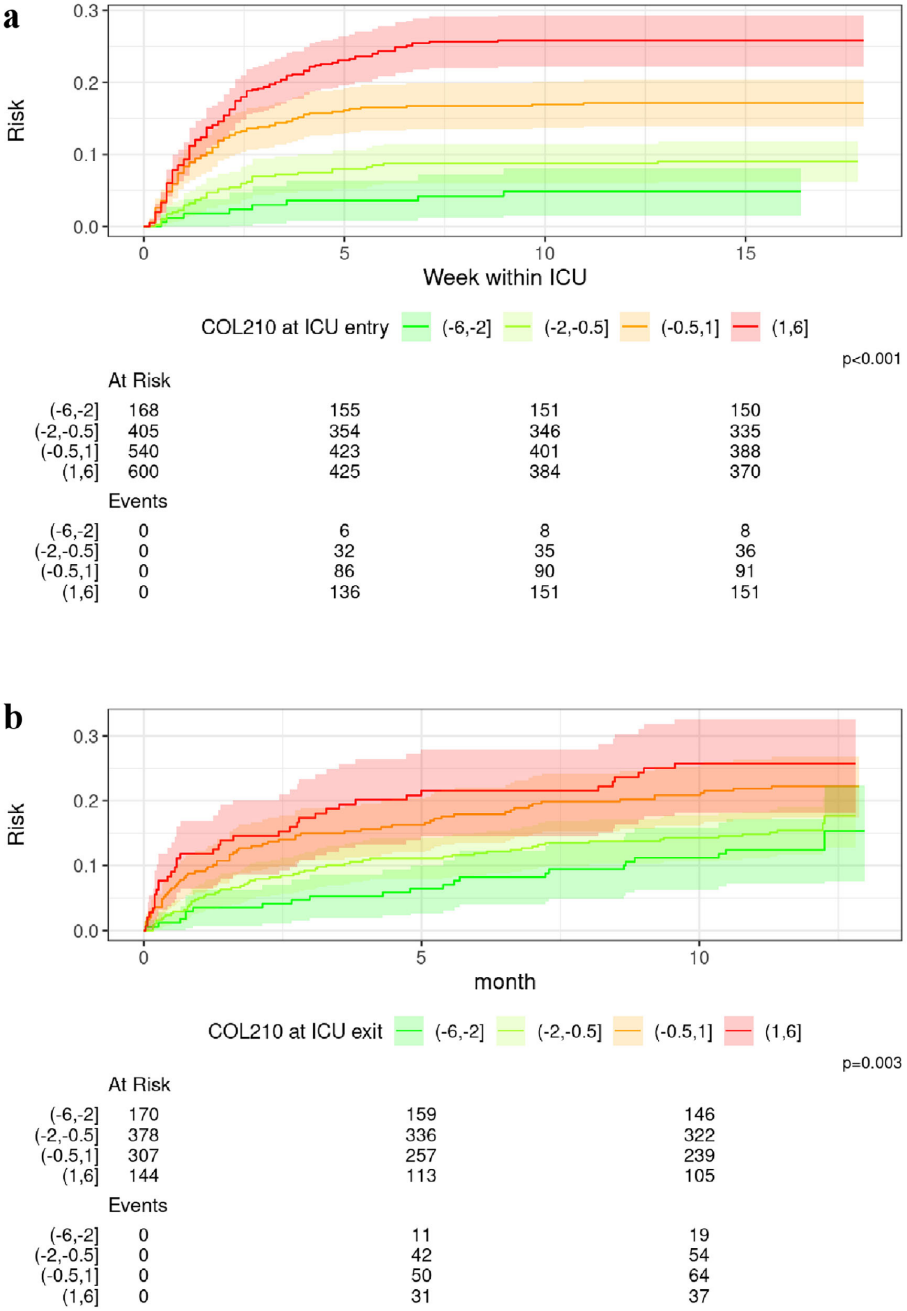
Survival curve depicting short‐term, in ICU (a) and long‐term, post‐ICU mortality (b) in patients with critical condition stratified into quartiles based on the COL210 score.

COL210 was first independently validated in patients in critical condition (ICU) (*n* = 1719 datasets, Table S1) [34]. Of the 1719 subjects, 580 (33.7%) died during a median follow‐up of 12 months. This included 287 deaths occurring in the ICU and 293 at a median of 47 days following hospitalization. Survival curves estimated using COL210 quartiles clearly showed that an increased COL210 score was associated with increased mortality (Figure 2). The highest COL210 score strata displayed an HR of 1.76 [95% confidence interval (CI): 1.19–2.6; *p*<0.001; Table S6]. Compared with the previously reported COV50 classifier [32], COL210 better predicted the mortality than COV50 (HR 1.2; 95% CI: 1.17–1.24; *p*<0.001). Outcomes for short term (deaths within ICU, HR 3.85; 95% CI: 1.87–7.93; *p*<0.001) but also long‐term mortality for patients that survived and exited ICU (HR 1.34, 95% CI: 0.76‐2.35; p = 0.3) are depicted (Figures 3A and 3B), with significant (*p* = 0.024) better prediction within the ICU. As no meaningful universal cutoff can be proposed for the COL210 classifier, the score was transformed into an estimate of the number of days to a 50% risk of death, with illustrative risk groups based on the COL210 score quintiles presented in Table S7.

### 3.3 COL210 predictive Value in Non‐Critical Patients (Non‐ICU)

**FIGURE 4:**
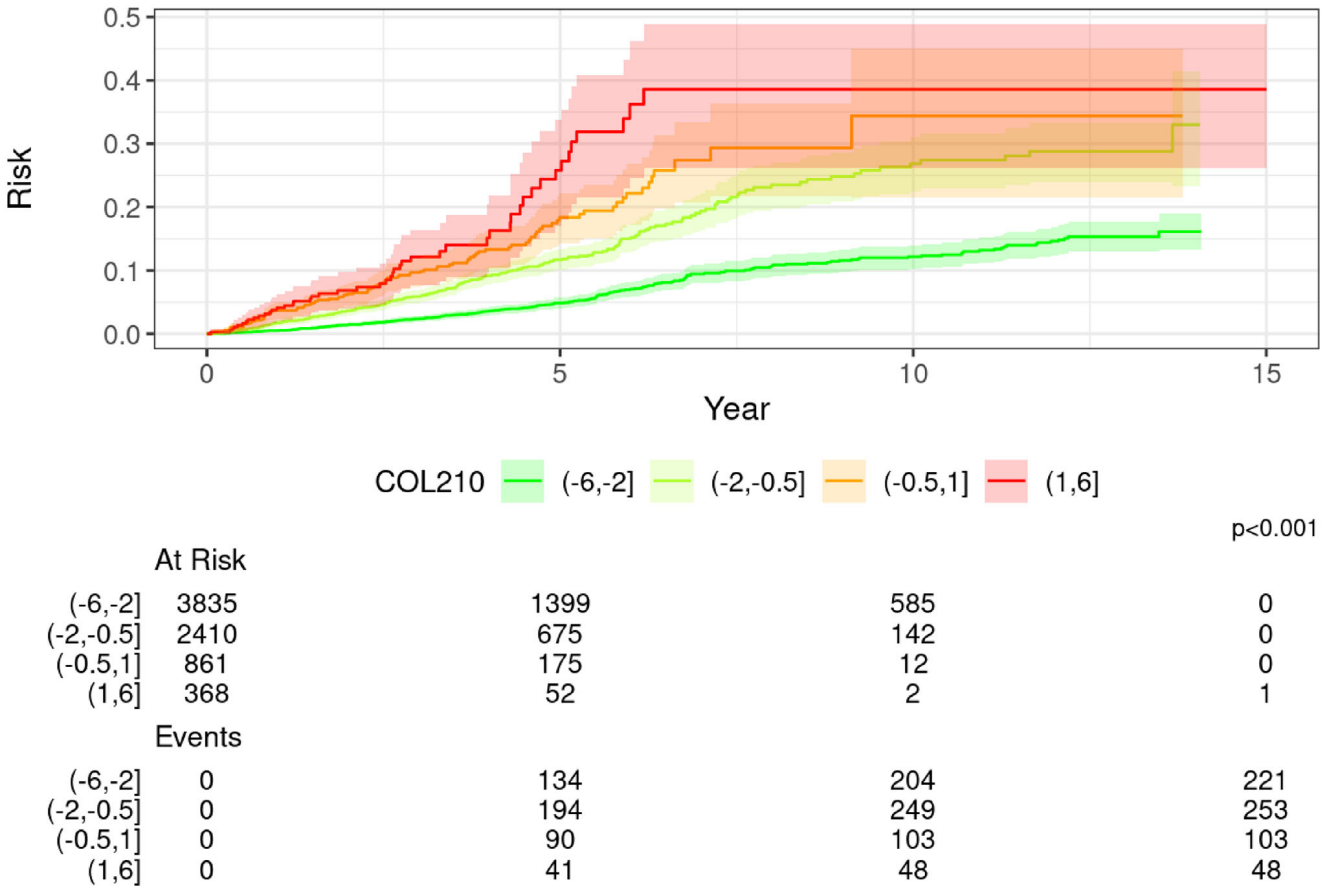
Survival curve depicting overall mortality in non‐ICU groups stratified into quartiles based on the COL210 score.

**FIGURE 5:**
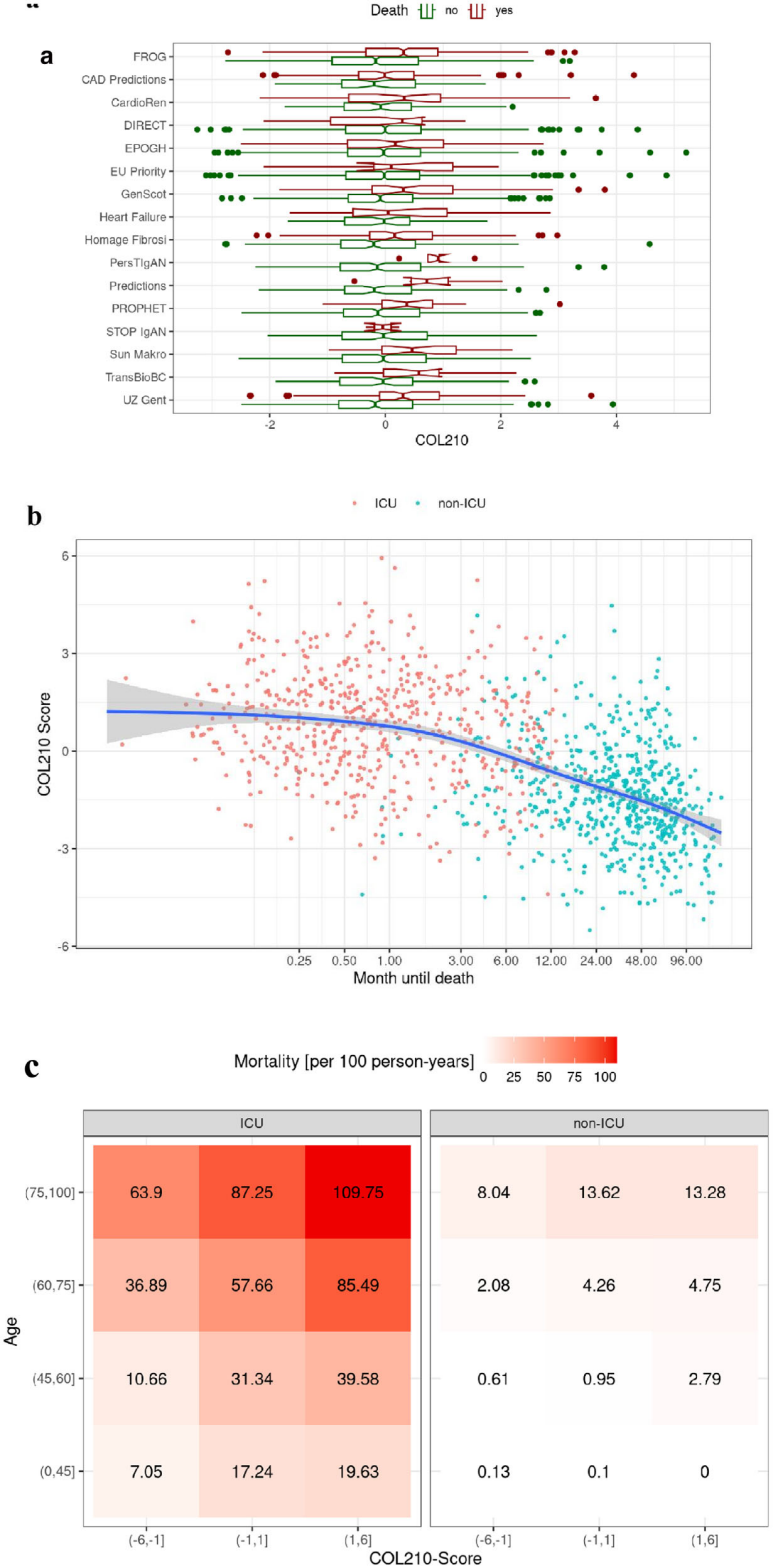
(a) Distribution plots of the COL210 score within the ICU (FROG) and the different study cohorts of the non‐ICU cohort. (b) COL210 score correlation with the time to death. (c) Mortality per person‐years for ICU and non‐ICU cohorts given age and COL210.

To further expand our analysis, we subsequently investigated if COL210 is predictive of future death in subjects without a critical condition. The numbers of subjects within the non‐ICU population per study split based on mortality events are summarized in Table S2. Of the 7474 subjects, 625 (8.4%) died during a median follow‐up of 47 months. Survival curves depicting overall mortality within the non‐ICU group stratified based on the COL210 score strata are shown in Figure 4, with adjusted HR estimated up to 2.20 for the highest COL210 score stratum (95% CI: 1.56–3.10; *p*<0.001; Table S8). Therefore, in both ICU and non‐ICU groups, higher COL210 scores are observed for patients that did not survive (Figure 5A), with a negative correlation of COL210 score with time to mortality (rho = ‐0.583, *p*<0.001 Figure 5B). As age is a crucial risk factor for death, we investigated the relationship between COL210 and mortality split by age groups (Figure 5C). Evidently, mortality is increased with both age and COL210. The risk groups based on COL210 score quintiles are presented in Table S9.

#### 3.3.1 Association of COL210 With Mortality Across Age Groups and Underlying Comorbidities

In age‐stratified Cox regression analyses among ICU patients, COL210 was significantly associated with mortality in patients aged 15–45 years (adjusted HR 1.23), 45–60 years (adjusted HR 1.23), and 60–75 years (adjusted HR 1.15). In contrast, no significant association was observed in patients aged 75–100 years (adjusted HR 1.04) (Table S10). Among non‐ICU patients, COL210 was not associated with mortality in the youngest age group (15–45 years; adjusted HR 1.12), but was significantly associated with increased mortality in patients aged 45–60 years (adjusted HR 1.24), 60–75 years (adjusted HR 1.21), and 75–100 years (adjusted HR 1.20) (Table S10).

When analyzing the ICU population, evidence of an interaction between age group and COL210 (*p* = 0.011) could be detected in the adjusted Cox analysis, indicating that the association between COL210 and mortality differed across age groups (Table S11). There was no evidence of interaction between age group and COL210 on mortality in non‐ICU cohort adjusted analyses (*p* = 0.76) (Table S11).

We also investigated the predictive performance of COL210 within individual disease groups and major underlying comorbidities, including cardiovascular disease, kidney disease, diabetes, and hypertension, to assess its cross‐disease applicability. Cancer was not considered due to the low number of subjects and events in the cohort. The predictive value of COL210 remained significant across the major comorbidities in the non‐ICU population (Table S12).

### 3.4 Association of COL210 With Kidney Fibrosis

To investigate the association between COL210 and kidney fibrosis, datasets from 830 patients with CKD of various aetiologies were analyzed. All patients had available data on the degree of fibrosis, assessed by IFTA. The COL210 classification scores were found to correlate significantly with IFTA, showing a Spearman correlation coefficient of rho = 0.253 (*p* < 0.0001), indicating statistically significant association between the COL210 classifier and the extent of kidney fibrosis. In parallel, Spearman correlations were analyzed within the most representative CKD aetiologies (Table S13). Although the effect size varies slightly across aetiologies, no statistically significant interaction was observed (Table S14).

## 4 Discussion

Recent organ‐centric studies and clinical trial data are shifting the research focus from a static perspective of tissue imaging for assessing excessive collagen deposition to a more dynamic investigation of peripheral (serum or urine‐based) collagen markers. These non‐invasive biomarkers can be indicative of fibrosis and further linked to organ failure and subsequent death. Importantly, this evolution is paralleled by a conceptual transition from investigating single diseases in isolation to a broader, integrative approach that identifies common molecular alterations across different organs and disease states. This allows for treatment strategies guided more by shared molecular signatures, such as collagen turnover, than by organ‐specific pathophysiology alone. Within kidney fibrosis, multiple urinary collagen‐derived peptides, especially from COL1A1 and COL3A1, were found to be significantly associated with CKD progression [63]. In subsequent studies, these findings could be confirmed and expanded, demonstrating a significant association of specific urine peptides with kidney fibrosis [41],. In liver fibrosis, composite biomarker panels quantifying collagen synthesis, like combinations of PRO‐C3, PRO‐C5, and degradation markers such as C6M, show promise for predicting fibrosis progression, regression, and survival outcomes [15], while in HF, markers of type I collagen turnover (e.g., urinary COL1A1 peptides) predict adverse cardiovascular events and mortality, demonstrating the clinical relevance of such noninvasive biomarkers for prognostication [16, 19].

In the context of the above literature and following previous evidence from our group reporting that collagen fragments naturally excreted in urine can be predictive of organ failure and death in organ‐specific studies, in this study we investigated whether we can generalize these findings. Specifically, we hypothesized that an exclusively collagen‐based model derived from CE‐MS collagen peptide reads in urine is predictive of mortality in a large and diverse population of patients at a critical condition or following chronic diseases involving a fibrotic environment. We were able to confirm the association of the collagen peptide‐based classifier COL210 with the degree of kidney fibrosis in a large cohort of CKD patients. In an age‐stratified analysis, the absence of an association in younger non‐ICU patients likely reflects the low number of deaths in this group, whereas the attenuation of the association in the oldest ICU patients may be due to high baseline mortality and competing risks that limit the relative impact of COL210. Age modified the association between COL210 and mortality in ICU patients but not in non‐ICU patients, suggesting that the prognostic relevance of COL210 varies with both age and illness severity.

The data presented are in agreement with multiple previous observations from CE‐MS‐based analysis of urinary peptides. In the context of HF, He and colleagues [16] reported significant reduction of the abundance of COL1A1 peptides from the central part of the collagen molecule associated with HF mortality, while several N‐ and C‐terminal peptides were found increased. This is in line to our results supporting that COL1A1 central and relatively large peptides also associate with mortality. CKD273, a classifier based on 273 peptides significantly associated with CKD, which contains as a major component reduced abundance of several COL1A1 peptides, was also found associated with increased risk of mortality in several studies [27, 64]. In addition, also in the COV‐50 classifier, reduction of the abundance of specific COL1A1 peptides was found associated with mortality, both in patients with SARS‐COV‐2, but also in patients not infected with SARS‐COV‐2, further supporting the findings of this study. The COL210 peptide classifier improved the predictive value in comparison with previous peptide panels developed for the detection of patients at critical state after SARS‐CoV‐2 infection [30, 32]. Of the 34 collagen‐derived peptides contained in the COV‐50 classifier, 20 overlap with the 210 collagen peptides identified here, all with identical trend in regulation. Six of these 20 also overlap with CKD273, further indicating commonality in the underlying biology.

The observation of reduced abundance of larger collagen I fragments associated with mortality is in line with a very recent publication (and data interpretation) from Mina et al., [65]. The authors aimed to find an explanation for the simultaneous increase and decrease of certain collagen fragments with CKD, a condition tightly associated with kidney fibrosis. Investigating the distribution of these peptides in detail, the authors presented a model suggesting initial degradation of collagen by endoproteases, likely members of the MMP family, resulting in the generation of larger peptides, which is attenuated in CKD. The same model is also applicable on the observations of this study, which suggests attenuation of collagen degradation, likely resulting in increased fibrosis, as associated with, and possibly a significant contributor to increased risk of mortality. This study extends previous findings by demonstrating, for the first time, that a collagen‐based urinary peptide signature (COL210) predicts mortality in patients with different conditions. Since the study evaluated also long‐term mortality in ICU and non‐ICU cohorts, rather than acute outcomes driven by acute systemic inflammation, cytokine storm, or endothelial injury, it is expected that the COL210 signature reflects rather a chronic, endogenous vulnerability, a physiological state that predisposes individuals to adverse outcomes regardless of the immediate clinical trigger. This observation highlights the potential of collagen‐derived urinary biomarkers as a generalizable indicator of systemic fibrotic burden and adverse outcomes across diseases. This interpretation is consistent with findings from previous work on COV50 in the general population [32], where similar peptide pattern was associated with long‐term risk independent of acute illness. Importantly, the use of a non‐invasive urine test provides a practical advantage for monitoring high‐risk patients, also the ones recovering from SARS‐CoV‐2, where early identification of those at increased risk of death could enable closer follow‐up and timely interventions.

Our data support the concept that intervention addressing fibrosis, for example via therapeutic drugs or lifestyle adjustments, may result as an additional benefit in the reduction of mortality. These thoughts are in general in agreement with the different effects observed in the context of sodium glucose cotransporter 2 (SGLT2) inhibition. This class of compounds, initially developed as antidiabetic drugs, has, in addition, demonstrated significant impact on mortality, and also on fibrosis. In a recent report [66], we could also demonstrate significant increase of several urinary collagen fragments in patients after SGLT2 inhibitor treatment, indicating that these drugs, possibly via inducing a metabolic switch, may result in reduction of highly reactive (oxygen) radicals, reducing collagen modification and crosslinking, and in this way enabling more efficient physiological collagen degradation, ideally even resolution of fibrosis. This hypothesis, although it has to be proven in additional experiments, would explain the above‐mentioned observations.

The study presents with limitations, especially due to its retrospective design based on previously collected data. We also cannot exclude that other disease‐specific mechanisms may also contribute to mortality risk. Nevertheless, the large number of datasets, the high number of endpoints assessed, and the high degree of homogeneity of the obtained findings strongly support that the results can be generalized. Along these lines, a strength of this analysis is the inclusion of datasets from multiple studies and across different conditions, indicating a robust basis for the assessment.

Collectively, in this study we demonstrate that multiple specific urinary collagen peptides are significantly associated with future death in both patients at critical condition and those without. These peptides can be combined into a classifier, COL210, that enables the detection of “vulnerable” subjects, irrespective of the underlying conditions. The COL210 score was also as correlated with the degree of fibrosis. This supports the rising clinical utility of collagen turnover biomarkers in predicting mortality across various fibrotic conditions and highlights the importance of dynamic markers assessing ECM. By integrating these biomarkers into risk models and leveraging advanced analytical methods like machine learning, predictive accuracy is enhanced, offering potential for improved prognostication and targeted interventions. The results indicate that personalized intervention guided by urinary collagen fragments may significantly improve outcomes and extend lifespan. Demonstrating the validity and extend of this assumption is the aim of a planned prospective study.

## 5 Associated Data

The data are not publicly available due to privacy or ethical restrictions. Anonymized data can be made available to investigators after sending a proposal to the corresponding author. Proposals will be reviewed and approved by the investigators, and collaborators on the basis of scientific merit and submitted for approval to the relevant ethics committee.

## Conflicts of Interest

HM is the cofounder and co‐owner of Mosaiques Diagnostics (Hannover, Germany) and AL, MF, and JS are employees of Mosaiques Diagnostics. PP is employee of Delta4 GmbH. AM reports grants or contracts from 4TEEN4, Abbott, Roche and Sphyngotec, and consulting fees from Roche, Adrenomed, Corteria, Fire1 and payment or honoraria from Merc and Novartis. All other authors declare no competing interests.

## Supporting information




**Supporting File 1**: pmic70131‐sup‐0001‐SuppMat.pdf.

**Supporting File 2**: pmic70131‐sup‐0002‐Tables.pdf.

## Data Availability

The data that support the findings of this study are available on request from the corresponding author. The data are not publicly available due to privacy or ethical restrictions.
