# Supplementary material for: Urinary Collagen Peptides Predict Mortality
Source: Proteomics. 2026 Apr 9;26(7):98–110. doi: 10.1002/pmic.70131 (PMC13327709; doi:10.1002/pmic.70131)
Supplement: Supplementary file 1 — Supporting File 1: pmic70131‐sup‐0001‐SuppMat.pdf. [file PMIC-26--s002.pdf]

Supplementary Figures

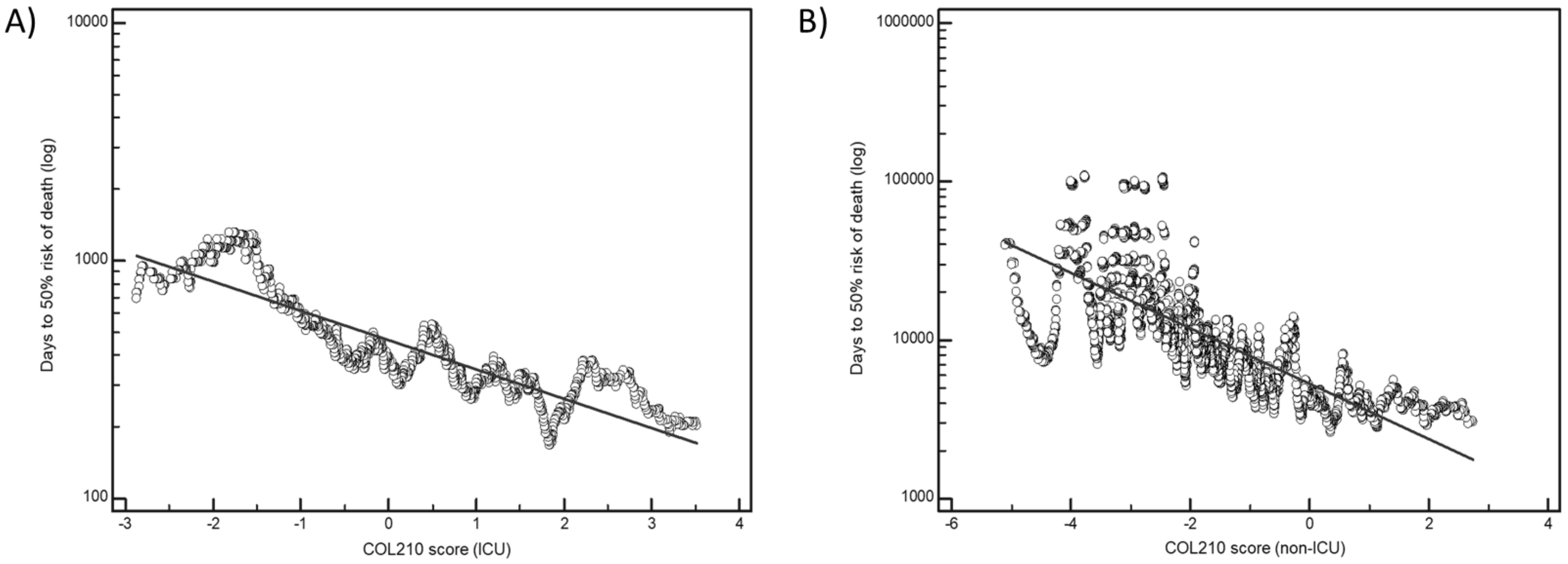

Supplementary Figure 1. Relationship between the COL210 score and the estimated time to a 50% event rate in the ICU (A) and non-ICU (B) cohorts.

A)

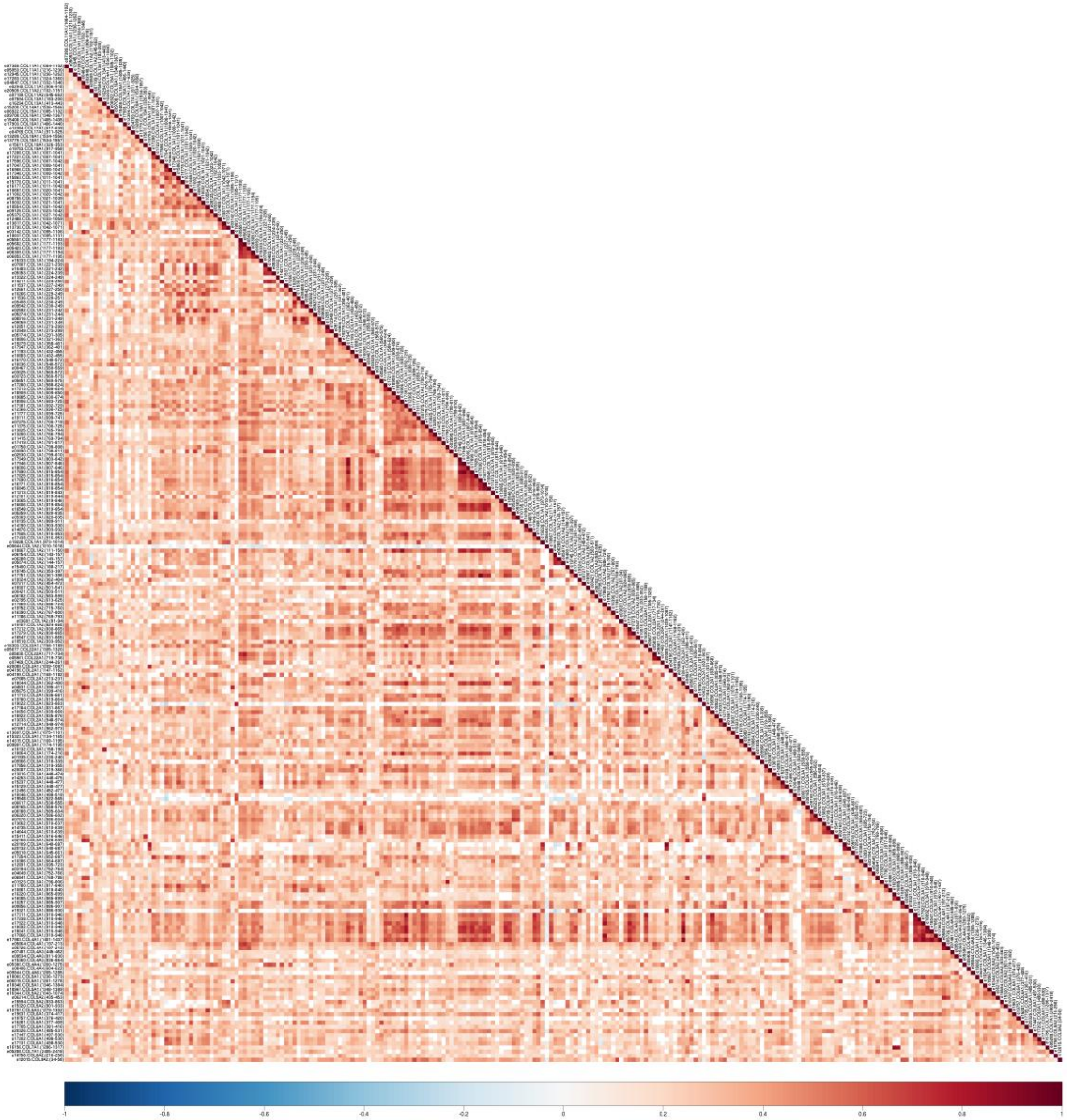

B)

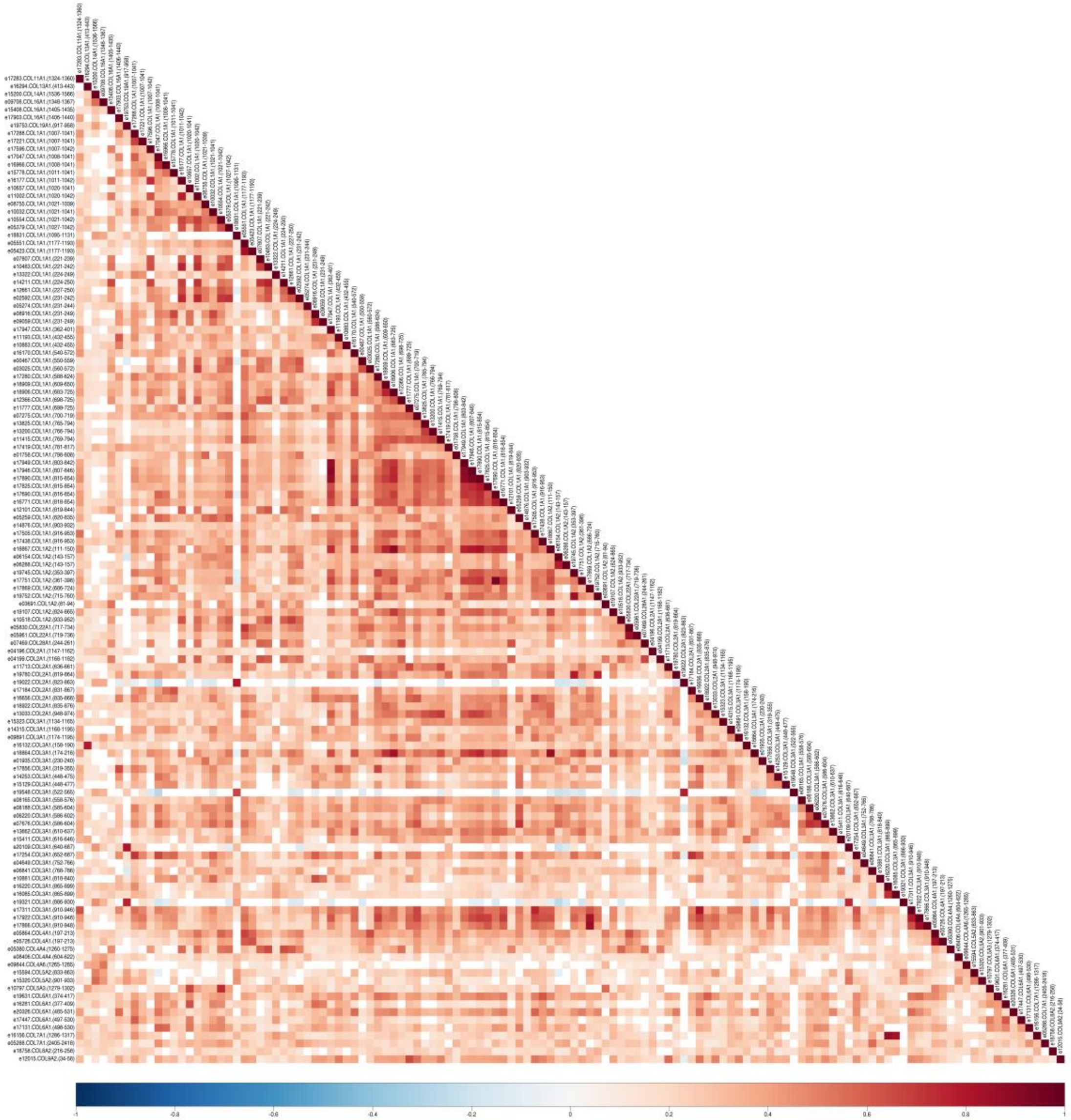

Supplementary Figure 2. Heatmaps presenting Spearman's rho values for correlations among the most frequent 607 (A) and 210 (B) mortality-associated peptides in the discovery dataset. Only peptide pairs in which both peptides were detected in at least 50% of samples were included.

A)

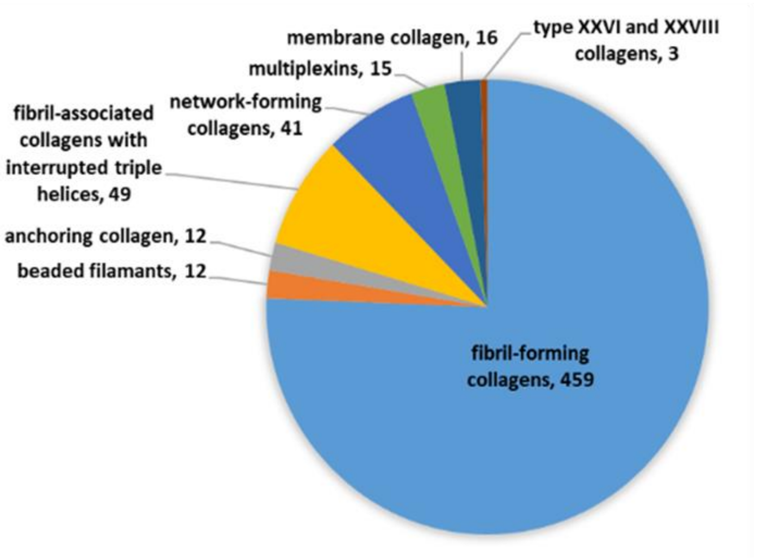

B)

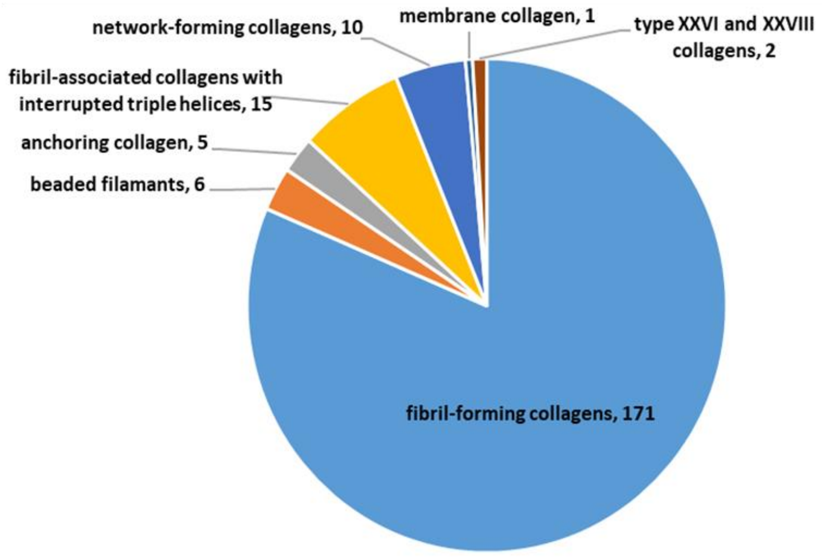

Supplementary Figure 3. Pie charts illustrating collagen structural classes based on the distribution of the 607 peptides associated with mortality (A) and the 210 peptides included in the COL210 classifier (B).

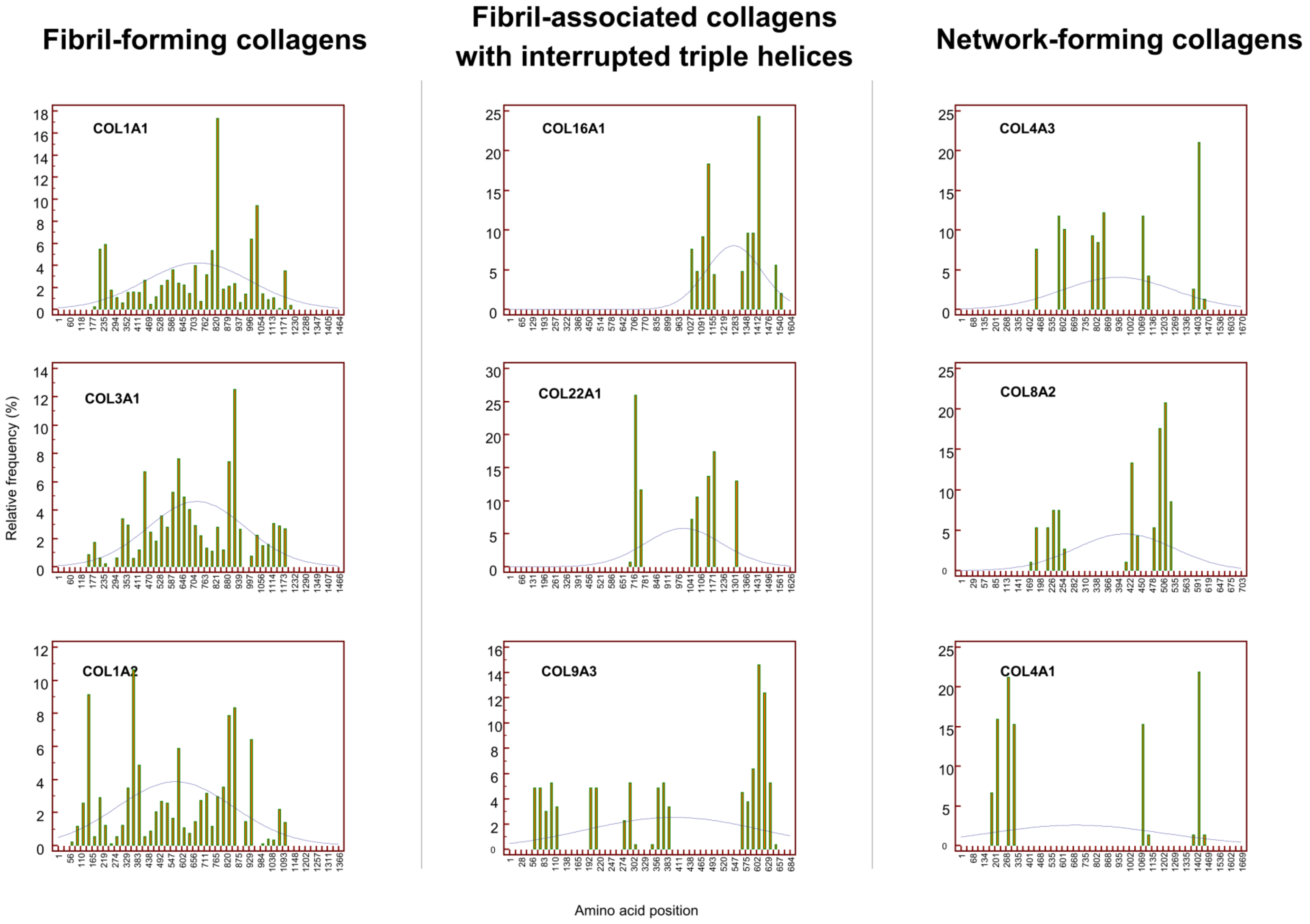
Fibril-forming collagens
Fibril-associated collagens with interrupted triple helices
Network-forming collagens
COL1A1
COL16A1
COL4A3
COL3A1
COL22A1
COL8A2
COL1A2
COL9A3
COL4A1
Relative frequency (%)
Amino acid position

Supplementary Figure 4. Histograms showing amino acid distributions of mortality-associated collagen peptides for selected parental proteins. The three proteins with the highest number of assigned peptides per collagen structural group are shown, with peptide locations mapped along the corresponding collagen chains. To increase coverage, the analysis was performed using all 607 mortality-associated peptides.
