# Supplementary material for: Urinary Collagen Peptides Predict Mortality
Source: Proteomics. 2026 Apr 9;26(7):98–110. doi: 10.1002/pmic.70131 (PMC13327709; doi:10.1002/pmic.70131)
Supplement: Supplementary file 2 — Supporting File 2: pmic70131‐sup‐0002‐Tables.pdf. [file PMIC-26--s001.pdf]

Supplementary Tables

Table of Contents

Supplementary Table 1: Descriptive statistics for the ICU patients considered within this study. 1

Supplementary Table 2: Descriptive statistics for the Non-ICU patients considered within this study. 2

Supplementary Table 3: Descriptive statistics for the CKD patients considered within this study. 4

Supplementary Table 4: List of 607 significant collagen peptides. 5

Supplementary Table 5: List of proteases responsible for generating the collagen peptides associated with mortality. 23

Supplementary Table 6: Prediction of short-term mortality in patients with critical condition stratified based on the COL210 score range. 24

Supplementary Table 7: Risk groups based on COL210 score quintiles in the ICU cohort. Days to 50% risk of death were estimated based on the established regression analysis. 26

Supplementary Table 8: Prediction of mortality in patients with non-critical condition. 27

Supplementary Table 9: Risk groups based on COL210 score quintiles in the non-ICU cohort. Days to 50% risk of death were estimated based on the established regression analysis. 29

Supplementary Table 10: Age-stratified Cox regression analyses among ICU and non-ICU patients. 30

Supplementary Table 11: Association between COL210 and mortality in ICU and non-ICU patients. 31

Supplementary Table 12: Underlying comorbidity–stratified Cox regression analyses among non-ICU patients. Hazard ratios (HRs) and 95% confidence intervals (CIs) are reported. 32

Supplementary Table 13: Correlation of the COL210 score with IFTA stratified by CKD aetiology. 33

Supplementary Table 14: Fisher’s r-to-z comparison of independent correlations between selected CKD aetiologies. 34

Supplementary Table 1: Descriptive statistics for the ICU patients considered within this study.

	Level/Unit	Overall	Death: no	Death: yes	p
N		1719 (100%)	1139 (66.3%)	580 (33.7%)	
Study	FROG	1719 (100%)	1139 (100%)	580 (100%)	
Age	[yrs]	62 (50, 73)	58 (46, 69)	70 (61, 78)	<0.001
Female	yes	602 (35%)	414 (36%)	188 (32%)	0.11
BMI	[kg/m²]	26.2 (22.9, 30.0)	26.2 (22.8, 29.9)	26.4 (23.1, 30.1)	0.6
Systolic BP	[mmHg]	123 (109, 140)	124 (110, 140)	120 (107, 139)	0.01
Diastolic BP	[mmHg]	64 (55, 75)	66 (56, 77)	60 (52, 70)	<0.001
Mean Arterial BP	[mmHg]	84 (74, 95)	86 (76, 96)	80 (71, 92)	<0.001
Hypertension	yes	979 (57%)	602 (53%)	377 (65%)	<0.001
eGFR	[ml/min/1.73 m²]	87 (48, 127)	97 (57, 132)	67 (37, 107)	<0.001
Kidney Disease	yes	716 (42%)	378 (33%)	338 (58%)	<0.001
Diabetes	yes	280 (16%)	160 (14%)	120 (21%)	<0.001
Cardiovascular Disease	yes	98 (5.7%)	49 (4.3%)	49 (8.4%)	<0.001
COV50		1.17 (0.34, 1.83)	1.01 (0.11, 1.74)	1.45 (0.72, 1.97)	<0.001
FU Duration	[month]	12.0 (2.0, 12.1)	12.0 (12.0, 12.5)	0.7 (0.3, 2.1)	<0.001

Categorical variables are described with absolute (N) and group-wise relative frequencies (%), continuous variables with median (IQR). P-values for group differences result from chi-squared homogeneity tests for categorical and for Wilcoxon rank sum test for continuous variables. Abbreviations: BMI= body mass index; BP= blood pressure; eGFR= estimated glomerular filtration rate; FU= follow-up; ICU= intensive care unit; yrs= years

Supplementary Table 2: Descriptive statistics for the non-ICU patients considered within this study.

	Level/Unit	Overall	Death: no	Death: yes	p
N		7474 (100%)	6849 (91.6%)	625 (8.4%)	
Study					<0.001
	CAD Predictions	145 (1.9%)	50 (0.7%)	95 (15%)	
	CardioRen	116 (1.6%)	87 (1.3%)	29 (4.6%)	
	DIRECT	1487 (20%)	1448 (21%)	39 (6.2%)	
	EPOGH	914 (12%)	850 (12%)	64 (10%)	
	EU Priority	1,769 (24%)	1,756 (26%)	13 (2.1%)	
	GenScot	473 (6.3%)	417 (6.1%)	56 (9.0%)	
	Heart Failure	84 (1.1%)	67 (1.0%)	17 (2.7%)	
	Homage Fibrosis	354 (4.7%)	229 (3.3%)	125 (20%)	
	PersTIgAN	270 (3.6%)	265 (3.9%)	5 (0.8%)	
	Predictions	91 (1.2%)	85 (1.2%)	6 (1.0%)	
	PROPHET	462 (6.2%)	444 (6.5%)	18 (2.9%)	
	STOP IgAN	109 (1.5%)	107 (1.6%)	2 (0.3%)	
	Sun Makro	581 (7.8%)	556 (8.1%)	25 (4.0%)	
	TransBioBC	131 (1.8%)	117 (1.7%)	14 (2.2%)	
	UZ Gent	488 (6.5%)	371 (5.4%)	117 (19%)	
Age	[yrs]	60 (48, 68)	59 (47, 66)	73 (66, 79)	<0.001
Female	yes	2857 (38%)	2657 (39%)	200 (32%)	<0.001
BMI	[kg/m²]	27.5 (24.3, 31.2)	27.6 (24.3, 31.4)	26.9 (23.7, 30.1)	<0.001

	Level/Unit	Overall	Death: no	Death: yes	p
Systolic BP	[mmHg]	132 (121, 145)	132 (121, 144)	138 (124, 153)	<0.001
Diastolic BP	[mmHg]	79 (72, 85)	79 (73, 85)	75 (67, 82)	<0.001
Mean Arterial BP	[mmHg]	97 (90, 104)	97 (90, 104)	97 (88, 105)	0.3
Hypertension	yes	3090 (41%)	2758 (40%)	332 (53%)	<0.001
eGFR	[ml/min/1.73 m²]	82 (59, 99)	84 (62, 100)	61 (37, 80)	<0.001
Kidney Disease	yes	2212 (30%)	1898 (28%)	314 (50%)	<0.001
Diabetes	yes	4101 (55%)	3938 (57%)	163 (26%)	<0.001
Cardiovascular Disease	yes	1357 (18%)	983 (14%)	374 (60%)	<0.001
COV50		-1.88 (-2.33, -1.27)	-1.89 (-2.34, -1.30)	-1.69 (-2.26, -0.94)	<0.001
FU Duration	[month]	47 (29, 67)	48 (29, 67)	38 (19, 62)	<0.001

Categorical variables are described with absolute (N) and group-wise relative frequencies (%), continuous variables with median (IQR). P-values for group differences result from chi-squared homogeneity tests for categorical and for Wilcoxon rank sum test for continuous variables. Abbreviations: BMI= body mass index; BP= blood pressure; eGFR= estimated glomerular filtration rate; FU= follow-up; yrs= years

Supplementary Table 3: Descriptive statistics for the CKD patients considered within this study.

	**Level/Unit**	**Overall**
N		830 (100%)
Age	[yrs]	60 (47, 72)
Female	yes	311 (37%)
BMI	[kg/m²]	27.9 (24.3, 32.1)
Systolic BP	[mmHg]	134 (120, 149)
Diastolic BP	[mmHg]	79 (70, 84)
eGFR	[ml/min/1.73 m²]	29 (14, 52)
IFTA	[%]	20 (7.5, 35)

Categorical variables are described with absolute (N) and group-wise relative frequencies (%), continuous variables with median (IQR). Abbreviations: BMI= body mass index; BP= blood pressure; eGFR= estimated glomerular filtration rate; yrs= years, IFTA=interstitial fibrosis and tubular atrophy

Supplementary Table 4: List of 607 significant collagen peptides.

Pep-tide ID	Mass [Da]	CE-time [Min]	Sequence	Symbol	Protein name	p-value	Adj. p-value (BH)	Average cases	Average controls	Fold change	Freq. cases	Freq. controls
e09627	1962.81	21.88	PpGPpGKNGDDGEAGKpGRp	COL1A1	Collagen alpha-1(I) chain	6.35E-08	1.58E-06	4.66	64.70	0.07	5.88	28.67
e16641	3117.45	31.20	GADGQPGAKGEpGDAGAKGDAGPPGPAGPAGPPGPIG	COL1A1	Collagen alpha-1(I) chain	2.57E-03	1.26E-02	2.83	17.58	0.16	3.36	12.77
e15230	2824.29	29.28	AGpPGApGApGApGPVGPAGKSGDRGETGPAGP	COL1A1	Collagen alpha-1(I) chain	1.44E-04	1.15E-03	22.25	130.71	0.17	8.40	23.07
e13850	2587.19	21.13	PpGKNGDDGEAGKPGRpGERGppGPQ	COL1A1	Collagen alpha-1(I) chain	4.47E-09	1.74E-07	24.47	134.77	0.18	11.76	39.08
e16966	3193.43	22.57	PpGESGREGApGAEGSpGRDGSPGAKGDRGETGP	COL1A1	Collagen alpha-1(I) chain	4.95E-17	1.13E-13	114.97	578.06	0.20	34.45	72.12
e01399	1050.48	26.93	DGRpGPpGPpG	COL1A1	Collagen alpha-1(I) chain	4.38E-09	1.73E-07	55.58	247.84	0.22	25.21	51.40
e18705	3660.71	33.06	GADGQPGAKGEpGDAGAKGDAGPPGpAGPAGpPG-PIGNVGApG	COL1A1	Collagen alpha-1(I) chain	6.01E-05	5.48E-04	6.33	27.08	0.23	6.72	22.40
e17162	3238.50	30.87	GPAGFAGpPGADGQPGAKGEPGDAGAKGDAGPpGPAGP	COL1A1	Collagen alpha-1(I) chain	3.66E-03	1.70E-02	6.22	26.17	0.24	7.56	17.58
e05294	1474.65	20.07	GRDGSpGAKGDRGET	COL1A1	Collagen alpha-1(I) chain	1.08E-05	1.19E-04	10.98	38.79	0.28	21.85	41.77
e17768	3381.56	31.78	GAPGAEGSPGRDGSPGAKGDRGETGPAGPPGApGApGAp	COL1A1	Collagen alpha-1(I) chain	7.12E-05	6.39E-04	12.79	41.05	0.31	8.40	24.75
e17690	3359.58	31.68	pPGADGQPGAKGEpGDAGAKGDAGppGPAGPAGPPGPIG	COL1A1	Collagen alpha-1(I) chain	1.52E-15	1.16E-12	204.20	655.21	0.31	52.94	81.86
e19431	3946.86	21.87	SEGPQGVRGEPGppGpAGAAGPAGNPGADGQPGAKGAn-GAPGIAGA	COL1A1	Collagen alpha-1(I) chain	8.33E-11	6.00E-09	87.67	265.38	0.33	21.01	53.75
e02592	1186.53	22.31	DDGEAGKpGRpG	COL1A1	Collagen alpha-1(I) chain	1.94E-07	3.80E-06	71.82	213.21	0.34	34.45	56.10
e00220	860.36	25.66	DDGEAGKpG	COL1A1	Collagen alpha-1(I) chain	5.93E-07	9.37E-06	63.16	182.97	0.35	12.61	35.61
e16708	3133.47	31.24	GADGQPGAKGEpGDAGAKGDAGPpGPAGPAGPPGPIG	COL1A1	Collagen alpha-1(I) chain	1.54E-06	2.08E-05	34.33	96.86	0.35	13.45	35.16
e17825	3400.59	32.15	GPpGADGQPGAKGEPGDAGAKGDAGPPGpAGPAGPpGPIG	COL1A1	Collagen alpha-1(I) chain	3.12E-09	1.26E-07	107.53	299.71	0.36	36.13	61.48
e18006	3448.59	32.10	PpGpAGFAGpPGADGQPGAKGEPGDAGAKGDAGPPGPAGP	COL1A1	Collagen alpha-1(I) chain	2.63E-08	7.61E-07	75.88	208.89	0.36	30.25	56.33
e18580	3618.69	32.79	FAGpPGADGQPGAKGEpGDAGAKGDAGpPGPAGPAGPPGPIG	COL1A1	Collagen alpha-1(I) chain	3.53E-06	4.32E-05	43.60	119.33	0.37	22.69	43.90
e17947	3433.55	31.83	GSEGPQGVRGEPGpPGPAGAAGPAGNPGADGQPGAKGANG	COL1A1	Collagen alpha-1(I) chain	2.32E-13	4.08E-11	322.54	865.92	0.37	46.22	76.37
e17626	3343.57	31.67	pPGADGQPGAKGEpGDAGAKGDAGPpGPAGPAGPPGPIG	COL1A1	Collagen alpha-1(I) chain	3.67E-08	9.89E-07	47.96	127.74	0.38	20.17	47.37
e16845	3165.47	31.32	GADGQpGAKGEpGDAGAKGDAGPpGPAGPAGPpGPIG	COL1A1	Collagen alpha-1(I) chain	5.38E-09	2.02E-07	102.48	253.52	0.40	26.89	55.99
e16611	3109.44	36.22	RGAPGDRGEPGPPGPAGFAGPpGADGQpGAKGEP	COL1A1	Collagen alpha-1(I) chain	2.56E-05	2.62E-04	27.49	66.37	0.41	12.61	32.25
e01983	1121.49	27.63	DGRpGPpGPpGA	COL1A1	Collagen alpha-1(I) chain	3.46E-03	1.63E-02	9.97	23.97	0.42	11.76	23.52
e14876	2767.35	21.71	KEGGKGPRGETGPAGRpGEVGPpGPpGPAG	COL1A1	Collagen alpha-1(I) chain	2.12E-09	9.62E-08	100.21	238.41	0.42	35.29	64.95
e08795	1865.80	32.98	DAGPAGPKGEpGSpGENGApG	COL1A1	Collagen alpha-1(I) chain	1.46E-04	1.16E-03	30.88	72.11	0.43	18.49	36.39
e17047	3209.43	22.73	PpGESGREGApGAEGSpGRDGSpGAKGDRGETGP	COL1A1	Collagen alpha-1(I) chain	6.11E-14	1.75E-11	1363.50	3137.90	0.43	76.47	92.05
e13830	2585.15	22.96	RDGSPGAKGDRGETGPAGPPGApGApGAP	COL1A1	Collagen alpha-1(I) chain	1.27E-02	4.55E-02	12.82	29.36	0.44	4.20	12.09
e11537	2233.05	20.52	GKNGDDGEAGKPGRpGERGPpGP	COL1A1	Collagen alpha-1(I) chain	6.85E-08	1.65E-06	123.25	270.47	0.46	41.18	65.29
e17949	3434.56	31.78	GEPGPPGPAGFAGPPGADGQPGAKGEPGDAGAKGDAGPpG	COL1A1	Collagen alpha-1(I) chain	2.63E-08	7.61E-07	221.26	466.93	0.47	50.42	70.77
e05228	1466.64	28.47	GPpGSpGEQGPSGASGP	COL1A1	Collagen alpha-1(I) chain	1.18E-02	4.28E-02	18.78	39.21	0.48	12.61	22.62
e05274	1471.63	22.10	DDGEAGKpGRpGER	COL1A1	Collagen alpha-1(I) chain	9.55E-10	4.86E-08	251.50	519.77	0.48	42.02	72.34
e14445	2687.23	28.98	KDGEAGAQGPPGPAGpAGERGEQGPAGSpG	COL1A1	Collagen alpha-1(I) chain	2.37E-07	4.52E-06	53.30	108.81	0.49	24.37	50.50
e16771	3149.46	31.28	GADGQpGAKGEpGDAGAKGDAGPPGPAGPAGPpGPIG	COL1A1	Collagen alpha-1(I) chain	2.14E-09	9.62E-08	191.94	388.98	0.49	44.54	72.00
e18625	3633.74	27.04	AAGEpGKAGERGVPGpp-GAVGPAGKDGEAGAQGpPGPAGPAG	COL1A1	Collagen alpha-1(I) chain	1.19E-07	2.66E-06	83.71	164.91	0.51	21.01	49.50

Pep-tide ID	Mass [Da]	CE-time [Min]	Sequence	Symbol	Protein name	p-value	Adj. p-value (BH)	Average cases	Average controls	Fold change	Freq. cases	Freq. controls
e18536	3603.69	32.96	ADGQPGAKGEpGDAGAKGDAGPPGPAGpAGpPG-PIGNVGApG	COL1A1	Collagen alpha-1(I) chain	1.49E-03	7.97E-03	32.82	64.59	0.51	25.21	39.42
e17946	3432.59	31.95	PpGPAGFAGPPGADGQPGAKGEpGDAGAKGDAGPPGPAGP	COL1A1	Collagen alpha-1(I) chain	8.68E-10	4.67E-08	744.52	1455.89	0.51	73.11	90.93
e18740	3671.71	22.41	DKGETGEQGDRGIKGHRGFSGLQGppGPPGSPGEQGP	COL1A1	Collagen alpha-1(I) chain	1.31E-02	4.63E-02	9.04	17.35	0.52	11.76	21.39
e00467	911.44	25.83	DGKTGPpGPA	COL1A1	Collagen alpha-1(I) chain	2.80E-04	1.95E-03	161.29	309.30	0.52	43.70	59.80
e12661	2377.11	20.84	GKNGDDGEAGKPGRpGERGPpGpQ	COL1A1	Collagen alpha-1(I) chain	2.47E-10	1.67E-08	582.46	1110.93	0.52	63.03	87.68
e17221	3250.40	22.62	GPpGESGREGAPGAEGSpGRDGSPGAKGDRGETGp	COL1A1	Collagen alpha-1(I) chain	2.90E-06	3.69E-05	146.01	277.21	0.53	33.61	58.68
e05379	1486.67	23.68	DGSpGAKGDRGETGPA	COL1A1	Collagen alpha-1(I) chain	1.91E-06	2.54E-05	182.18	345.37	0.53	50.42	71.22
e16213	3022.36	24.59	SGREGApGAEGSpGRDGSPGAKGDRGETGPAGP	COL1A1	Collagen alpha-1(I) chain	2.75E-07	4.99E-06	95.56	179.02	0.53	23.53	49.72
e17596	3337.47	22.75	GPpGESGREGApGAEGSpGRDGSPGAKGDRGETGpA	COL1A1	Collagen alpha-1(I) chain	2.80E-07	5.02E-06	251.38	469.49	0.54	31.09	59.24
e18909	3736.69	32.21	PAGKDGEAGAQGPpGpAGPAGERGEQGPAG-SPGFQGLPGPAG	COL1A1	Collagen alpha-1(I) chain	4.93E-06	5.83E-05	102.22	190.83	0.54	43.70	63.72
e06176	1578.76	40.28	VGPpGPpGPPGPpGPPSA	COL1A1	Collagen alpha-1(I) chain	6.26E-03	2.61E-02	186.05	345.40	0.54	29.41	40.43
e17348	3280.45	22.62	ppGESGREGAPGAEGSpGRDGSpGAKGDRGETGPA	COL1A1	Collagen alpha-1(I) chain	1.11E-12	1.70E-10	184.76	335.10	0.55	27.73	65.17
e17890	3416.59	31.96	GPpGADGQPGAKGEpGDAGAKGDAGPPGpAGPAGPPGpIG	COL1A1	Collagen alpha-1(I) chain	1.44E-09	6.86E-08	686.62	1243.97	0.55	73.11	89.92
e12211	2320.08	20.79	KNGDDGEAGKpGRPGERGppGPQ	COL1A1	Collagen alpha-1(I) chain	1.32E-04	1.08E-03	57.42	103.39	0.56	27.73	45.46
e08755	1860.83	21.44	EGSpGRDGSpGAKGDRGET	COL1A1	Collagen alpha-1(I) chain	7.23E-07	1.10E-05	265.81	476.88	0.56	45.38	68.98
e11903	2281.98	35.26	GppGESGREGApGAEGSPGRDGSPG	COL1A1	Collagen alpha-1(I) chain	7.43E-03	2.95E-02	93.05	163.41	0.57	19.33	31.35
e16606	3108.46	31.19	ADGQpGAKGEpGDAGAKGDAGpPGPAGPAGPpGPIG	COL1A1	Collagen alpha-1(I) chain	5.91E-08	1.50E-06	293.56	515.33	0.57	68.91	83.65
e16548	3092.47	31.13	ADGQpGAKGEpGDAGAKGDAGPpGPAGPAGPPGPIG	COL1A1	Collagen alpha-1(I) chain	1.86E-09	8.69E-08	273.68	476.01	0.57	66.39	83.54
e16177	3013.34	22.23	ESGREGApGAEGSPGRDGSpGAKGDRGETGpA	COL1A1	Collagen alpha-1(I) chain	8.77E-10	4.67E-08	1755.75	3043.02	0.58	52.10	82.98
e05423	1491.73	39.89	VGpPGPPGpPGPPGPPS	COL1A1	Collagen alpha-1(I) chain	6.64E-04	4.05E-03	178.91	308.44	0.58	41.18	57.89
e05259	1469.67	23.62	DGQPGAKGEpGDAGAK	COL1A1	Collagen alpha-1(I) chain	1.26E-08	4.35E-07	734.20	1264.53	0.58	78.15	93.17
e18906	3735.70	32.37	GERGVQGPpGpAGpRGANGAPGNDGAKGDAGAP-GAPGSQGAPG	COL1A1	Collagen alpha-1(I) chain	5.54E-05	5.09E-04	184.37	314.29	0.59	55.46	71.11
e05369	1485.68	23.77	DGQpGAKGEpGDAGAK	COL1A1	Collagen alpha-1(I) chain	3.63E-06	4.40E-05	226.82	384.15	0.59	70.59	86.34
e11527	2232.01	33.67	IGPpGpAGApGDKGESGPSGPAGPTG	COL1A1	Collagen alpha-1(I) chain	1.76E-04	1.33E-03	58.21	97.74	0.60	25.21	43.78
e18578	3617.75	27.06	AAGEpGKAGERGVpGPP-GAVGPAGKDGEAGAQGPpGPAGPAG	COL1A1	Collagen alpha-1(I) chain	1.95E-05	2.05E-04	36.11	59.15	0.61	15.13	35.27
e03142	1247.52	22.07	DKGETGEQGDRG	COL1A1	Collagen alpha-1(I) chain	2.86E-07	5.09E-06	518.46	844.82	0.61	69.75	87.79
e05136	1453.68	23.64	DGQPGAKGEPGDAGAK	COL1A1	Collagen alpha-1(I) chain	3.86E-03	1.78E-02	27.37	44.35	0.62	25.21	37.85
e07275	1697.74	31.08	NGApGNDGAKGDAGApGApG	COL1A1	Collagen alpha-1(I) chain	7.73E-07	1.14E-05	423.82	685.09	0.62	52.94	76.71
e05353	1483.66	22.63	GPpGKNGDDGEAGKpG	COL1A1	Collagen alpha-1(I) chain	5.08E-06	5.97E-05	211.02	340.27	0.62	51.26	68.98
e13065	2446.09	28.29	ADGQpGAKGEpGDAGAKGDAGPpGPAGP	COL1A1	Collagen alpha-1(I) chain	1.82E-06	2.44E-05	133.12	213.21	0.62	36.97	64.50
e14893	2770.25	29.27	GPpGADGQpGAKGEpGDAGAKGDAGpPGPAGP	COL1A1	Collagen alpha-1(I) chain	1.29E-02	4.57E-02	31.17	49.84	0.63	26.05	37.07
e11002	2156.97	22.19	AEGSpGRDGSpGAKGDRGETGPA	COL1A1	Collagen alpha-1(I) chain	4.75E-07	7.72E-06	377.17	601.75	0.63	50.42	74.58
e18627	3634.70	32.70	FAGpPGADGQPGAKGEpGDAGAKGDAGPpGPAGPAGPpGPIG	COL1A1	Collagen alpha-1(I) chain	3.43E-03	1.62E-02	53.96	84.66	0.64	14.29	27.88
e16474	3076.46	31.08	ADGQPGAKGEpGDAGAKGDAGpPGPAGPAGPPGPIG	COL1A1	Collagen alpha-1(I) chain	2.04E-03	1.04E-02	27.57	43.00	0.64	12.61	26.32
e17280	3264.57	25.70	AAGEpGKAGERGVpGPpGAVGPAGKDGEAGAQGPPGP	COL1A1	Collagen alpha-1(I) chain	2.66E-08	7.61E-07	733.37	1114.14	0.66	69.75	90.26
e12949	2423.09	27.70	LDGAKGDAGPAGpKGEpGSpGENGApG	COL1A1	Collagen alpha-1(I) chain	8.04E-05	7.00E-04	145.89	221.55	0.66	55.46	73.80

Pep-tide ID	Mass [Da]	CE-time [Min]	Sequence	Symbol	Protein name	p-value	Adj. p-value (BH)	Average cases	Average controls	Fold change	Freq. cases	Freq. controls
e16170	3011.39	29.69	LTGSpGSpGpDGKTGPPGPAGQDGRPGPpGppG	COL1A1	Collagen alpha-1(I) chain	9.75E-06	1.09E-04	1008.58	1493.95	0.68	89.92	94.62
e07807	1750.79	23.87	GpPGpPGKNGDDGEAGKpG	COL1A1	Collagen alpha-1(I) chain	3.08E-05	3.08E-04	500.93	740.77	0.68	54.62	74.36
e11777	2265.98	33.80	ANGApGNDGAKGDAGApGApGSQGAPG	COL1A1	Collagen alpha-1(I) chain	5.85E-04	3.65E-03	210.81	309.47	0.68	53.78	69.54
e06309	1594.76	40.28	VGPpGPpGPpGPpGPPSA	COL1A1	Collagen alpha-1(I) chain	1.83E-04	1.36E-03	708.57	1032.95	0.69	69.75	82.08
e11264	2194.95	33.38	NGApGNDGAKGDAGAPGApGSQGApG	COL1A1	Collagen alpha-1(I) chain	1.19E-03	6.57E-03	64.85	94.36	0.69	23.53	38.97
e10032	2014.89	21.88	EGSpGRDGSpGAKGDRGETGP	COL1A1	Collagen alpha-1(I) chain	1.22E-07	2.72E-06	1441.26	2086.25	0.69	78.15	93.39
e11415	2216.03	33.83	IGPpGPAGApGDKGESGPSGPAGPTG	COL1A1	Collagen alpha-1(I) chain	1.21E-06	1.71E-05	323.80	467.94	0.69	57.14	74.36
e13825	2584.24	35.08	LTGPIGppGPAGAPGDKGESGPSGPAGPTG	COL1A1	Collagen alpha-1(I) chain	3.14E-06	3.93E-05	363.10	510.26	0.71	53.78	75.92
e05551	1507.73	40.19	VGPpGPPGPpGPpGPPS	COL1A1	Collagen alpha-1(I) chain	2.44E-03	1.20E-02	1416.21	1971.36	0.72	78.15	86.00
e13277	2488.11	27.95	PpGADGQpGAKGEpGDAGAKGDAGPpGP	COL1A1	Collagen alpha-1(I) chain	6.21E-04	3.81E-03	81.22	112.67	0.72	30.25	49.61
e05682	1523.73	40.32	VGPpGPpGPpGPpGPPS	COL1A1	Collagen alpha-1(I) chain	7.40E-03	2.95E-02	4233.04	5871.64	0.72	78.15	84.21
e17505	3318.52	30.97	AGRpGEVGPpGPpGPAGEKGSPGADGPAGAPGTpGPQG	COL1A1	Collagen alpha-1(I) chain	1.08E-05	1.19E-04	154.75	213.52	0.72	47.06	70.32
e03025	1235.55	26.86	GQDGRpGPpGpPG	COL1A1	Collagen alpha-1(I) chain	1.99E-03	1.02E-02	1011.25	1384.35	0.73	60.50	73.57
e13342	2503.13	28.32	GADGQpGAKGEpGDAGAKGDAGPpGPAGP	COL1A1	Collagen alpha-1(I) chain	1.26E-02	4.51E-02	36.28	49.63	0.73	19.33	30.68
e10483	2076.95	21.79	GPpGPpGKNGDDGEAGKpGRpG	COL1A1	Collagen alpha-1(I) chain	1.59E-04	1.23E-03	657.94	897.20	0.73	60.50	78.05
e15778	2926.30	22.17	ESGREGAPGAEGSPGRDGSpGAKGDRGETGp	COL1A1	Collagen alpha-1(I) chain	3.61E-06	4.40E-05	274.52	369.30	0.74	44.54	72.56
e15863	2942.30	22.19	ESGREGApGAEGSpGRDGSpGAKGDRGETGP	COL1A1	Collagen alpha-1(I) chain	3.66E-06	4.41E-05	1822.49	2448.32	0.74	79.83	94.51
e11991	2294.03	28.29	DGAKGDAGPAGPKGEpGSpGENGApG	COL1A1	Collagen alpha-1(I) chain	4.26E-03	1.93E-02	26.39	35.30	0.75	9.24	20.83
e15333	2841.26	24.45	GAPGpQGFQGppGEPGEPGASGPMGPRGPPG	COL1A1	Collagen alpha-1(I) chain	1.02E-05	1.14E-04	714.17	954.91	0.75	65.55	91.38
e11375	2210.96	33.69	NGApGNDGAKGDAGApGApGSQGApG	COL1A1	Collagen alpha-1(I) chain	2.28E-04	1.64E-03	1916.39	2548.84	0.75	74.79	87.79
e11213	2188.98	26.86	ADGQPGAKGEpGDAGAKGDAGPPGP	COL1A1	Collagen alpha-1(I) chain	1.49E-04	1.18E-03	497.40	646.73	0.77	62.18	81.75
e12851	2407.09	27.69	LDGAKGDAGPAGPKGEpGSpGENGApG	COL1A1	Collagen alpha-1(I) chain	1.46E-04	1.16E-03	470.90	609.78	0.77	68.07	86.23
e17438	3302.51	30.84	AGRpGEVGpPGpPGPAGEKGSPGADGPAGAPGTPGPQG	COL1A1	Collagen alpha-1(I) chain	2.10E-05	2.20E-04	210.63	272.02	0.77	44.54	66.18
e01758	1096.48	26.17	ApGDRGEpGPp	COL1A1	Collagen alpha-1(I) chain	4.75E-03	2.11E-02	2288.46	2954.48	0.77	73.11	84.55
e03890	1321.59	28.38	ApGDRGEpGPpGPA	COL1A1	Collagen alpha-1(I) chain	4.65E-03	2.08E-02	5422.22	6992.30	0.78	83.19	89.25
e19279	3871.78	27.55	QGpRGSEGPQGVRGEPGPPGPAGAAGPAGNPGADGQP-GAKGANG	COL1A1	Collagen alpha-1(I) chain	9.83E-03	3.69E-02	279.16	353.73	0.79	67.23	80.74
e05844	1542.69	24.01	DGQpGAKGEpGDAGAKG	COL1A1	Collagen alpha-1(I) chain	7.03E-03	2.83E-02	28.18	35.70	0.79	7.56	17.13
e10657	2101.91	22.00	AEGSpGRDGSpGAKGDRGETGp	COL1A1	Collagen alpha-1(I) chain	1.24E-06	1.73E-05	607.67	768.39	0.79	55.46	85.44
e08320	1809.82	20.97	GPpGKNGDDGEAGKpGRpG	COL1A1	Collagen alpha-1(I) chain	1.34E-02	4.68E-02	68.41	86.19	0.79	28.57	41.21
e17419	3295.53	25.47	KGESGPSGPAGPTGARGAPGDRGEPGPpGpAGFAGPP	COL1A1	Collagen alpha-1(I) chain	3.64E-05	3.55E-04	195.95	246.77	0.79	40.34	67.41
e10266	2047.92	21.93	NGDDGEAGKpGRpGERGPPGP	COL1A1	Collagen alpha-1(I) chain	7.51E-04	4.48E-03	7907.75	9659.54	0.82	94.96	98.77
e12366	2339.00	34.00	GANGApGNDGAKGDAGApGApGSQGApG	COL1A1	Collagen alpha-1(I) chain	7.05E-03	2.83E-02	325.19	396.47	0.82	48.74	63.27
e11536	2233.01	22.45	NGDDGEAGKPGRpGERGPpGPQG	COL1A1	Collagen alpha-1(I) chain	1.66E-04	1.26E-03	313.53	381.90	0.82	45.38	69.54
e07823	1752.78	19.61	PpGKNGDDGEAGKpGRpG	COL1A1	Collagen alpha-1(I) chain	4.78E-04	3.10E-03	59.01	71.78	0.82	26.05	43.34
e10554	2085.93	21.99	EGSpGRDGSpGAKGDRGETGPA	COL1A1	Collagen alpha-1(I) chain	1.00E-03	5.68E-03	5389.29	6529.83	0.83	92.44	98.21
e14211	2644.23	21.20	GPpGKNGDDGEAGKPGRpGERGPpGpQ	COL1A1	Collagen alpha-1(I) chain	9.53E-03	3.59E-02	308.23	370.94	0.83	55.46	72.12
e19885	4169.93	33.60	ERGEQGPAGSpGFQGLpGpAGppGEAGKpGEQGVPGDL-GAPGPSG	COL1A1	Collagen alpha-1(I) chain	1.08E-02	4.00E-02	1537.09	1842.68	0.83	83.19	91.38
e09408	1933.88	21.64	GDDGEAGKpGRPGERGPPGp	COL1A1	Collagen alpha-1(I) chain	6.73E-04	4.09E-03	745.75	882.75	0.84	64.71	88.80

Pep-tide ID	Mass [Da]	CE-time [Min]	Sequence	Symbol	Protein name	p-value	Adj. p-value (BH)	Average cases	Average controls	Fold change	Freq. cases	Freq. controls
e02530	1179.52	26.93	pGDRGEpGPpGP	COL1A1	Collagen alpha-1(I) chain	7.61E-03	3.00E-02	207.35	242.15	0.86	35.29	54.42
e03723	1306.56	27.33	GQDGRPGppGpPGA	COL1A1	Collagen alpha-1(I) chain	4.39E-03	1.97E-02	1216.91	1417.79	0.86	71.43	86.45
e05651	1519.69	22.58	GQDGRPGppGpPGARG	COL1A1	Collagen alpha-1(I) chain	1.66E-03	8.74E-03	101.26	117.81	0.86	37.82	57.00
e18806	3696.77	27.01	ARGNDGATGAAGPpGPTGPAGpPGFpGAVGAKGEAG-pQGPRG	COL1A1	Collagen alpha-1(I) chain	1.58E-05	1.68E-04	195.59	225.58	0.87	40.34	68.53
e17381	3287.49	30.80	GpAGpRGANGApGNDGAKGDAGAPGAPGSQGAPGLQGM	COL1A1	Collagen alpha-1(I) chain	1.44E-02	4.96E-02	2321.29	2662.85	0.87	84.03	95.74
e13322	2500.18	20.94	GPpGKNGDDGEAGKpGRpGERGPPGP	COL1A1	Collagen alpha-1(I) chain	1.31E-02	4.63E-02	81.78	92.36	0.89	39.50	54.42
e08916	1876.87	22.17	DDGEAGKPGRpGERGpPGP	COL1A1	Collagen alpha-1(I) chain	6.24E-03	2.61E-02	1213.24	1352.87	0.90	79.83	94.18
e17288	3266.43	22.62	GPpGESGREGAPGAEGSpGRDGSpGAKGDRGETGp	COL1A1	Collagen alpha-1(I) chain	1.11E-02	4.08E-02	2632.14	2933.76	0.90	85.71	94.18
e13200	2471.16	34.76	TGPIGpPGPAGAPGDKGESGpSGPAGPTG	COL1A1	Collagen alpha-1(I) chain	1.10E-02	4.06E-02	211.02	233.05	0.91	52.94	65.06
e10883	2137.99	25.72	NSGEPGApGSKGDTGAKGEPGPVG	COL1A1	Collagen alpha-1(I) chain	6.67E-03	2.74E-02	103.96	112.69	0.92	37.82	56.33
e11193	2185.99	25.94	NSGEpGApGSKGDTGAkGEpGPVG	COL1A1	Collagen alpha-1(I) chain	1.86E-03	9.64E-03	1181.59	1270.81	0.93	65.55	89.36
e11128	2176.03	20.48	KNGDDGEAGKPGRPGERGPpGp	COL1A1	Collagen alpha-1(I) chain	1.41E-03	7.59E-03	19.45	20.87	0.93	11.76	25.87
e09059	1892.87	22.13	DDGEAGKPGRpGERGpPGp	COL1A1	Collagen alpha-1(I) chain	5.41E-03	2.33E-02	318.33	335.20	0.95	52.94	75.03
e09542	1949.89	21.61	GDDGEAGKpGRpGERGpPGP	COL1A1	Collagen alpha-1(I) chain	8.04E-04	4.71E-03	250.26	243.76	1.03	47.90	70.77
e03533	1286.54	29.33	DGQpGAKGEpGDAG	COL1A1	Collagen alpha-1(I) chain	1.23E-02	4.41E-02	73.35	70.23	1.04	23.53	37.29
e12101	2308.01	27.33	ADGQpGAKGEpGDAGAKGDAGPpGpA	COL1A1	Collagen alpha-1(I) chain	2.01E-03	1.03E-02	166.78	156.59	1.07	33.61	54.20
e09125	1899.87	21.34	SpGRDGSpGAKGDRGETGPA	COL1A1	Collagen alpha-1(I) chain	6.47E-03	2.66E-02	190.40	173.38	1.10	36.13	53.19
e01511	1065.50	25.57	GPDGKTGpPGPA	COL1A1	Collagen alpha-1(I) chain	4.78E-03	2.12E-02	56.48	51.30	1.10	10.92	23.40
e11940	2287.01	22.41	GESGREGApGAEGSpGRDGSpGAKG	COL1A1	Collagen alpha-1(I) chain	1.43E-02	4.95E-02	43.01	37.59	1.14	15.13	26.32
e14190	2639.29	21.44	KEGGKGPRGETGPAGRpGEVGPpGPpGP	COL1A1	Collagen alpha-1(I) chain	1.07E-05	1.19E-04	389.91	322.90	1.21	47.90	75.48
e14501	2697.23	29.13	pPGADGQPGAKGEpGDAGAKGDAGpPGPAGP	COL1A1	Collagen alpha-1(I) chain	1.17E-02	4.25E-02	111.98	89.15	1.26	33.61	50.17
e06697	1635.79	40.52	VGPpGpPGPpGPPGPPSAG	COL1A1	Collagen alpha-1(I) chain	1.20E-02	4.34E-02	704.95	535.61	1.32	56.30	43.11
e05705	1526.69	23.89	DGQPGAKGEpGDAGAKG	COL1A1	Collagen alpha-1(I) chain	1.38E-02	4.79E-02	73.55	55.57	1.32	26.89	41.55
e13223	2476.17	22.83	GSpGPAGPKGSpGEAGRpGEAGLpGAKG	COL1A1	Collagen alpha-1(I) chain	4.39E-03	1.97E-02	79.77	60.26	1.32	32.77	22.28
e06859	1651.79	40.75	VGpPGpPGPpGPpGPPSAG	COL1A1	Collagen alpha-1(I) chain	2.80E-03	1.36E-02	2431.82	1745.61	1.39	69.75	55.77
e17213	3248.56	25.78	AAGEPGKAGERGVpGpPGAVGPAGKDGEAGAQGPPGP	COL1A1	Collagen alpha-1(I) chain	2.84E-03	1.37E-02	914.33	627.05	1.46	64.71	86.34
e14583	2713.23	29.12	pPGADGQpGAKGEpGDAGAKGDAGPpGPAGP	COL1A1	Collagen alpha-1(I) chain	4.18E-03	1.90E-02	44.46	30.02	1.48	14.29	27.32
e20139	4343.06	25.14	VGPpGPpGpPGPPGPPSAGFDFSFLPQPPQEKAHDGGRYYRA	COL1A1	Collagen alpha-1(I) chain	5.38E-04	3.41E-03	37.91	25.28	1.50	21.01	10.08
e19111	3802.73	32.21	ANGApGNDGAKGDAGApGApGSQGApGLQGMpGERGAA-GLPGp	COL1A1	Collagen alpha-1(I) chain	1.38E-04	1.12E-03	1200.80	761.83	1.58	80.67	66.41
e16895	3177.44	22.62	pPGESGREGAPGAEGSPGRDGSpGAKGDRGETGP	COL1A1	Collagen alpha-1(I) chain	3.43E-07	5.86E-06	99.56	61.94	1.61	11.76	36.06
e12488	2352.04	26.81	KGDRGETGpAGPPGApGAPGAPGPVGP	COL1A1	Collagen alpha-1(I) chain	3.72E-03	1.73E-02	1093.24	650.23	1.68	86.55	92.61
e05174	1458.62	28.04	SpGENGApGQmGPRG	COL1A1	Collagen alpha-1(I) chain	1.15E-02	4.17E-02	294.18	169.23	1.74	68.07	64.84
e13817	2583.20	28.30	AGPpGAPGApGAPGpVGPAGKSGDRGETGP	COL1A1	Collagen alpha-1(I) chain	5.08E-04	3.26E-03	1167.30	657.32	1.78	88.24	91.49
e10135	2029.94	21.76	SGNAGPpGPpGPAGKEGGKGPRG	COL1A1	Collagen alpha-1(I) chain	1.22E-02	4.40E-02	391.15	219.18	1.78	49.58	66.97
e18791	3689.76	20.75	LQGMpGERGAAGLPGpKGDRGDAGPKGADGSpGKDGVRG	COL1A1	Collagen alpha-1(I) chain	3.99E-05	3.84E-04	114.38	62.66	1.83	15.13	5.26
e19228	3847.85	27.44	PGLPGPSGEpGKQGpSGASGERGPPGPMGPPGLAGppGESGR	COL1A1	Collagen alpha-1(I) chain	3.07E-06	3.88E-05	308.03	149.75	2.06	68.07	52.97
e04405	1376.68	20.00	PpGPAGKEGGKGPRG	COL1A1	Collagen alpha-1(I) chain	2.45E-03	1.21E-02	1060.92	502.87	2.11	45.38	34.04
e10002	2009.96	24.86	GPSGEpGKQGpSGASGERGpPG	COL1A1	Collagen alpha-1(I) chain	6.67E-06	7.76E-05	349.68	162.35	2.15	47.06	29.79

Pep-tide ID	Mass [Da]	CE-time [Min]	Sequence	Symbol	Protein name	p-value	Adj. p-value (BH)	Average cases	Average controls	Fold change	Freq. cases	Freq. controls
e16638	3115.45	25.06	QGPGGPpGPKGNSGEpGApGSKGDTGAKGEPGpVG	COL1A1	Collagen alpha-1(I) chain	1.23E-04	1.01E-03	113.52	50.84	2.23	25.21	12.65
e09923	1997.91	25.16	NSGEPGApGSKGDTGAKGEpGP	COL1A1	Collagen alpha-1(I) chain	8.99E-03	3.45E-02	581.27	259.73	2.24	52.10	48.82
e03111	1244.56	21.70	GRDGSpGAKGDRG	COL1A1	Collagen alpha-1(I) chain	2.32E-03	1.15E-02	57.78	23.35	2.47	16.81	30.80
e18831	3703.71	22.41	DKGETGEQGDRGIKGHRGFSGLQGppGppGSPGEQGP	COL1A1	Collagen alpha-1(I) chain	1.40E-04	1.13E-03	3532.74	1418.12	2.49	72.27	73.57
e16605	3108.44	20.64	RGPpGPpGKNGDDGEAGKPGRpGERGPpGPQG	COL1A1	Collagen alpha-1(I) chain	5.48E-03	2.36E-02	87.39	33.45	2.61	21.85	12.88
e16036	2979.40	29.96	LTGSpGSpGPDGKTGPpGPAGQDGRPGPPGPpG	COL1A1	Collagen alpha-1(I) chain	5.51E-03	2.36E-02	496.79	185.36	2.68	59.66	57.33
e15755	2923.38	20.41	RGPpGPpGKNGDDGEAGKPGRpGERGPpGP	COL1A1	Collagen alpha-1(I) chain	7.12E-10	4.37E-08	3317.48	1126.56	2.94	63.03	38.19
e15015	2790.39	20.22	PGPAGPPGEAGKPGEQGVPGDLGAPGPSGARG	COL1A1	Collagen alpha-1(I) chain	2.67E-04	1.87E-03	3276.63	1102.30	2.97	32.77	19.26
e09308	1921.85	31.98	SAGPpGATGFpGAAGRVGPpGP	COL1A1	Collagen alpha-1(I) chain	2.94E-04	2.03E-03	142.09	47.19	3.01	43.70	31.24
e05480	1498.66	29.13	GEAGKpGEQGVpGDLG	COL1A1	Collagen alpha-1(I) chain	1.55E-03	8.24E-03	327.37	107.23	3.05	45.38	33.59
e17471	3310.44	25.12	AGAPGDKGESGPSGPAGpTGARGAPGDRGEpGPpGPAG	COL1A1	Collagen alpha-1(I) chain	2.49E-04	1.76E-03	237.24	74.42	3.19	28.57	15.57
e12904	2414.15	22.94	SGNAGPpGPPGpAGKEGGKGPRGETGP	COL1A1	Collagen alpha-1(I) chain	2.90E-07	5.11E-06	738.47	228.65	3.23	48.74	28.00
e11146	2178.95	32.66	GEPGSpGENGApGQMGPRGLPGE	COL1A1	Collagen alpha-1(I) chain	5.54E-04	3.48E-03	154.69	47.19	3.28	23.53	12.43
e12542	2359.07	27.50	DGQpGAKGEPGDAGAKGDAGPpGPAGP	COL1A1	Collagen alpha-1(I) chain	4.95E-11	4.05E-09	292.32	88.48	3.30	46.22	21.05
e04274	1361.58	27.83	NSGEpGApGSKGDTG	COL1A1	Collagen alpha-1(I) chain	5.17E-03	2.25E-02	46.28	13.51	3.42	15.97	8.51
e14887	2769.31	20.18	RGPpGPpGKNGDDGEAGKpGRpGERGPP	COL1A1	Collagen alpha-1(I) chain	9.69E-06	1.09E-04	151.22	44.09	3.43	26.05	11.53
e00191	854.41	23.29	PpGERGGpG	COL1A1	Collagen alpha-1(I) chain	1.34E-02	4.68E-02	111.04	31.39	3.54	26.89	18.70
e14935	2776.31	24.06	GPpGPKGNSGEpGApGSKGDTGAKGEpGPVG	COL1A1	Collagen alpha-1(I) chain	1.46E-04	1.16E-03	253.08	70.42	3.59	55.46	42.11
e13118	2456.12	27.82	PpGADGQPGAKGEpGDAGAKGDAGPPGP	COL1A1	Collagen alpha-1(I) chain	8.07E-03	3.15E-02	91.31	24.02	3.80	16.81	9.52
e02424	1168.54	26.34	KpGEQGVPGDLG	COL1A1	Collagen alpha-1(I) chain	4.69E-08	1.24E-06	296.28	72.96	4.06	42.02	21.39
e05160	1456.70	22.56	ApGSKGDTGAKGEpGP	COL1A1	Collagen alpha-1(I) chain	1.20E-04	9.93E-04	241.16	57.26	4.21	42.02	27.44
e11918	2284.02	33.27	DGAKGDAGAPGApGSQGApGLQGmpG	COL1A1	Collagen alpha-1(I) chain	1.69E-04	1.28E-03	265.44	62.72	4.23	49.58	38.52
e14827	2759.34	23.79	GFPGpKGAAGEpGKAGERGVpGPpGAVGPAG	COL1A1	Collagen alpha-1(I) chain	7.95E-04	4.68E-03	68.54	16.16	4.24	18.49	9.07
e06870	1652.76	30.26	GSpGSpGPDGKTGppGPAG	COL1A1	Collagen alpha-1(I) chain	5.69E-03	2.41E-02	392.90	81.08	4.85	43.70	35.16
e07514	1721.81	23.71	ApGPVGPAGKSGDRGETGP	COL1A1	Collagen alpha-1(I) chain	6.15E-07	9.59E-06	151.58	30.87	4.91	38.66	20.27
e05202	1462.68	22.27	DGRpGPpGPpGARGQ	COL1A1	Collagen alpha-1(I) chain	1.31E-06	1.82E-05	995.55	200.12	4.97	36.13	19.60
e05309	1476.67	29.51	TGPIGPpGPAGApGDKG	COL1A1	Collagen alpha-1(I) chain	7.48E-03	2.96E-02	352.46	70.68	4.99	30.25	19.71
e05509	1501.73	20.17	GSpGRDGSpGAKGDRG	COL1A1	Collagen alpha-1(I) chain	7.61E-07	1.14E-05	2756.49	535.52	5.15	59.66	41.32
e14709	2738.23	28.44	GPpGADGQPGAKGEPGDAGAKGDAGPpGPAGP	COL1A1	Collagen alpha-1(I) chain	1.50E-03	8.02E-03	149.39	28.98	5.15	26.89	17.47
e06054	1566.70	29.50	GpAGNpGADGQPGAKGAN	COL1A1	Collagen alpha-1(I) chain	6.36E-03	2.63E-02	277.54	51.51	5.39	23.53	15.23
e02069	1130.49	27.54	KGEpGSpGENGA	COL1A1	Collagen alpha-1(I) chain	9.25E-03	3.51E-02	50.65	9.36	5.41	17.65	10.08
e15006	2788.28	21.65	ESGREGApGAEGSpGRDGSpGAKGDRGET	COL1A1	Collagen alpha-1(I) chain	6.69E-03	2.74E-02	54.04	9.98	5.42	12.61	6.16
e00992	993.48	24.84	VRGEpGPpGP	COL1A1	Collagen alpha-1(I) chain	1.13E-02	4.14E-02	58.67	10.63	5.52	12.61	6.61
e09562	1952.86	25.43	GEPGApGSKGDTGAKGEpGPVG	COL1A1	Collagen alpha-1(I) chain	4.69E-03	2.09E-02	173.41	30.41	5.70	19.33	11.31
e09799	1981.94	25.16	NSGEPGApGSKGDTGAKGEPGP	COL1A1	Collagen alpha-1(I) chain	3.92E-16	4.49E-13	1351.14	229.98	5.88	55.46	22.73
e08392	1816.88	24.38	SGNAGPpGPpGPAGKEGGKGP	COL1A1	Collagen alpha-1(I) chain	7.19E-07	1.10E-05	296.73	49.79	5.96	19.33	6.49
e01025	998.48	21.21	DGEAGKpGRP	COL1A1	Collagen alpha-1(I) chain	1.87E-03	9.69E-03	31.03	5.12	6.06	17.65	9.18
e09953	2002.94	25.44	GQPGAKGEpGDAGAKGDAGPPGP	COL1A1	Collagen alpha-1(I) chain	5.43E-04	3.43E-03	101.08	16.36	6.18	11.76	4.37
e08234	1798.88	41.05	VGPpGPpGPpGPpGPPSAGF	COL1A1	Collagen alpha-1(I) chain	4.53E-07	7.52E-06	1038.75	164.46	6.32	19.33	6.38
e13730	2567.20	28.23	AGPpGAPGApGAPGPVGPAGKSGDRGETGP	COL1A1	Collagen alpha-1(I) chain	3.37E-07	5.84E-06	2406.05	380.52	6.32	82.35	79.17

Pep-tide ID	Mass [Da]	CE-time [Min]	Sequence	Symbol	Protein name	p-value	Adj. p-value (BH)	Average cases	Average controls	Fold change	Freq. cases	Freq. controls
e13648	2554.14	23.13	GPpGESGREGApGAEGSpGRDGSpGAKG	COL1A1	Collagen alpha-1(I) chain	3.83E-03	1.77E-02	195.24	30.59	6.38	20.17	11.53
e05415	1490.74	22.88	EpGKAGERGVPGPPGA	COL1A1	Collagen alpha-1(I) chain	4.98E-03	2.18E-02	104.57	16.19	6.46	16.81	9.18
e06985	1667.79	40.64	TGDAGpVGPpGPpGPPGPP	COL1A1	Collagen alpha-1(I) chain	6.95E-04	4.20E-03	139.55	20.89	6.68	10.92	4.03
e00383	893.44	23.55	GPpGPpGARG	COL1A1	Collagen alpha-1(I) chain	2.98E-05	2.99E-04	5.97	0.88	6.77	11.76	3.47
e09978	2006.91	32.30	GpAGPpGEAGKpGEQGVpGDLG	COL1A1	Collagen alpha-1(I) chain	4.13E-11	3.50E-09	688.38	99.43	6.92	36.97	14.45
e07720	1742.85	23.74	LGApGPSGARGERGFpGE	COL1A1	Collagen alpha-1(I) chain	1.42E-08	4.64E-07	421.41	60.35	6.98	28.57	10.97
e08522	1833.88	24.05	GEPGKQGPSGASGERGPpGP	COL1A1	Collagen alpha-1(I) chain	3.70E-11	3.26E-09	8608.69	1204.19	7.15	35.29	12.88
e14018	2612.26	23.41	GPpGPAGEEGKRGARGEPGpTGLpGPpG	COL1A1	Collagen alpha-1(I) chain	9.36E-03	3.54E-02	150.07	20.08	7.48	24.37	16.57
e12792	2398.11	22.80	GNAGPpGPpGPAGKEGGKGPRGETGPA	COL1A1	Collagen alpha-1(I) chain	2.44E-04	1.74E-03	268.53	32.73	8.20	13.45	5.15
e10219	2042.93	32.24	PGAVGPAGKDGEAGAQGPpGpAGP	COL1A1	Collagen alpha-1(I) chain	8.87E-07	1.29E-05	75.03	8.93	8.40	14.29	3.92
e00271	870.48	23.82	DGVRGLTGP	COL1A1	Collagen alpha-1(I) chain	1.59E-04	1.23E-03	210.37	24.93	8.44	24.37	12.43
e08666	1849.86	24.75	GEPGKQGpSGASGERGPpGP	COL1A1	Collagen alpha-1(I) chain	3.63E-03	1.69E-02	724.28	80.23	9.03	25.21	16.13
e06027	1563.75	23.05	GEpGKAGERGVPGPpGA	COL1A1	Collagen alpha-1(I) chain	1.37E-06	1.88E-05	729.54	77.38	9.43	44.54	26.43
e02899	1221.58	26.91	IGPpGPAGApGDKG	COL1A1	Collagen alpha-1(I) chain	6.14E-04	3.80E-03	198.06	21.00	9.43	22.69	12.32
e03982	1331.62	27.87	RpGEVGPpGPpGPA	COL1A1	Collagen alpha-1(I) chain	6.35E-05	5.77E-04	203.79	18.36	11.10	23.53	11.20
e15680	2907.38	20.45	RGPpGPPGKNGDDGEAGKPGRPGERGPpGp	COL1A1	Collagen alpha-1(I) chain	5.60E-04	3.51E-03	1128.87	99.15	11.39	31.93	20.04
e14750	2745.33	21.49	ERGSPGPAGPKGSpGEAGRpGEAGLpGAKG	COL1A1	Collagen alpha-1(I) chain	6.91E-06	7.99E-05	305.19	26.05	11.71	32.77	17.36
e12709	2384.08	22.89	GApGAEGSpGRDGSpGAKGDRGETGP	COL1A1	Collagen alpha-1(I) chain	2.87E-08	8.07E-07	183.03	15.15	12.08	17.65	4.82
e09896	1993.98	21.54	GPKGSpGEAGRpGEAGLpGAKG	COL1A1	Collagen alpha-1(I) chain	7.63E-04	4.53E-03	331.60	23.58	14.06	21.01	11.09
e03905	1322.60	27.62	GSpGPDGKTGPpGPA	COL1A1	Collagen alpha-1(I) chain	1.01E-03	5.71E-03	1559.31	73.74	21.15	21.01	11.76
e11048	2165.00	21.93	ERGSpGpAGpKGSPGEAGRPGEA	COL1A1	Collagen alpha-1(I) chain	4.73E-07	7.72E-06	1092.69	48.14	22.70	18.49	6.05
e09075	1893.91	21.22	NGDDGEAGKPGRpGERGPp	COL1A1	Collagen alpha-1(I) chain	1.06E-04	8.91E-04	466.46	16.11	28.95	18.49	7.84
e16540	3091.46	28.24	GPAGAAGARGNDGQPGPAGPPGPVGpAGGpGFpGAPG	COL2A1	Collagen alpha-1(II) chain	1.36E-11	1.48E-09	50.44	212.99	0.24	19.33	53.30
e18281	3530.65	26.13	ppGSNGNpGPPGPPGPSGKDGPKGARGDSGPPGRAGEPG	COL2A1	Collagen alpha-1(II) chain	2.24E-06	2.93E-05	85.71	255.65	0.34	26.05	49.05
e11713	2256.97	33.66	GETGAAGpPGPAGPAGERGEQGAPGP	COL2A1	Collagen alpha-1(II) chain	7.43E-05	6.62E-04	219.08	440.47	0.50	32.77	52.63
e06794	1645.76	20.76	DGPKGASGPAGPpGAQGpP	COL2A1	Collagen alpha-1(II) chain	1.27E-05	1.37E-04	25.51	48.09	0.53	23.53	44.79
e07685	1738.76	24.95	GPAGApGpQGFQGNpGEPG	COL2A1	Collagen alpha-1(II) chain	1.87E-07	3.72E-06	196.39	367.00	0.54	39.50	66.74
e09547	1950.84	32.03	GPpGEGGKpGDQGVpGEAGApG	COL2A1	Collagen alpha-1(II) chain	7.65E-03	3.01E-02	14.44	26.70	0.54	11.76	22.73
e05675	1523.62	23.69	GSPGPAGASGNpGTDGIP	COL2A1	Collagen alpha-1(II) chain	4.04E-08	1.08E-06	90.65	167.03	0.54	36.13	64.50
e04199	1353.66	25.88	PVGpSGKDGANGIpG	COL2A1	Collagen alpha-1(II) chain	2.22E-06	2.93E-05	69.61	125.51	0.55	27.73	54.31
e12658	2376.95	33.92	AGppGEKGEPGDDGpSGAEGPpGPQG	COL2A1	Collagen alpha-1(II) chain	2.82E-04	1.95E-03	114.24	195.12	0.59	29.41	46.70
e16656	3121.43	30.16	FAGpPGADGQpGAKGEQGEAGQKGDAGApGpQGP	COL2A1	Collagen alpha-1(II) chain	1.37E-08	4.55E-07	378.87	622.21	0.61	53.78	84.10
e18044	3458.58	31.40	AGGPGFPGApGAKGEAGpTGARGpEGAQGPRGEPGTPGS	COL2A1	Collagen alpha-1(II) chain	8.74E-08	2.04E-06	9323.34	13797.94	0.68	82.35	95.07
e04531	1392.63	21.80	PGTpGSPGPAGASGNPG	COL2A1	Collagen alpha-1(II) chain	4.94E-05	4.62E-04	2289.92	3288.88	0.70	89.08	95.97
e17184	3242.48	22.89	GPAGFAGPPGADGQPGAKGEQGEAGQKGDAGAPGPQG	COL2A1	Collagen alpha-1(II) chain	5.57E-06	6.51E-05	141.05	200.73	0.70	36.13	59.24
e12714	2385.05	33.96	pAGPPGEKGEPGDDGPSGAEGPpGPQ	COL2A1	Collagen alpha-1(II) chain	7.27E-04	4.37E-03	306.99	433.05	0.71	57.98	73.01
e01661	1084.43	25.18	DGpSGAEGpPGp	COL2A1	Collagen alpha-1(II) chain	1.22E-06	1.72E-05	1257.52	1760.05	0.71	72.27	90.82
e12007	2296.95	27.05	pGADGQpGAKGEQGEAGQKGDAGAp	COL2A1	Collagen alpha-1(II) chain	2.11E-04	1.53E-03	177.15	241.79	0.73	24.37	42.78
e17377	3286.53	25.40	GARGAPGERGETGPPGpAGFAGpPGADGQPGAKGEQ	COL2A1	Collagen alpha-1(II) chain	4.29E-03	1.94E-02	95.27	129.79	0.73	31.93	47.26

Pep-tide ID	Mass [Da]	CE-time [Min]	Sequence	Symbol	Protein name	p-value	Adj. p-value (BH)	Average cases	Average controls	Fold change	Freq. cases	Freq. controls
e18922	3740.67	32.25	FAGppGADGQPGAKGEQGEAGQKG-DAGAPGPQGPSGAPGPQG	COL2A1	Collagen alpha-1(II) chain	1.62E-03	8.54E-03	88.15	118.59	0.74	42.02	57.89
e19780	4113.85	24.55	GAPGERGETGPpGpAGFAGpPGADGQPGAKGEQGEAGQKG-DAGAPG	COL2A1	Collagen alpha-1(II) chain	1.34E-07	2.89E-06	270.06	360.11	0.75	44.54	71.22
e13634	2551.15	34.75	PGVKGESGSpGENGSpGpMGPRGLPGE	COL2A1	Collagen alpha-1(II) chain	6.19E-04	3.81E-03	84.73	108.73	0.78	23.53	40.99
e13033	2442.07	34.10	GpAGPPGEKGEPGDDGPSGAEGPpGPQ	COL2A1	Collagen alpha-1(II) chain	4.93E-03	2.17E-02	779.94	964.10	0.81	72.27	85.44
e13127	2458.08	34.20	GpAGpPGEKGEPGDDGPSGAEGpPGPQ	COL2A1	Collagen alpha-1(II) chain	1.33E-02	4.67E-02	32.12	36.27	0.89	15.13	26.09
e04196	1353.59	21.57	PSGDQGASGpAGPSGP	COL2A1	Collagen alpha-1(II) chain	7.38E-03	2.95E-02	228.35	162.65	1.40	50.42	67.19
e20089	4306.00	25.07	SPGADGPPGRDGAAGVKGDRGETGAVGAPGAPGPpG-SpGpAGPTGKQGD	COL2A1	Collagen alpha-1(II) chain	1.70E-04	1.28E-03	1846.64	1168.97	1.58	70.59	56.33
e09072	1893.84	32.04	pPGEGGKpGDQGVpGEAGApG	COL2A1	Collagen alpha-1(II) chain	3.59E-04	2.42E-03	92.38	50.25	1.84	23.53	12.09
e19022	3774.72	22.71	ERGETGPPGPAGFAGPpGADGQpGAKGEQGEAGQKGDAGAp	COL2A1	Collagen alpha-1(II) chain	9.84E-12	1.19E-09	5876.69	2260.94	2.60	74.79	49.38
e17318	3274.47	22.99	GpAGFAGPPGADGQPGAKGEQGEAGQKGDAGAPGPQG	COL2A1	Collagen alpha-1(II) chain	8.86E-03	3.41E-02	218.28	47.57	4.59	5.04	14.11
e15593	2889.36	24.09	GpSGpAGARGIQGpQGPRGDKGEAGEPGER	COL2A1	Collagen alpha-1(II) chain	1.45E-02	4.98E-02	385.56	65.55	5.88	8.40	18.59
e05303	1475.78	22.81	GRpGPpGPQGARGQp	COL2A1	Collagen alpha-1(II) chain	1.58E-08	4.97E-07	583.49	54.84	10.64	26.05	9.07
e06181	1579.65	39.92	GETGAVGApGApGPpGSPG	COL2A1	Collagen alpha-1(II) chain	2.40E-04	1.72E-03	42.80	1.92	22.28	10.92	3.70
e06611	1626.72	30.52	EGGKpGDQGVpGEAGApG	COL2A1	Collagen alpha-1(II) chain	7.53E-03	2.97E-02	1414.06	32.10	44.05	5.04	14.00
e12451	2347.04	21.91	SPGVSGpKGDAGQpGEKGSPGAQGPP	COL3A1	Collagen alpha-1(III) chain	1.38E-02	4.78E-02	1.87	9.81	0.19	3.36	10.30
e20018	4255.98	25.73	FPGSPGAKGEVGPAGSPGSNGAPGQRGE-PGPQGHAGAQGPpGppGIN	COL3A1	Collagen alpha-1(III) chain	8.07E-11	6.00E-09	104.21	522.08	0.20	11.76	43.56
e01767	1097.50	21.08	PGpTGPGGDkGD	COL3A1	Collagen alpha-1(III) chain	3.63E-03	1.69E-02	5.13	22.42	0.23	2.52	11.20
e17112	3226.48	22.73	PSGSPGKDGPpGpAGNTGAPGSPGVSGPKGDAGQPGE	COL3A1	Collagen alpha-1(III) chain	7.75E-03	3.04E-02	3.48	13.56	0.26	3.36	11.31
e16231	3025.40	29.89	pPGKNGETGPQGppGPTGPGGDKGDTGPPGpQG	COL3A1	Collagen alpha-1(III) chain	9.27E-05	7.89E-04	11.09	41.81	0.27	5.88	21.28
e18864	3718.72	32.42	GpAGPPGPPGPPGTSGHPGSPGSPGYQGPPGEPGQAGPSGPPG	COL3A1	Collagen alpha-1(III) chain	5.59E-13	9.14E-11	116.62	383.80	0.30	37.82	69.76
e01648	1082.50	23.81	GpEGGKGAAGPpG	COL3A1	Collagen alpha-1(III) chain	1.23E-04	1.01E-03	45.43	129.02	0.35	21.85	38.63
e14821	2758.25	28.98	KNGETGPQGppGPTGPGGDKGDTGPpGPQG	COL3A1	Collagen alpha-1(III) chain	7.38E-08	1.74E-06	32.55	86.06	0.38	20.17	47.03
e15129	2809.20	24.38	ERGEAGIpGVpGAKGEDGKDGSPGEpGANG	COL3A1	Collagen alpha-1(III) chain	1.09E-11	1.25E-09	164.64	390.81	0.42	31.93	69.09
e01935	1115.51	21.60	DGESGRpGRpG	COL3A1	Collagen alpha-1(III) chain	2.51E-07	4.68E-06	78.69	167.08	0.47	28.57	54.87
e17270	3262.50	22.36	APGAPGHPGPPGPVGPAGKSGDRGESGPAGPAGAPGPAG	COL3A1	Collagen alpha-1(III) chain	2.56E-03	1.25E-02	23.24	49.12	0.47	14.29	26.65
e08188	1794.80	24.01	GNDGApGKNGERGGpGGpGP	COL3A1	Collagen alpha-1(III) chain	1.70E-11	1.69E-09	546.54	1127.58	0.48	70.59	89.47
e06841	1649.73	22.75	GPpGpAGQpGDKGEGGAPG	COL3A1	Collagen alpha-1(III) chain	8.90E-11	6.18E-09	387.63	792.57	0.49	68.91	85.78
e08165	1790.81	23.85	GESGRPGppGpSGPRGQPG	COL3A1	Collagen alpha-1(III) chain	3.05E-07	5.33E-06	71.15	139.82	0.51	33.61	59.46
e03938	1326.54	29.12	SpGGpGSDGKpGPpG	COL3A1	Collagen alpha-1(III) chain	4.70E-03	2.09E-02	28.79	55.35	0.52	10.08	20.83
e17170	3239.49	30.85	NTGApGSPGVSGpKGDAGQpGEKGSPGAQGPPGAPGP	COL3A1	Collagen alpha-1(III) chain	9.97E-03	3.73E-02	26.49	49.67	0.53	21.01	31.13
e17866	3409.61	31.29	NTGApGSpGVSGPKGDAGQPGEKGSpGAQGPPGAPGPLG	COL3A1	Collagen alpha-1(III) chain	1.06E-06	1.53E-05	164.18	296.60	0.55	41.18	64.50
e15411	2854.37	34.67	PQGPpGPTGpGGDKGDTGPpGPQGLQGLpGT	COL3A1	Collagen alpha-1(III) chain	1.27E-08	4.35E-07	1170.70	2075.98	0.56	69.75	88.02
e16388	3058.39	24.82	GSPGKDGppGpAGNTGAPGSPGVSGPKGDAGQPGE	COL3A1	Collagen alpha-1(III) chain	2.88E-06	3.68E-05	103.64	180.77	0.57	21.85	46.47
e17238	3255.49	30.76	NTGApGSpGVSGPKGDAGQpGEKGSpGAQGPPGAPGP	COL3A1	Collagen alpha-1(III) chain	2.02E-07	3.92E-06	162.21	282.86	0.57	45.38	70.88
e14315	2663.21	23.57	NRGERGSEGSPGHpGQpGPPGpPGApGP	COL3A1	Collagen alpha-1(III) chain	1.54E-05	1.65E-04	189.46	313.82	0.60	48.74	72.68
e14224	2647.21	23.52	NRGERGSEGSPGHpGQPGPpGPPGApGP	COL3A1	Collagen alpha-1(III) chain	7.41E-03	2.95E-02	35.27	57.68	0.61	19.33	31.13

Peptide ID	Mass [Da]	CE-time [Min]	Sequence	Symbol	Protein name	p-value	Adj. p-value (BH)	Average cases	Average controls	Fold change	Freq. cases	Freq. controls
e17254	3258.47	22.83	ENGKPGEpGpKGDAGApGApGGKGDAGApGERGpPG	COL3A1	Collagen alpha-1(III) chain	2.88E-09	1.20E-07	1067.62	1745.05	0.61	67.23	92.39
e14644	2726.26	28.92	KNGETGPQGPPGPTGPGGDKGDTGPpGPQG	COL3A1	Collagen alpha-1(III) chain	2.53E-05	2.59E-04	110.01	176.11	0.62	40.34	61.03
e13662	2557.17	28.23	KNGETGPQGPPGpTGPGGDKGDTGPpGP	COL3A1	Collagen alpha-1(III) chain	1.50E-07	3.16E-06	83.64	133.64	0.63	26.05	54.09
e12496	2353.99	27.28	AGIpGVpGAKGEDGKDGSpGEpGANG	COL3A1	Collagen alpha-1(III) chain	8.33E-06	9.49E-05	86.64	136.96	0.63	28.57	52.97
e18041	3457.61	31.46	NTGAPGSpGVSGpKGDAGQpGEKGSpGAQGppGAPGPLG	COL3A1	Collagen alpha-1(III) chain	1.79E-08	5.40E-07	11914.80	18291.13	0.65	83.19	94.40
e09629	1962.88	31.97	QGLpGTGGPpGENGKpGEpGP	COL3A1	Collagen alpha-1(III) chain	6.30E-03	2.62E-02	63.16	96.33	0.66	36.13	48.26
e09856	1989.88	32.53	SNGNPGppGPSGSpGKDGPPGp	COL3A1	Collagen alpha-1(III) chain	1.17E-04	9.75E-04	154.97	234.43	0.66	42.02	63.94
e13697	2563.15	21.25	ApGPAGSRGApGPQGpRGDKGETGERG	COL3A1	Collagen alpha-1(III) chain	3.55E-07	6.03E-06	678.20	1019.52	0.67	66.39	89.14
e18092	3473.60	31.47	NTGApGSpGVSGpKGDAGQpGEKGSpGAQGpPGAPGpLG	COL3A1	Collagen alpha-1(III) chain	1.69E-07	3.40E-06	2587.84	3888.82	0.67	84.87	94.29
e16132	3001.44	34.97	YDVKSGVAVGGLAGYpGpAGPPGPPGPPGTSGH	COL3A1	Collagen alpha-1(III) chain	4.86E-05	4.58E-04	5512.16	8257.45	0.67	82.35	92.61
e16085	2991.41	20.97	SPGGPGAAGFPGARGLPGPPGSNGNPGPPGPSGSp	COL3A1	Collagen alpha-1(III) chain	8.64E-03	3.34E-02	118.96	176.06	0.68	42.86	57.78
e17922	3425.61	31.29	NTGApGSPGVSGPKGDAGQpGEKGSpGAQGpPGAPGPLG	COL3A1	Collagen alpha-1(III) chain	5.47E-07	8.76E-06	1594.62	2348.01	0.68	82.35	94.62
e11780	2266.03	22.25	QNGEpGGKGERGApGEKGEGGPpG	COL3A1	Collagen alpha-1(III) chain	7.66E-07	1.14E-05	2716.01	3928.14	0.69	93.28	97.20
e02160	1140.48	21.09	DKGDTGPPGPQG	COL3A1	Collagen alpha-1(III) chain	4.94E-05	4.62E-04	1301.13	1818.67	0.72	78.99	91.71
e17311	3271.52	30.76	NTGApGSpGVSGpKGDAGQpGEKGSPGAQGPPGAPGp	COL3A1	Collagen alpha-1(III) chain	6.90E-07	1.07E-05	756.09	1043.56	0.72	76.47	91.27
e02041	1127.52	20.81	ApGKNGERGGpG	COL3A1	Collagen alpha-1(III) chain	1.36E-02	4.73E-02	16.63	22.79	0.73	23.53	36.17
e16220	3023.41	20.91	SPGGPGAAGFPGARGLPGPPGSNGNpGppGPSGSP	COL3A1	Collagen alpha-1(III) chain	3.55E-05	3.48E-04	252.46	341.90	0.74	42.02	69.99
e15237	2825.28	24.45	ERGEAGIpGVpGAKGEDGKDGSpGEpGANG	COL3A1	Collagen alpha-1(III) chain	4.84E-05	4.58E-04	9479.35	12183.74	0.78	95.80	98.99
e10881	2137.93	21.77	NGEpGGKGERGAPGEKGEGGppG	COL3A1	Collagen alpha-1(III) chain	1.00E-03	5.68E-03	410.45	524.63	0.78	63.87	77.94
e10257	2046.92	32.75	GSNGNpGpPGPSGSpGKDGPpGP	COL3A1	Collagen alpha-1(III) chain	3.89E-04	2.59E-03	1245.56	1558.11	0.80	66.39	87.12
e07676	1737.78	23.79	NDGAPGKNGERGGpGGpGp	COL3A1	Collagen alpha-1(III) chain	2.55E-04	1.80E-03	2568.59	3209.83	0.80	89.92	96.86
e09891	1993.88	32.19	SEGSPGHpGQPGpPGpPGApGP	COL3A1	Collagen alpha-1(III) chain	1.34E-02	4.68E-02	185.71	230.51	0.81	49.58	61.37
e15323	2839.36	24.20	GPAGPRGPVGPSGPPGKDGTSGHPGPIGppGP	COL3A1	Collagen alpha-1(III) chain	2.52E-06	3.28E-05	574.73	702.42	0.82	68.91	83.99
e14735	2742.25	28.93	KNGETGPQGPPGpTGPGGDKGDTGPpGPQG	COL3A1	Collagen alpha-1(III) chain	9.62E-03	3.62E-02	1386.97	1693.26	0.82	89.92	95.86
e20087	4305.93	28.79	ARGNDGARGSDGQpGpPGPpGTAGFpGSpGAKGEVGpAG-SpGSNGApG	COL3A1	Collagen alpha-1(III) chain	5.22E-03	2.27E-02	2448.30	2973.08	0.82	89.08	94.96
e04649	1405.70	23.33	DGVPGKDGPRGPTGP	COL3A1	Collagen alpha-1(III) chain	7.04E-03	2.83E-02	90.96	109.56	0.83	42.86	55.21
e17856	3405.56	25.85	ARGNDGARGSDGQPGPpGppGTAGFpGSpGAKGEVGP	COL3A1	Collagen alpha-1(III) chain	1.77E-04	1.33E-03	1924.16	2316.69	0.83	72.27	92.16
e06220	1583.70	23.30	NDGApGKNGERGGpGGp	COL3A1	Collagen alpha-1(III) chain	1.42E-07	3.02E-06	170.44	194.53	0.88	42.02	66.18
e14253	2654.20	23.89	ERGEAGIpGVpGAKGEDGKDGSpGEpGA	COL3A1	Collagen alpha-1(III) chain	2.29E-05	2.38E-04	189.63	201.39	0.94	36.13	61.14
e06598	1624.73	24.42	NDGApGKNGERGGpGGPG	COL3A1	Collagen alpha-1(III) chain	1.44E-02	4.96E-02	226.93	218.43	1.04	23.53	36.39
e10386	2062.93	26.46	DAGAPGApGGKGDAGApGERGpPG	COL3A1	Collagen alpha-1(III) chain	9.28E-04	5.30E-03	373.28	358.57	1.04	55.46	76.60
e03194	1251.62	22.45	DGVPGKDGPRGPT	COL3A1	Collagen alpha-1(III) chain	4.24E-03	1.92E-02	272.28	249.61	1.09	45.38	61.14
e14956	2779.23	28.94	HRGFpGNPGAPGSPGPAGQqGAIGSpGPAGP	COL3A1	Collagen alpha-1(III) chain	6.32E-03	2.62E-02	71.08	59.60	1.19	15.97	29.12
e01923	1114.49	25.71	SpGERGETGPp	COL3A1	Collagen alpha-1(III) chain	3.24E-03	1.53E-02	3320.91	2588.47	1.28	74.79	91.71
e20132	4338.00	25.14	LQGLPGTGGppGENGKpGEpGpKGDAGApGApGGKG-DAGApGERGpPG	COL3A1	Collagen alpha-1(III) chain	2.18E-04	1.57E-03	991.05	728.76	1.36	70.59	55.21
e13816	2583.17	23.63	ERGEAGIpGVpGAKGEDGKDGSpGEpG	COL3A1	Collagen alpha-1(III) chain	3.10E-05	3.09E-04	275.19	184.79	1.49	36.13	61.14
e19159	3822.88	24.17	QQGAIGSPGPAGPRGPVGpSGpPGKDGTSGHpGPIGPPGpRG	COL3A1	Collagen alpha-1(III) chain	1.52E-04	1.19E-03	62.84	41.23	1.52	21.01	9.52

Pep-tide ID	Mass [Da]	CE-time [Min]	Sequence	Symbol	Protein name	p-value	Adj. p-value (BH)	Average cases	Average controls	Fold change	Freq. cases	Freq. controls
e20109	4321.99	25.14	LQGLpGTGGppGENGKpGEPGpKGDAGAPGAPGGKG-DAGApGERGppG	COL3A1	Collagen alpha-1(III) chain	6.56E-08	1.62E-06	10890.25	6544.08	1.66	87.39	76.04
e05577	1510.67	23.44	GGAGEpGKNGAKGEpGP	COL3A1	Collagen alpha-1(III) chain	1.89E-03	9.77E-03	248.28	137.29	1.81	17.65	32.36
e19801	4121.91	23.26	GEPGRDGVPGGPGMRGMPGSPGG-PGSDGKPGPpGSQGESGRpGpP	COL3A1	Collagen alpha-1(III) chain	5.41E-03	2.33E-02	2821.21	1312.82	2.15	41.18	31.13
e17824	3400.55	30.92	GEVGPAGSPGSNGApGQRGEpGpQGHAGAQGPPGPPGI	COL3A1	Collagen alpha-1(III) chain	3.67E-04	2.48E-03	515.32	232.61	2.22	43.70	28.56
e16173	3012.38	22.19	LGSPGpKGDKGEpGGpGADGVPGKDGPRGPTGP	COL3A1	Collagen alpha-1(III) chain	9.46E-04	5.39E-03	681.50	306.11	2.23	53.78	43.00
e12891	2412.11	27.18	RGGAGPPGpEGGKGAAGPpGpPGAAGTpG	COL3A1	Collagen alpha-1(III) chain	4.48E-03	2.01E-02	653.57	293.39	2.23	71.43	70.10
e05814	1538.78	20.42	GEpGKNGAKGEPGpRG	COL3A1	Collagen alpha-1(III) chain	2.82E-03	1.37E-02	133.97	59.19	2.26	31.93	21.16
e03633	1296.61	21.80	RGApGEKGEGGpPG	COL3A1	Collagen alpha-1(III) chain	3.10E-03	1.48E-02	56.43	23.70	2.38	29.41	18.81
e19548	4008.81	23.32	GEPGRDGVPGGPGMRGMPGSPGG-PGSDGKPGPPGSQGESGRpGp	COL3A1	Collagen alpha-1(III) chain	1.47E-08	4.66E-07	10437.78	4272.68	2.44	69.75	50.50
e11599	2241.01	33.65	PpGSNGNpGPpGPSGSPGKDGPpGP	COL3A1	Collagen alpha-1(III) chain	5.53E-03	2.36E-02	102.91	41.18	2.50	16.81	9.29
e19321	3890.78	24.34	SNGNPGPPGPSGSPGKDGPPGpAGNTGApGSpGVSGPKG-DAGQPG	COL3A1	Collagen alpha-1(III) chain	2.83E-15	1.62E-12	2909.11	941.85	3.09	80.67	56.44
e05918	1551.69	29.71	GTGGPpGENGKpGEpGP	COL3A1	Collagen alpha-1(III) chain	1.16E-04	9.68E-04	640.59	199.55	3.21	68.91	59.57
e15095	2802.31	19.60	AGERGAPGFRGPAGPNGIPGEKGpAGERGA	COL3A1	Collagen alpha-1(III) chain	3.93E-03	1.81E-02	31.45	8.79	3.58	15.97	7.95
e04723	1412.62	29.58	SpGAPGApGHpGPpGP	COL3A1	Collagen alpha-1(III) chain	9.82E-04	5.58E-03	44.24	12.05	3.67	11.76	4.59
e09966	2004.93	25.02	GARGNDGARGSDGQPGPpGPpG	COL3A1	Collagen alpha-1(III) chain	5.69E-10	3.72E-08	799.24	216.68	3.69	69.75	51.74
e10019	2012.95	25.18	RGGAGPpGPEGGKGAAGPpGPpGA	COL3A1	Collagen alpha-1(III) chain	3.92E-06	4.68E-05	99.57	26.27	3.79	36.97	20.60
e00184	852.42	23.26	DGPRGPTGP	COL3A1	Collagen alpha-1(III) chain	2.09E-03	1.06E-02	22.18	5.70	3.89	15.13	7.05
e10046	2016.02	21.43	GIpGEKGPAGERGApGPAGPRG	COL3A1	Collagen alpha-1(III) chain	7.62E-04	4.53E-03	4267.02	1018.91	4.19	66.39	67.64
e14434	2684.27	21.39	GEpGRDGNpGSDGLpGRDGSpGGKGDRG	COL3A1	Collagen alpha-1(III) chain	9.30E-03	3.53E-02	118.28	26.91	4.40	36.97	29.23
e03927	1325.56	21.47	GEpGKNGAKGEpGP	COL3A1	Collagen alpha-1(III) chain	8.51E-04	4.95E-03	201.41	43.33	4.65	20.17	35.61
e05434	1492.71	22.74	GESGRpGPpGPSGPRG	COL3A1	Collagen alpha-1(III) chain	6.08E-04	3.77E-03	76.05	16.30	4.66	23.53	12.77
e02000	1123.50	26.03	GGAGPpGPEGGKGA	COL3A1	Collagen alpha-1(III) chain	3.92E-04	2.60E-03	106.03	20.36	5.21	21.01	10.53
e08819	1867.92	21.32	ApGPQGpRGDKGETGERGA	COL3A1	Collagen alpha-1(III) chain	1.12E-05	1.23E-04	297.05	56.18	5.29	24.37	10.75
e00837	968.45	26.63	KGDTGPpGPQ	COL3A1	Collagen alpha-1(III) chain	8.13E-03	3.17E-02	49.63	9.34	5.31	10.92	5.15
e06617	1627.70	29.59	MpGSpGGpGSDGKpGpPG	COL3A1	Collagen alpha-1(III) chain	8.38E-05	7.25E-04	6322.27	1040.89	6.07	85.71	82.42
e13241	2481.12	27.35	NTGApGSpGVSGpKGDAGQPGEKGSPGA	COL3A1	Collagen alpha-1(III) chain	7.03E-08	1.68E-06	298.26	47.71	6.25	47.06	28.11
e01416	1052.48	25.16	GGAGPpGPEGGKG	COL3A1	Collagen alpha-1(III) chain	7.46E-04	4.47E-03	258.37	40.21	6.43	26.05	15.68
e04135	1347.68	21.86	GRPGppGPSGPRGQ	COL3A1	Collagen alpha-1(III) chain	3.24E-06	3.99E-05	106.39	16.02	6.64	22.69	9.07
e13362	2507.13	22.82	ApGQNGEPGGkGERGAPGEkGEGGPpG	COL3A1	Collagen alpha-1(III) chain	6.62E-04	4.05E-03	639.13	94.24	6.78	29.41	47.03
e14880	2768.26	24.19	ERGEAGIpGVpGAKGEDGKDGSpGEpGAN	COL3A1	Collagen alpha-1(III) chain	2.06E-03	1.05E-02	142.63	19.30	7.39	26.89	17.69
e12612	2369.07	34.14	PpGSNGNpGpPGPSGSpGKDGPPGPAG	COL3A1	Collagen alpha-1(III) chain	7.45E-10	4.37E-08	187.57	24.75	7.58	29.41	10.53
e03325	1265.61	21.80	ApGKNGERGGPGGP	COL3A1	Collagen alpha-1(III) chain	8.39E-11	6.00E-09	390.08	51.07	7.64	30.25	10.19
e02344	1160.54	26.41	SpGPAGPRGPVGP	COL3A1	Collagen alpha-1(III) chain	1.28E-02	4.56E-02	166.72	21.79	7.65	15.97	8.96
e14469	2691.26	24.02	IGSpGPAGPRGPVGpSGPPGKDGTSGHpGP	COL3A1	Collagen alpha-1(III) chain	3.28E-08	8.95E-07	79.72	10.00	7.97	17.65	4.82
e13352	2505.20	20.93	GEpGPRGERGEAGIPGVpGAKGEDGK	COL3A1	Collagen alpha-1(III) chain	6.84E-03	2.79E-02	169.67	20.47	8.29	15.13	7.84
e14566	2710.30	28.62	RGGAGpPGPEGGKGAAGPpGPpGAAGTpGLQG	COL3A1	Collagen alpha-1(III) chain	1.71E-08	5.22E-07	838.96	97.60	8.60	32.77	14.22

Pep-tide ID	Mass [Da]	CE-time [Min]	Sequence	Symbol	Protein name	p-value	Adj. p-value (BH)	Average cases	Average controls	Fold change	Freq. cases	Freq. controls
e06066	1567.69	29.54	NGSpGAPGApGHPGPpGP	COL3A1	Collagen alpha-1(III) chain	8.19E-06	9.38E-05	398.57	39.61	10.06	38.66	23.18
e05726	1529.70	39.89	GPpGFTGppGPPGPPGP	COL4A1	Collagen alpha-1(IV) chain	1.58E-04	1.22E-03	193.82	363.87	0.53	45.38	64.05
e05864	1545.70	40.28	GppGFTGPpGpPGPPGP	COL4A1	Collagen alpha-1(IV) chain	4.86E-04	3.14E-03	1315.50	1995.33	0.66	70.59	82.53
e17983	3441.61	31.36	DGAPGQKGEMGPAGPTGPRGFpGppGPDGLPGSMGPP	COL4A1	Collagen alpha-1(IV) chain	1.23E-07	2.72E-06	6488.86	9407.16	0.69	82.35	95.41
e12114	2309.19	19.56	GEKGEKGSIGIPGMPGSPGLKGSpG	COL4A1	Collagen alpha-1(IV) chain	4.27E-04	2.79E-03	18.98	9.65	1.97	12.61	4.70
e18981	3759.86	19.39	GMpGVGEKGEpGKPGPRGKPGKDGDKGEKGSPGFPGEPG	COL4A1	Collagen alpha-1(IV) chain	2.84E-03	1.37E-02	4743.92	1851.78	2.56	51.26	43.45
e05900	1549.69	40.72	QGpQGEKGEAGppGPP	COL4A1	Collagen alpha-1(IV) chain	1.05E-02	3.91E-02	82.58	30.49	2.71	17.65	10.08
e16099	2995.38	29.72	TpSQTTDERGPPGEQGPPGPPGPPGVPGIDG	COL9A1	Collagen alpha-1(IX) chain	2.68E-11	2.55E-09	38.52	120.41	0.32	17.65	51.51
e06111	1572.67	39.90	pGADGLTGPDGSpGSIGS	COL9A1	Collagen alpha-1(IX) chain	5.85E-03	2.47E-02	30.47	23.17	1.31	21.85	12.65
e09264	1916.85	24.65	LpGAPGDQGQRGPpGEAGPK	COL9A1	Collagen alpha-1(IX) chain	8.65E-04	5.00E-03	70.96	11.49	6.17	20.17	10.53
e08146	1787.93	24.63	LGRVGPVGDpGRRGppGP	COL9A1	Collagen alpha-1(IX) chain	7.50E-04	4.48E-03	95.94	8.92	10.76	15.13	6.83
e01721	1092.46	36.37	GPpGPpGpAGEp	COL9A1	Collagen alpha-1(IX) chain	7.79E-03	3.05E-02	258.07	18.14	14.23	18.49	10.53
e18967	3756.69	32.37	GPPGEPGPAGQDGPPGDKGDDGEPGQTGSPGPTGEPGPSGPP	COL5A1	Collagen alpha-1(V) chain	2.51E-07	4.68E-06	84.97	168.65	0.50	43.70	66.07
e18060	3463.59	30.87	PGEKGETGDVGQMGPPGPPGPRGpSGApGADGpQGPPG	COL5A1	Collagen alpha-1(V) chain	9.17E-05	7.84E-04	842.68	1278.51	0.66	67.23	82.64
e05011	1441.68	39.08	pGPPGEPGPAGQDGPP	COL5A1	Collagen alpha-1(V) chain	3.12E-03	1.48E-02	391.40	546.14	0.72	26.05	41.77
e19013	3772.71	32.53	GpPGEPGPAGQDGPPGDKGDDGEPGQTGSPGPTGEPGPSGPP	COL5A1	Collagen alpha-1(V) chain	1.94E-04	1.43E-03	78.16	107.00	0.73	26.89	46.36
e18346	3549.51	31.23	DpGPPGEPGPAGQDGPPGDKGDDGEPGQTGSPGPTGEPG	COL5A1	Collagen alpha-1(V) chain	1.71E-03	8.94E-03	434.00	502.91	0.86	57.14	71.44
e06015	1562.69	22.48	GApGADGpQGpPGGIGNP	COL5A1	Collagen alpha-1(V) chain	5.23E-03	2.27E-02	1733.44	1633.26	1.06	84.87	93.39
e12971	2428.09	27.47	VVGpQGPTGETGpMGERGHpGPpGP	COL5A1	Collagen alpha-1(V) chain	1.27E-02	4.55E-02	38.34	29.97	1.28	12.61	24.30
e03053	1238.54	27.56	GEAGHPGPpGPPGP	COL5A1	Collagen alpha-1(V) chain	9.40E-05	7.97E-04	86.81	40.22	2.16	36.13	20.60
e09005	1885.84	32.28	PGppGpRGPSGAPGADGpQGP	COL5A1	Collagen alpha-1(V) chain	1.01E-02	3.77E-02	62.03	27.11	2.29	21.85	13.66
e11963	2291.01	33.46	ppGpPGVVGpQGPTGETGPMGERG	COL5A1	Collagen alpha-1(V) chain	1.01E-07	2.33E-06	152.46	61.16	2.49	23.53	8.40
e11907	2282.05	25.63	GADGPPGHPGKEGPpGEKGGqGppG	COL5A1	Collagen alpha-1(V) chain	8.78E-04	5.05E-03	98.77	26.23	3.76	14.29	6.16
e16400	3060.53	21.10	PQGPTGETGPMGERGHpGpPGPPGEQGLPGLA	COL5A1	Collagen alpha-1(V) chain	1.46E-08	4.66E-07	49.35	10.73	4.60	24.37	7.95
e12651	2375.21	22.51	KGGQGpPGPQGpIGYPGPRGVKGAD	COL5A1	Collagen alpha-1(V) chain	2.72E-05	2.76E-04	34095.59	6628.88	5.14	44.54	28.67
e17131	3231.42	22.51	PPGDPGLMGERGEDGpAGNGTEGFpGFPGYPGN	COL6A1	Collagen alpha-1(VI) chain	8.67E-09	3.20E-07	82.16	212.17	0.39	32.77	60.58
e17202	3247.38	22.46	PpGDpGLMGERGEDGpAGNGTEGFPGFPGYPGN	COL6A1	Collagen alpha-1(VI) chain	2.73E-09	1.17E-07	305.87	632.88	0.48	38.66	67.08
e17637	3347.45	30.65	EAGRpGSSGpSGDEGQPGEPGPPGEKGEAGDEGNPG	COL6A1	Collagen alpha-1(VI) chain	7.27E-04	4.37E-03	44.18	85.44	0.52	24.37	40.87
e19631	4043.79	33.01	GEpGADGEAGRpGSSGpSGDEGQPGEPGPPGEKGEAGDEGNP GP	COL6A1	Collagen alpha-1(VI) chain	2.65E-06	3.41E-05	233.81	387.36	0.60	52.10	74.24
e17705	3363.44	30.78	EAGRpGSSGpSGDEGQpGEPGPPGEKGEAGDEGNPG	COL6A1	Collagen alpha-1(VI) chain	2.19E-03	1.10E-02	155.08	230.82	0.67	36.13	54.09
e20326	4467.97	28.94	GSEGARGAPGPAGPPGDPGLMGERGEDGPAGNG-TEGFpGFpGYpGNR	COL6A1	Collagen alpha-1(VI) chain	5.02E-04	3.23E-03	160.14	236.31	0.68	55.46	73.68
e19757	4100.77	33.06	PGADGEAGRPGSSGpSGDEGQpGEpGPPGEKGEAGDEGNPGP DGA	COL6A1	Collagen alpha-1(VI) chain	1.03E-04	8.67E-04	129.91	185.16	0.70	32.77	53.98
e16718	3136.41	30.22	DPGLMGERGEDGpAGNGTEGFpGFPGYPGNR	COL6A1	Collagen alpha-1(VI) chain	3.94E-04	2.61E-03	124.00	154.65	0.80	24.37	45.02
e17447	3304.39	22.57	GPpGDpGLMGERGEDGpAGNGTEGFPGFPGYPGN	COL6A1	Collagen alpha-1(VI) chain	1.79E-04	1.34E-03	491.68	593.84	0.83	52.94	75.81
e16281	3037.34	22.30	GADGEAGRPGSSGPSGDEGQPGEpGppGEKGEA	COL6A1	Collagen alpha-1(VI) chain	4.00E-05	3.84E-04	388.84	440.23	0.88	42.86	70.88
e11761	2263.91	33.50	ADGEAGRPGSSGPSGDEGQPGEPGP	COL6A1	Collagen alpha-1(VI) chain	1.86E-03	9.64E-03	31.75	9.45	3.36	12.61	5.38
e02718	1200.54	26.31	GEKGEAGDpGRp	COL6A1	Collagen alpha-1(VI) chain	3.17E-06	3.95E-05	526.59	93.88	5.61	29.41	13.66

Pep-tide ID	Mass [Da]	CE-time [Min]	Sequence	Symbol	Protein name	p-value	Adj. p-value (BH)	Average cases	Average controls	Fold change	Freq. cases	Freq. controls
e16018	2975.44	20.97	QGPPGSATAKGERGFPGADGRpGSPGRAGNPG	COL7A1	Collagen alpha-1(VII) chain	6.46E-04	3.96E-03	10.45	38.76	0.27	12.61	26.54
e20262	4414.02	28.99	FDGQPGPKGDQGEKGERGTPGIGGFPGPSGNDGSAG-PPGPpGSVGpR	COL7A1	Collagen alpha-1(VII) chain	1.69E-08	5.22E-07	67.04	162.45	0.41	23.53	53.19
e16156	3007.42	21.01	QGPpGSATAKGERGFpGADGRpGSPGRAGNPG	COL7A1	Collagen alpha-1(VII) chain	3.18E-05	3.15E-04	202.78	322.08	0.63	46.22	68.87
e05288	1473.63	22.33	FPGQTGPRGEMGQp	COL7A1	Collagen alpha-1(VII) chain	8.02E-04	4.71E-03	237.84	343.28	0.69	74.79	84.66
e20082	4304.04	23.05	GDRGFPGPLGEAGEKGERGPPGPAGSRGLpGVAGRp-GAKGpEGPPG	COL7A1	Collagen alpha-1(VII) chain	2.42E-04	1.73E-03	51.46	17.21	2.99	21.01	9.97
e13387	2511.14	27.32	KGEKGDSEDGApGLpGQPGSpGEQGp	COL7A1	Collagen alpha-1(VII) chain	1.70E-03	8.92E-03	220.43	64.20	3.43	43.70	34.83
e14783	2751.35	21.71	LPGQVGETGKpGApGRDGASGKDGDRGSP	COL7A1	Collagen alpha-1(VII) chain	3.78E-04	2.54E-03	118.24	29.77	3.97	13.45	5.26
e09719	1971.94	24.77	RGDPGPQGppGLALGERGpP	COL7A1	Collagen alpha-1(VII) chain	9.00E-03	3.45E-02	870.05	207.60	4.19	46.22	38.07
e11417	2216.08	22.36	KGEKGDSGASGREGFPGVpGGTGP	COL7A1	Collagen alpha-1(VII) chain	1.08E-13	2.24E-11	2764.09	538.29	5.13	48.74	20.60
e00748	954.49	24.73	KGDKGEAGPP	COL7A1	Collagen alpha-1(VII) chain	7.63E-05	6.73E-04	44.86	6.59	6.81	15.13	5.71
e03037	1236.56	27.18	GDRGEpGpPGPpG	COL7A1	Collagen alpha-1(VII) chain	5.36E-08	1.40E-06	195.91	26.51	7.39	24.37	8.73
e07939	1764.83	24.47	pGpLGEAGEKGERGpPGp	COL7A1	Collagen alpha-1(VII) chain	3.47E-04	2.35E-03	91.43	9.89	9.25	12.61	4.82
e07215	1691.79	31.85	GSPGppGpAGIATKGLNGP	COL10A1	Collagen alpha-1(X) chain	9.31E-03	3.53E-02	15.74	32.05	0.49	3.36	11.31
e07224	1692.77	23.47	GIpGFPGSKGDPGSPGPPG	COL10A1	Collagen alpha-1(X) chain	4.01E-04	2.65E-03	224.84	160.34	1.40	59.66	46.92
e07216	1691.81	23.67	KGDPGSPGPpGpAGIATKG	COL10A1	Collagen alpha-1(X) chain	3.42E-07	5.86E-06	62.12	4.11	15.13	14.29	3.70
e02502	1176.56	26.86	PGPQGPTGpSGpP	COL10A1	Collagen alpha-1(X) chain	2.92E-08	8.07E-07	87.10	3.62	24.04	14.29	3.25
e02940	1226.53	21.11	SGGDGppGpPGER	COL11A1	Collagen alpha-1(XI) chain	1.95E-12	2.63E-10	175.00	446.62	0.39	41.18	75.03
e18973	3757.74	32.85	PGEPGpAGQDGVGGDKGEDGDpGQPGPPGPSGEAGPPGPPGK	COL11A1	Collagen alpha-1(XI) chain	7.76E-03	3.04E-02	19.10	42.43	0.45	9.24	19.15
e12945	2423.00	33.98	DGpQGPpGSVGSVGGVGEKGEPGEAGN	COL11A1	Collagen alpha-1(XI) chain	1.97E-04	1.45E-03	101.17	149.24	0.68	36.13	52.41
e07389	1708.78	32.15	GpAGEKGApGEKGpQGPAG	COL11A1	Collagen alpha-1(XI) chain	4.96E-03	2.18E-02	191.11	271.70	0.70	41.18	54.20
e04847	1425.59	22.34	GGDkGEDGDPGQPGp	COL11A1	Collagen alpha-1(XI) chain	9.64E-06	1.09E-04	1496.82	2101.28	0.71	75.63	92.27
e17283	3265.44	35.90	GPAGQDGVGGDKGEDGDpGQpGPPGPSGEAGPPGpPG	COL11A1	Collagen alpha-1(XI) chain	8.36E-03	3.25E-02	201.77	254.40	0.79	50.42	63.16
e05053	1445.67	28.49	GPMGPPGppGpRGPQ	COL11A1	Collagen alpha-1(XI) chain	4.68E-04	3.05E-03	442.57	178.38	2.48	68.07	60.36
e17634	3346.67	19.25	EGQSGEKGALGppGpQGPIGYPGPRGVKGADGVRG	COL11A1	Collagen alpha-1(XI) chain	2.97E-03	1.43E-02	166.79	44.10	3.78	27.73	17.92
e02106	1134.60	21.20	pGPpGKRGPpGA	COL11A1	Collagen alpha-1(XI) chain	2.19E-09	9.65E-08	571.81	117.82	4.85	17.65	4.26
e16701	3131.45	31.20	QGPpGPqGpNGLSIPGEQGRQGmKGDAGEPGL	COL12A1	Collagen alpha-1(XII) chain	5.65E-03	2.40E-02	18.34	31.76	0.58	10.92	22.51
e16294	3039.40	34.39	QPGDKGERGAAGEQGpDGpKGSKGEPGKGEM	COL13A1	Collagen alpha-1(XIII) chain	6.38E-03	2.63E-02	2502.06	2883.20	0.87	75.63	85.22
e07594	1730.77	30.12	PGHPGpKGDMGLTGpPGQ	COL13A1	Collagen alpha-1(XIII) chain	1.16E-02	4.20E-02	571.73	362.33	1.58	73.11	69.88
e07106	1681.74	30.91	EAGEKGNPGAEGLQGVpG	COL13A1	Collagen alpha-1(XIII) chain	1.02E-03	5.73E-03	1111.95	469.34	2.37	55.46	42.11
e08610	1844.91	41.11	SQASIQGppGPPGPpGPSGp	COL13A1	Collagen alpha-1(XIII) chain	1.24E-09	6.05E-08	512.04	104.66	4.89	18.49	4.48
e17892	3417.50	22.03	KGERGAAGEQGpDGPKGSKGEPGKGEMVDYNGNI	COL13A1	Collagen alpha-1(XIII) chain	2.69E-05	2.73E-04	57.00	9.57	5.96	10.92	3.02
e15200	2820.31	23.85	GIpGGVGSpGRDGSpGQRGLPGKDGSSGPpG	COL14A1	Collagen alpha-1(XIV) chain	4.15E-07	6.94E-06	509.07	215.42	2.36	83.19	74.36
e17139	3232.52	22.12	KGEKGNpGVGTQGPRGPPGPAGPSGESRPGSPGPP	COL14A1	Collagen alpha-1(XIV) chain	3.36E-04	2.29E-03	79.40	22.31	3.56	15.97	6.83
e06381	1603.74	30.02	VQGppGEPGRPGSpGAP	COL14A1	Collagen alpha-1(XIV) chain	7.23E-03	2.90E-02	335.23	74.14	4.52	49.58	43.78
e15611	2892.33	34.39	QKGEQGFEGSKGETGEKGEQGEKGDpAL	COL19A1	Collagen alpha-1(XIX) chain	1.04E-07	2.37E-06	384.24	773.02	0.50	52.10	77.60
e19753	4098.99	21.17	GPEGPSGKpGINGKDGIPGAQGImGKpGDRGpKGERGDQGIP	COL19A1	Collagen alpha-1(XIX) chain	1.02E-07	2.33E-06	3599.92	1873.29	1.92	74.79	55.43
e03306	1263.56	26.70	GSDGPPGKpGppGP	COL19A1	Collagen alpha-1(XIX) chain	5.33E-04	3.38E-03	52.25	6.42	8.13	10.92	3.92
e17504	3318.40	22.73	TDVFMGPpGSpGEDGPAGEPGPPGPEGQPGVDGATG	COL15A1	Collagen alpha-1(XV) chain	1.45E-06	1.98E-05	61.49	124.23	0.49	21.01	45.58

Pep-tide ID	Mass [Da]	CE-time [Min]	Sequence	Symbol	Protein name	p-value	Adj. p-value (BH)	Average cases	Average controls	Fold change	Freq. cases	Freq. controls
e06922	1659.74	29.44	GPpGFGRpGDpGPPGPPG	COL15A1	Collagen alpha-1(XV) chain	8.80E-03	3.40E-02	1114.93	724.22	1.54	82.35	85.33
e12388	2340.21	26.00	GEKGDPGNRGLPGPPGKKGQAGPpG	COL15A1	Collagen alpha-1(XV) chain	3.41E-05	3.36E-04	722.59	381.07	1.90	26.05	12.43
e17239	3255.50	36.06	GppGLPGIPGKPGTDVFMGPPGSPGEDGpAGEpGP	COL15A1	Collagen alpha-1(XV) chain	3.88E-03	1.79E-02	62.99	24.21	2.60	21.01	11.98
e02207	1145.48	37.24	GDPGpPGPpGPpG	COL15A1	Collagen alpha-1(XV) chain	8.92E-03	3.43E-02	5.33	2.03	2.63	10.08	4.48
e07459	1715.77	30.45	FGRpGDpGPPGPPGppGP	COL15A1	Collagen alpha-1(XV) chain	5.50E-03	2.36E-02	238.55	83.24	2.87	34.45	25.31
e01845	1106.48	36.92	PGVMGpPGppGP	COL15A1	Collagen alpha-1(XV) chain	9.13E-10	4.76E-08	354.20	55.66	6.36	26.89	8.96
e05421	1491.65	23.57	HpGppGEPGTDGAAGK	COL16A1	Collagen alpha-1(XVI) chain	2.71E-04	1.89E-03	29.33	70.72	0.41	24.37	40.99
e10130	2029.85	20.34	SAGEkGEPGPPGSEGLPGppGP	COL16A1	Collagen alpha-1(XVI) chain	1.29E-03	7.03E-03	25.08	51.81	0.48	21.85	37.63
e11495	2226.96	33.46	GNSGEKGDQGFQGQPGFPGPpGP	COL16A1	Collagen alpha-1(XVI) chain	5.11E-03	2.23E-02	62.97	76.76	0.82	22.69	36.95
e15408	2854.31	21.56	pAGERGHPGAPGPSGSPGLPGVPGSMGDMVN	COL16A1	Collagen alpha-1(XVI) chain	1.55E-04	1.20E-03	109.59	119.45	0.92	30.25	52.63
e17903	3421.57	26.00	AGERGHPGApGPSGSPGLpGVPGSMGDMVNYDEIK	COL16A1	Collagen alpha-1(XVI) chain	1.51E-03	8.03E-03	532.62	563.06	0.95	45.38	68.87
e10847	2131.99	32.55	KGEPGppGQPGYPGATGPpGLPG	COL16A1	Collagen alpha-1(XVI) chain	1.62E-03	8.54E-03	222.42	76.68	2.90	46.22	35.83
e09708	1970.91	24.92	AAGKEGPPGKQGFYGPpGpK	COL16A1	Collagen alpha-1(XVI) chain	3.21E-06	3.98E-05	1422.60	367.06	3.88	63.87	48.26
e07614	1731.82	40.63	GIAGENGLPGppGPQGppG	COL16A1	Collagen alpha-1(XVI) chain	9.19E-09	3.34E-07	497.71	88.22	5.64	19.33	5.38
e06980	1666.83	23.69	GPPGPAGERGHPGApGPSG	COL16A1	Collagen alpha-1(XVI) chain	2.40E-04	1.72E-03	1555.12	206.28	7.54	43.70	31.47
e12694	2382.15	22.71	GPAGPRGERGPQGNSGEKGDQGFQ	COL16A1	Collagen alpha-1(XVI) chain	1.41E-04	1.13E-03	273.84	31.69	8.64	13.45	5.04
e08891	1874.86	24.43	QRGEEGPPGMRGSPGPpGP	COL16A1	Collagen alpha-1(XVI) chain	5.42E-11	4.28E-09	617.26	56.32	10.96	38.66	16.46
e04769	1417.64	20.06	pGPpGpKGDQGPpGP	COL17A1	Collagen alpha-1(XVII) chain	3.99E-03	1.83E-02	310.57	372.57	0.83	69.75	82.42
e06856	1651.77	23.31	PGSFLSNSETFLSGpP	COL17A1	Collagen alpha-1(XVII) chain	6.16E-03	2.59E-02	48.38	51.14	0.95	10.08	20.83
e12384	2340.04	22.58	EGpmGQRGREGPMGpRGEAGPpG	COL17A1	Collagen alpha-1(XVII) chain	1.21E-02	4.34E-02	460.78	152.38	3.02	58.82	58.68
e11512	2228.08	22.45	SGPPGPPGPPGpKGDQGpPGPRGH	COL17A1	Collagen alpha-1(XVII) chain	1.10E-02	4.06E-02	46.01	13.12	3.51	17.65	10.30
e15902	2951.44	22.27	AGEPGPHGPPGVPGSVGPKGSSGSPGPQGPPGPV	COL17A1	Collagen alpha-1(XVII) chain	1.54E-11	1.60E-09	2304.97	183.22	12.58	38.66	15.01
e13779	2577.25	24.62	DDILASPPRLPEPQPYPGAPHHSS	COL18A1	Collagen alpha-1(XVIII) chain	2.71E-03	1.32E-02	151.58	130.76	1.16	47.06	64.61
e13289	2490.24	24.64	DDILASPPRLPEPQPYPGAPHHS	COL18A1	Collagen alpha-1(XVIII) chain	3.30E-04	2.26E-03	334.60	155.70	2.15	66.39	59.13
e16025	2976.44	20.46	pAGpALQTVpGPQGPPGPPGRDGTPGRDGEP	COL18A1	Collagen alpha-1(XVIII) chain	2.79E-05	2.81E-04	93.08	37.28	2.50	24.37	11.31
e00977	990.55	20.58	NVAKGIRSF	COL18A1	Collagen alpha-1(XVIII) chain	2.11E-03	1.06E-02	55.91	17.15	3.26	12.61	5.49
e05744	1531.69	40.62	pGGGGFFGSSLpGpPGP	COL18A1	Collagen alpha-1(XVIII) chain	7.63E-05	6.73E-04	52.47	13.09	4.01	19.33	8.17
e08853	1871.83	31.17	PGDPGEDGKpGDTGpQGFp	COL18A1	Collagen alpha-1(XVIII) chain	1.06E-02	3.94E-02	239.99	34.34	6.99	19.33	11.98
e07751	1745.78	24.11	TSAESpDApEENIAGVGA	COL18A1	Collagen alpha-1(XVIII) chain	5.94E-04	3.69E-03	123.41	9.50	12.99	15.13	6.61

Pep-tide ID	Mass [Da]	CE-time [Min]	Sequence	Symbol	Protein name	p-value	Adj. p-value (BH)	Average cases	Average controls	Fold change	Freq. cases	Freq. controls
e05725	1529.69	29.50	MRGMPGpPGpPGPPGP	COL18A1	Collagen alpha-1(XVIII) chain	7.46E-03	2.96E-02	181.79	13.68	13.29	19.33	11.87
e05830	1540.74	39.81	GPpGVPGpPGpGGSPGLP	COL22A1	Collagen alpha-1(XXII) chain	3.84E-04	2.57E-03	2000.07	2966.42	0.67	78.15	86.67
e16303	3041.38	29.95	pGppGIAGPQGSQGERGADGEVGQKGDQGHPG	COL22A1	Collagen alpha-1(XXII) chain	5.40E-05	5.00E-04	920.48	1262.79	0.73	77.31	92.39
e05677	1523.67	22.09	pGKDGDTGPTGPQGPQ	COL22A1	Collagen alpha-1(XXII) chain	3.06E-03	1.46E-02	2777.91	3552.76	0.78	94.12	97.42
e05961	1556.74	40.01	pGVpGPPGPGGSPGLPGE	COL22A1	Collagen alpha-1(XXII) chain	1.21E-02	4.34E-02	455.12	510.23	0.89	50.42	64.50
e15160	2814.34	20.02	SpGSRGLPGFPGPQGPAGRDGAPGNPGERG	COL22A1	Collagen alpha-1(XXII) chain	1.55E-07	3.21E-06	401.42	342.50	1.17	28.57	11.20
e05644	1518.75	22.90	KpGPpGpTGpPGKDGP	COL22A1	Collagen alpha-1(XXII) chain	2.41E-03	1.19E-02	38.98	18.49	2.11	16.81	8.29
e20946	5508.53	25.31	GTEGKKGEAGPPGLPGPpGIAGpQGSQGERGADG-EVGQKGDQGHPGVPGFMGPPGNPGP	COL22A1	Collagen alpha-1(XXII) chain	4.14E-04	2.73E-03	637.20	280.55	2.27	43.70	31.35
e09908	1995.95	24.98	GPTGPQGPQGPRGPPGKNGSPG	COL22A1	Collagen alpha-1(XXII) chain	4.77E-15	2.18E-12	553.02	121.64	4.55	38.66	11.87
e19272	3869.82	20.69	GPPGPPGVPGPPGPGGSPGLPGEIGFPGKPGPPGpTGpPGKDGP	COL22A1	Collagen alpha-1(XXII) chain	8.79E-05	7.54E-04	1582.89	341.92	4.63	33.61	18.70
e10073	2019.96	25.52	GLpGFpGpQGPAGRDGAPGNPG	COL22A1	Collagen alpha-1(XXII) chain	2.11E-03	1.06E-02	1028.54	199.80	5.15	36.97	26.43
e05046	1444.72	28.93	GPpGKDGpNGpPGPPG	COL22A1	Collagen alpha-1(XXII) chain	3.69E-14	1.21E-11	205.86	22.66	9.09	31.09	8.62
e14558	2708.19	21.85	GKKGDDGTPSQpGppGPKGEpGSMGPRG	COL23A1	Collagen alpha-1(XXIII) chain	2.34E-05	2.42E-04	149.02	19.84	7.51	24.37	11.42
e18759	3677.85	26.69	pGRTGLAGApGpPGVKGSSGLPGSPGIQGpKGEQGLPGQP	COL24A1	Collagen alpha-1(XXIV) chain	2.38E-07	4.52E-06	530.97	191.80	2.77	49.58	29.00
e01856	1107.50	36.82	pGLQGpSGPpGP	COL24A1	Collagen alpha-1(XXIV) chain	4.38E-03	1.97E-02	14.88	5.10	2.92	10.08	4.14
e10311	2054.05	21.58	RGPKGDTGPPGPPGPAGIpGpSG	COL24A1	Collagen alpha-1(XXIV) chain	7.63E-07	1.14E-05	493.08	128.41	3.84	27.73	12.09
e19485	3981.92	22.14	GEKGVMGYPGPPGVPGpIGPLGLPGHVGARGPPGSQGPKGQR	COL24A1	Collagen alpha-1(XXIV) chain	3.35E-03	1.58E-02	3462.69	220.94	15.67	11.76	5.26
e09548	1950.85	35.51	GTDGpMGpHGpAGPKGERGE	COL25A1	Collagen alpha-1(XXV) chain	6.85E-08	1.65E-06	62.55	187.73	0.33	27.73	52.07
e04814	1422.62	22.68	KGTDGpMGPHGpAGp	COL25A1	Collagen alpha-1(XXV) chain	2.31E-03	1.15E-02	182.43	233.84	0.78	21.01	36.95
e15228	2824.28	21.50	HGPPGpmGPHGLPGPKGTDGPMGPHGpAGp	COL25A1	Collagen alpha-1(XXV) chain	1.86E-05	1.96E-04	124.01	27.91	4.44	23.53	10.64

Pep-tide ID	Mass [Da]	CE-time [Min]	Sequence	Symbol	Protein name	p-value	Adj. p-value (BH)	Average cases	Average controls	Fold change	Freq. cases	Freq. controls
e13619	2548.26	27.12	ANGMKGEKGDSGMPGPQGPSIIGPPGp	COL25A1	Collagen alpha-1(XXV) chain	8.97E-14	2.21E-11	443.08	91.11	4.86	23.53	4.82
e00142	842.37	34.86	SGMPGpQGP	COL25A1	Collagen alpha-1(XXV) chain	8.45E-03	3.28E-02	18.44	3.48	5.29	10.08	4.48
e00600	935.45	23.82	GRpGPpGPpG	COL26A1	Collagen alpha-1(XXVI) chain	1.31E-03	7.13E-03	31.89	63.31	0.50	16.81	30.80
e06279	1591.68	39.72	PGppGPEGFPGDIGpPG	COL27A1	Collagen alpha-1(XXVII) chain	1.28E-02	4.56E-02	52.26	16.99	3.08	15.13	8.51
e07469	1716.77	28.12	KGDpGDpGpPGTHGNPGI	COL28A1	Collagen alpha-1(XXVIII) chain	1.19E-03	6.57E-03	129.39	197.00	0.66	36.13	53.08
e14200	2641.26	19.60	PMGIpGIGSQGEQGIQGpIGpPGPQGPA	COL28A1	Collagen alpha-1(XXVIII) chain	2.61E-06	3.38E-05	3202.62	312.54	10.25	58.82	44.12
e10993	2154.98	33.18	FAGEKGPSGEAGTAGPpGTpGPQG	COL1A2	Collagen alpha-2(I) chain	6.17E-04	3.81E-03	4.78	20.43	0.23	4.20	16.13
e17585	3334.56	31.03	EPGSAGPQGPPGPSGEEGKRGPNGEAGSAGPPGppGL	COL1A2	Collagen alpha-2(I) chain	2.90E-08	8.07E-07	25.53	107.78	0.24	18.49	44.12
e17751	3375.57	31.83	GEPGSAGPQGPPGPSGEEGKRGPNGEAGSAGPPGpPGL	COL1A2	Collagen alpha-2(I) chain	9.63E-14	2.21E-11	165.69	568.62	0.29	47.90	78.16
e04647	1405.64	20.15	DGPpGRDGQpGHKG	COL1A2	Collagen alpha-2(I) chain	1.92E-06	2.54E-05	14.85	50.82	0.29	25.21	44.79
e13424	2518.09	21.13	EAGRDGNpGNDGPpGRDGQpGHKGE	COL1A2	Collagen alpha-2(I) chain	2.46E-03	1.21E-02	2.76	8.93	0.31	2.52	11.87
e10635	2096.93	20.27	DGppGRDGQpGHKGERGYpG	COL1A2	Collagen alpha-2(I) chain	2.17E-04	1.57E-03	3.32	10.47	0.32	5.88	20.16
e15108	2805.30	23.80	LKGQpGApGVKGEpGApGENGTPGQTGARG	COL1A2	Collagen alpha-2(I) chain	6.88E-03	2.79E-02	13.11	32.92	0.40	5.04	14.11
e06288	1592.74	19.54	EDGHpGKpGRpGERG	COL1A2	Collagen alpha-2(I) chain	2.30E-05	2.39E-04	60.84	144.24	0.42	34.45	52.63
e19541	4005.96	22.25	EVGKpGERGLHGEFGLPGPAGpRGERGpPGESGAAGPTGPIG	COL1A2	Collagen alpha-2(I) chain	1.47E-03	7.91E-03	26.53	56.09	0.47	14.29	28.44
e18867	3719.69	32.15	QGFQGPAGEPGEPGQTGPAGARGPAGppGKAGEDGHpGKP	COL1A2	Collagen alpha-2(I) chain	5.90E-08	1.50E-06	278.80	538.20	0.52	63.03	80.29
e09557	1951.86	32.09	VNGApGEAGRDGNpGNDGPpG	COL1A2	Collagen alpha-2(I) chain	7.79E-05	6.81E-04	29.66	57.07	0.52	13.45	32.92
e06154	1576.75	19.51	EDGHpGKPGRpGERG	COL1A2	Collagen alpha-2(I) chain	5.65E-07	8.99E-06	164.10	314.00	0.52	41.18	62.26
e19107	3801.79	33.48	DQGPVGRTGEVGAVGPpGFAGEKGpSGEAGTAGPPGTpGPQG	COL1A2	Collagen alpha-2(I) chain	1.60E-04	1.23E-03	115.99	221.47	0.52	47.06	61.81
e18573	3616.72	33.07	DQGPVGRTGEVGAVGpPGFAGEKGPSGEAGTAGPpGTpGP	COL1A2	Collagen alpha-2(I) chain	9.14E-03	3.49E-02	51.38	95.30	0.54	26.05	37.85
e05074	1447.70	19.50	DGHpGKpGRPGERG	COL1A2	Collagen alpha-2(I) chain	3.86E-04	2.58E-03	166.08	295.51	0.56	47.06	61.70
e19752	4098.90	24.55	GEVGPAGpNGFAGpAGAAGQp-GAKGERGAKGPKGENGVVGPTGpVG	COL1A2	Collagen alpha-2(I) chain	1.82E-08	5.42E-07	517.66	917.25	0.56	55.46	80.07
e05209	1463.69	19.50	DGHpGKpGRpGERG	COL1A2	Collagen alpha-2(I) chain	3.52E-03	1.65E-02	73.40	128.95	0.57	35.29	48.04
e11265	2194.97	20.12	NDGPpGRDGQpGHKGERGYpG	COL1A2	Collagen alpha-2(I) chain	7.78E-05	6.81E-04	58.69	102.96	0.57	30.25	47.26
e19745	4097.86	24.65	SKGESGNKGEpGSAGPQGPpGpSGEEGKRGPNGEAGSAG-PPGPpG	COL1A2	Collagen alpha-2(I) chain	1.36E-08	4.55E-07	791.47	1350.19	0.59	72.27	88.69
e06182	1579.68	23.04	GpAGPRGERGPpGESGA	COL1A2	Collagen alpha-2(I) chain	5.21E-07	8.41E-06	313.20	520.18	0.60	64.71	81.86
e16869	3169.50	25.46	ATGDRGEAGAAGPAGPAGpRGSPGERGEVGPAGPNG	COL1A2	Collagen alpha-2(I) chain	8.63E-07	1.26E-05	55.48	90.86	0.61	13.45	37.85
e17869	3410.58	31.13	PAGATGDRGEAGAAGPAGPAGpRGSpGERGEVGPAGPNG	COL1A2	Collagen alpha-2(I) chain	4.00E-05	3.84E-04	161.33	248.93	0.65	35.29	58.57
e18907	3735.73	27.23	TGDPGKNGDKGHAGLAGARGAPGpDGN-NGAQGppGPQGVQG	COL1A2	Collagen alpha-2(I) chain	5.23E-05	4.87E-04	119.90	175.24	0.68	40.34	60.25
e05559	1508.66	24.06	GpAGpRGERGPPGESG	COL1A2	Collagen alpha-2(I) chain	9.13E-03	3.49E-02	60.25	86.63	0.70	28.57	41.32
e00644	941.44	20.27	EpGEKGPRG	COL1A2	Collagen alpha-2(I) chain	4.47E-03	2.00E-02	183.91	258.84	0.71	55.46	68.65

Pep-tide ID	Mass [Da]	CE-time [Min]	Sequence	Symbol	Protein name	p-value	Adj. p-value (BH)	Average cases	Average controls	Fold change	Freq. cases	Freq. controls
e17212	3248.55	30.60	RTGEVGAVGPpGFAGEKGPSGEAGTAGpPGTpGPQG	COL1A2	Collagen alpha-2(I) chain	7.79E-07	1.14E-05	1386.48	1938.84	0.72	79.83	96.08
e10518	2080.93	20.25	DGPpGRDGQpGHKGERGYpG	COL1A2	Collagen alpha-2(I) chain	6.08E-07	9.54E-06	195.29	270.40	0.72	36.97	63.05
e15948	2960.36	29.70	LVGEpGPAGSKGESGNKGEpGSAGPQGPpGPSG	COL1A2	Collagen alpha-2(I) chain	3.34E-03	1.58E-02	13.36	18.22	0.73	5.88	17.02
e03691	1303.55	27.13	YDGKGVGLGPGPMG	COL1A2	Collagen alpha-2(I) chain	1.13E-03	6.30E-03	895.43	1194.72	0.75	68.07	85.78
e16547	3092.44	36.22	TGEVGAVGPpGFAGEKGPSGEAGTAGpPGTpGPQG	COL1A2	Collagen alpha-2(I) chain	6.89E-03	2.79E-02	146.32	188.13	0.78	46.22	62.15
e00421	902.42	20.86	DpGKNGDKG	COL1A2	Collagen alpha-2(I) chain	1.22E-03	6.67E-03	408.30	510.10	0.80	64.71	75.70
e17279	3264.53	30.51	RTGEVGAVGPpGFAGEKGpSGEAGTAGpPGTpGPQG	COL1A2	Collagen alpha-2(I) chain	5.07E-03	2.22E-02	406.55	484.76	0.84	76.47	88.80
e11186	2184.96	33.01	GPpGpAGSRGDGGPpGMTGFpGAAG	COL1A2	Collagen alpha-2(I) chain	2.28E-03	1.14E-02	116.20	120.20	0.97	34.45	52.30
e16390	3058.44	30.62	pNGPPGpAGSRGDGGpPGMTGFPGAAGRTGPpGP	COL1A2	Collagen alpha-2(I) chain	1.43E-02	4.94E-02	151.19	153.26	0.99	47.90	62.60
e07217	1691.81	30.35	GSpGNIGPAGKEGPVGLpG	COL1A2	Collagen alpha-2(I) chain	1.10E-02	4.06E-02	1425.90	1302.50	1.09	74.79	68.31
e14368	2674.20	20.11	EAGRDGNpGNDGpPGRDGQpGHKGER	COL1A2	Collagen alpha-2(I) chain	8.83E-04	5.07E-03	27.33	24.16	1.13	15.97	30.46
e15400	2853.33	23.85	LkGQpGApGVkGEpGApGENGTpGQTGARG	COL1A2	Collagen alpha-2(I) chain	1.22E-03	6.67E-03	1202.08	937.52	1.28	85.71	77.38
e08442	1822.74	30.83	AGEKGPSGEAGTAGPpGTpGP	COL1A2	Collagen alpha-2(I) chain	2.86E-03	1.37E-02	308.51	223.29	1.38	35.29	50.84
e06165	1577.78	20.65	RGLpGERGRVGApGPA	COL1A2	Collagen alpha-2(I) chain	7.85E-04	4.63E-03	52.96	34.83	1.52	19.33	9.41
e19692	4066.97	21.12	GERGAAGIpGGKGEKGEpGLRGEIGNpGRDGARGAP-GAVGApGP	COL1A2	Collagen alpha-2(I) chain	5.87E-03	2.47E-02	293.46	181.47	1.62	41.18	29.45
e02795	1209.53	26.33	GPpGPDGNKGEpG	COL1A2	Collagen alpha-2(I) chain	1.72E-03	8.95E-03	1014.71	590.18	1.72	84.03	79.06
e11629	2244.08	19.54	AGPpGKAGEDGHpGKpGRpGERG	COL1A2	Collagen alpha-2(I) chain	2.00E-04	1.46E-03	444.11	237.17	1.87	59.66	47.26
e13891	2593.33	19.91	ARGPAGPpGKAGEDGHPGKPGRpGERG	COL1A2	Collagen alpha-2(I) chain	8.29E-05	7.19E-04	61.79	31.25	1.98	12.61	4.14
e08482	1828.01	24.57	KEGPVGLpGIDGRPGPIGP	COL1A2	Collagen alpha-2(I) chain	1.04E-03	5.84E-03	58.94	28.22	2.09	11.76	4.59
e17105	3224.66	25.40	TGAKGAAGLpGVAGApGLpGPRGIpGPVGAAGATGARG	COL1A2	Collagen alpha-2(I) chain	1.14E-08	4.00E-07	699.32	310.27	2.25	61.34	37.07
e19324	3891.77	24.39	EPGSAGPQGPPGPSGEEGKRGPNGEAGSAGPPGppGLRGSpGS	COL1A2	Collagen alpha-2(I) chain	1.66E-04	1.26E-03	3573.68	1492.25	2.39	75.63	80.74
e13572	2542.19	23.08	HGEFGLpGPAGPRGERGpPGESGAAGP	COL1A2	Collagen alpha-2(I) chain	7.28E-10	4.37E-08	150.10	58.67	2.56	32.77	12.09
e20265	4416.15	22.51	GpAGKDGRTGHPGTVGPAGIRGPQGHQGPAG-PPGPPGPPGPPGVSGGGY	COL1A2	Collagen alpha-2(I) chain	1.40E-07	2.99E-06	22284.79	6957.90	3.20	63.03	45.69
e18344	3548.69	21.50	KGPTGDpGKNGDKGHAGLAGARGApGPDGNNGAQGPpGP	COL1A2	Collagen alpha-2(I) chain	3.90E-06	4.68E-05	299.74	89.43	3.35	15.97	5.15
e10513	2079.96	25.58	RGSpGSRGLpGADGRAGVMGPP	COL1A2	Collagen alpha-2(I) chain	6.11E-08	1.54E-06	2534.44	719.53	3.52	60.50	42.22
e10832	2129.98	32.86	GVQGGKGEQGPPGPPGFQGLpGP	COL1A2	Collagen alpha-2(I) chain	1.20E-06	1.71E-05	132.70	36.78	3.61	28.57	13.10
e09972	2005.92	25.12	GEEGKRGPNGEAGSAGPpGPpG	COL1A2	Collagen alpha-2(I) chain	1.28E-05	1.38E-04	189.51	52.03	3.64	34.45	19.37
e11611	2242.03	26.40	IRGPQGHQGPAGPpGPpGPpGPpG	COL1A2	Collagen alpha-2(I) chain	2.72E-14	1.04E-11	537.58	140.63	3.82	68.07	39.42
e10318	2054.95	25.44	GEpGPAGSKGESGNKGEpGSAGP	COL1A2	Collagen alpha-2(I) chain	1.54E-04	1.20E-03	381.34	92.79	4.11	26.89	49.27
e08859	1871.93	21.28	SGPpGPpGPAGKEGLRGPRG	COL1A2	Collagen alpha-2(I) chain	2.90E-11	2.66E-09	182.21	43.02	4.24	30.25	9.52
e05999	1560.81	23.09	KEGPVGLpGIDGRPGP	COL1A2	Collagen alpha-2(I) chain	7.08E-10	4.37E-08	405.43	88.76	4.57	33.61	13.10
e13555	2538.23	23.10	ISGPpGPpGPAGKEGLRGPRGDQGPVG	COL1A2	Collagen alpha-2(I) chain	6.94E-05	6.26E-04	217.39	47.22	4.60	36.13	21.72
e15742	2920.37	21.93	SKGESGNKGEpGSAGPQGPPGPSGEEGKRGp	COL1A2	Collagen alpha-2(I) chain	4.93E-09	1.88E-07	214.34	44.93	4.77	19.33	5.15
e17219	3249.55	30.71	RTGEVGAVGPpGFAGEKGpSGEAGTAGpPGTPGPqG	COL1A2	Collagen alpha-2(I) chain	8.09E-10	4.57E-08	502.06	105.04	4.78	43.70	19.71
e16873	3171.59	20.85	VGKpGERGLHGEFGLpGPAGPRGERGppGESG	COL1A2	Collagen alpha-2(I) chain	4.37E-04	2.85E-03	73.08	13.73	5.32	14.29	5.71
e04648	1405.66	28.35	GISGPpGPpGPAGKEG	COL1A2	Collagen alpha-2(I) chain	4.05E-07	6.82E-06	368.86	68.66	5.37	51.26	33.59
e11510	2228.05	19.61	AGPpGKAGEDGHpGKpGRPGERG	COL1A2	Collagen alpha-2(I) chain	8.64E-05	7.44E-04	395.70	66.45	5.95	36.97	22.84
e12693	2382.14	19.80	GPAGpPGKAGEDGHPGKpGRpGERG	COL1A2	Collagen alpha-2(I) chain	6.40E-03	2.64E-02	214.83	34.97	6.14	36.13	25.87

Pep-tide ID	Mass [Da]	CE-time [Min]	Sequence	Symbol	Protein name	p-value	Adj. p-value (BH)	Average cases	Average controls	Fold change	Freq. cases	Freq. controls
e01185	1020.52	25.27	NAGPAGPAGPRG	COL1A2	Collagen alpha-2(I) chain	4.51E-05	4.29E-04	58.31	9.33	6.25	13.45	4.48
e11382	2212.09	19.64	AGPpGKAGEDGHPGKPGRpGERG	COL1A2	Collagen alpha-2(I) chain	1.12E-09	5.55E-08	132.16	17.76	7.44	32.77	13.10
e08582	1841.85	24.22	VGEPGpAGSKGESGNKGEpG	COL1A2	Collagen alpha-2(I) chain	2.53E-03	1.24E-02	121.82	15.69	7.76	17.65	9.52
e09366	1928.90	24.97	VGEPGpAGSKGESGNKGEpGS	COL1A2	Collagen alpha-2(I) chain	6.71E-03	2.74E-02	259.92	32.73	7.94	17.65	10.41
e04292	1363.64	28.57	KGEPGSAGpQGPpGP	COL1A2	Collagen alpha-2(I) chain	9.99E-03	3.73E-02	526.93	64.11	8.22	19.33	12.09
e07405	1710.75	31.05	EKGpSGEAGTAGPpGTpGP	COL1A2	Collagen alpha-2(I) chain	4.83E-04	3.12E-03	188.88	20.02	9.43	24.37	13.21
e00157	845.40	23.40	SpGERGEV	COL1A2	Collagen alpha-2(I) chain	2.04E-03	1.04E-02	110.26	10.13	10.89	20.17	11.31
e01938	1115.56	26.55	YDGKGVGLGPGP	COL1A2	Collagen alpha-2(I) chain	8.18E-10	4.57E-08	220.79	16.31	13.54	16.81	3.70
e00536	924.45	24.12	GRVGApGPAGA	COL1A2	Collagen alpha-2(I) chain	5.42E-05	5.00E-04	245.48	11.18	21.95	17.65	7.05
e02963	1228.58	27.34	FpGAAGRTGppGP	COL1A2	Collagen alpha-2(I) chain	1.63E-07	3.33E-06	276.41	10.08	27.42	15.13	4.03
e02105	1134.59	23.99	PIGQEGAPGRPG	COL4A2	Collagen alpha-2(IV) chain	3.35E-05	3.31E-04	36.53	76.10	0.48	10.92	29.68
e16610	3109.40	29.91	DTGNPGAPGTpGTKGWAGDSGpQGRpGVFGLPG	COL4A2	Collagen alpha-2(IV) chain	5.49E-04	3.45E-03	33.29	66.51	0.50	15.13	31.13
e11768	2264.94	43.13	pGFPGAQGEPGSQGEpGDpGLpGP	COL4A2	Collagen alpha-2(IV) chain	8.54E-04	4.95E-03	103.97	33.56	3.10	28.57	17.25
e15535	2877.32	24.25	GETGPHGYKGMVGAIGATGPpGEEGpRGpP	COL9A2	Collagen alpha-2(IX) chain	2.57E-07	4.74E-06	148.90	186.25	0.80	21.01	49.38
e12015	2297.99	33.82	PpGppGPpGVPGSDGIDGDNGPPGK	COL9A2	Collagen alpha-2(IX) chain	1.32E-02	4.64E-02	290.63	327.35	0.89	57.98	71.22
e06314	1595.69	22.81	PGpKGEpGKAGpDGpDG	COL9A2	Collagen alpha-2(IX) chain	8.71E-04	5.03E-03	126.90	142.09	0.89	30.25	46.58
e14936	2776.34	21.50	VGMMGPPGpPGPPGYpGKQGpHGHpGPR	COL9A2	Collagen alpha-2(IX) chain	2.15E-13	4.08E-11	721.23	171.16	4.21	63.03	35.16
e06668	1632.80	30.84	ERGpPGpPGpPGVpGSD	COL9A2	Collagen alpha-2(IX) chain	1.24E-04	1.01E-03	564.58	94.48	5.98	31.93	19.04
e08304	1807.80	23.88	pGQpGTKGGpGDQGEPGPQ	COL9A2	Collagen alpha-2(IX) chain	1.11E-02	4.08E-02	81.49	13.11	6.22	5.04	13.77
e15532	2876.35	34.48	GPTGATGDKGPPGPVGpPGSNGPVGEPGPEGPA	COL5A2	Collagen alpha-2(V) chain	4.87E-03	2.15E-02	7.84	36.08	0.22	5.04	14.45
e20387	4523.06	26.21	PGPVGpPGSNGPVGEPGPEGPAGNDGTPGRDGAVGERG-DRGDPGPAGLPG	COL5A2	Collagen alpha-2(V) chain	3.80E-05	3.69E-04	11.91	36.71	0.32	6.72	23.29
e18809	3697.72	27.26	TGPMGAMGPLGPRGMPGERGRLGpQGApGQRGAHGMpG	COL5A2	Collagen alpha-2(V) chain	2.07E-03	1.05E-02	13.36	38.29	0.35	3.36	13.44
e06214	1583.65	39.73	GSPGTSGppGSAGpPGSpG	COL5A2	Collagen alpha-2(V) chain	2.39E-07	4.52E-06	198.82	558.01	0.36	34.45	60.69
e16344	3049.35	29.64	VGEpGpEGpAGNDGTPGRDGAVGERGDRGDPG	COL5A2	Collagen alpha-2(V) chain	2.59E-07	4.74E-06	127.51	308.95	0.41	27.73	53.08
e15594	2889.37	28.80	PGSRGENGpTGAVGFAGPQGPDGQPGVKGEP	COL5A2	Collagen alpha-2(V) chain	1.55E-07	3.21E-06	635.24	339.61	1.87	84.03	75.03
e16571	3098.48	29.41	LTGNpGVQGPEGKLGPLGApGEDGRpGpPGSIG	COL5A2	Collagen alpha-2(V) chain	1.34E-02	4.68E-02	96.96	51.53	1.88	15.97	8.96
e15320	2838.39	24.16	TQGPPGATGFPGSAGRVGPpGpAGAPGPAGPLG	COL5A2	Collagen alpha-2(V) chain	1.26E-05	1.37E-04	2050.94	985.83	2.08	93.28	91.83
e03494	1283.55	27.39	DKGDpGEDGQPGP	COL5A2	Collagen alpha-2(V) chain	3.81E-03	1.77E-02	61.11	26.11	2.34	3.36	12.43
e04424	1379.63	28.20	DKGpPGpVGPpGSNG	COL5A2	Collagen alpha-2(V) chain	1.69E-07	3.40E-06	357.03	52.26	6.83	21.85	7.73
e00028	809.32	34.76	GEPGPEGPA	COL5A2	Collagen alpha-2(V) chain	1.26E-03	6.86E-03	11.00	1.32	8.31	10.92	4.14
e03206	1253.55	21.34	PGTSGPpGSAGpPGS	COL5A2	Collagen alpha-2(V) chain	1.31E-02	4.63E-02	996.08	65.96	15.10	13.45	24.75
e16764	3148.46	31.26	KGEpGLPGpPGEGRAGEPGTAGPTGPPGVPGSpGI	COL8A2	Collagen alpha-2(VIII) chain	1.66E-05	1.76E-04	33.69	135.34	0.25	18.49	36.84
e18758	3677.82	24.29	pGAPGQGGAPGPPGLPGPAGLGKPGLDGLP-GAPGDKGESGPPG	COL8A2	Collagen alpha-2(VIII) chain	1.39E-03	7.51E-03	354.44	517.92	0.68	47.90	67.86
e02699	1198.54	26.12	GPpGFQGEpGPQ	COL8A2	Collagen alpha-2(VIII) chain	8.36E-03	3.25E-02	180.36	105.43	1.71	52.94	43.00
e13748	2571.16	34.64	EGRAGEPGTAGPTGppGVPGSPGITGPpG	COL8A2	Collagen alpha-2(VIII) chain	1.14E-02	4.16E-02	76.73	29.38	2.61	32.77	24.97
e15401	2853.34	28.33	GTAGPTGPPGVpGSpGITGpPGPPGPPGPPGAPG	COL8A2	Collagen alpha-2(VIII) chain	3.33E-04	2.28E-03	534.37	97.58	5.48	32.77	21.05
e09792	1980.91	25.56	GPTGPKGEpGFTGRpGGPGVAG	COL8A2	Collagen alpha-2(VIII) chain	1.24E-04	1.01E-03	4397.02	748.19	5.88	10.08	3.02
e03383	1271.62	21.83	KGEpGFTGRPGGP	COL8A2	Collagen alpha-2(VIII) chain	1.28E-12	1.84E-10	330.17	17.58	18.78	19.33	3.70

Pep-tide ID	Mass [Da]	CE-time [Min]	Sequence	Symbol	Protein name	p-value	Adj. p-value (BH)	Average cases	Average controls	Fold change	Freq. cases	Freq. controls
e17816	3397.56	31.76	NGADGPQGPpGGVGNLGppGEKGEPGESGSPGIQGEP	COL11A2	Collagen alpha-2(XI) chain	7.20E-07	1.10E-05	9.68	119.81	0.08	4.20	23.96
e14918	2774.37	21.54	pGPEGPAGLIGPPGIQGNPGPVGDPGERGP	COL11A2	Collagen alpha-2(XI) chain	1.04E-03	5.85E-03	138.02	131.40	1.05	17.65	8.40
e07199	1689.74	40.69	pGPpGQQGTPGTQGLpGP	COL11A2	Collagen alpha-2(XI) chain	4.82E-03	2.13E-02	2186.35	1623.47	1.35	72.27	56.44
e20509	4654.14	25.78	GEHGpPGPPGPIGPVGQPGAAGADGEPGARGPQGHF-GAKGDEGTRGFNGP	COL11A2	Collagen alpha-2(XI) chain	7.29E-05	6.52E-04	12302.68	8672.79	1.42	96.64	95.07
e09631	1962.89	41.85	EPGppGQQGTpGTQGLpGPQ	COL11A2	Collagen alpha-2(XI) chain	1.94E-03	9.97E-03	79.21	28.49	2.78	12.61	5.26
e18396	3560.70	26.10	GLPGPKGELALSGEKGDQGPPGDpGSpGSpGPAGPAGPPG	COL4A3	Collagen alpha-3(IV) chain	1.98E-04	1.45E-03	11.65	43.60	0.27	6.72	21.28
e09534	1948.99	24.80	EGPRGAQGLpGLNGLKGQQG	COL4A3	Collagen alpha-3(IV) chain	5.30E-04	3.38E-03	4269.30	6042.64	0.71	68.91	84.66
e15360	2847.26	24.37	GpKGDpGIpGLDRSGFpGETGSPGIPGHQ	COL4A3	Collagen alpha-3(IV) chain	7.81E-04	4.62E-03	332.44	456.93	0.73	57.98	78.05
e07481	1717.90	23.77	KGPPGDHGLPGYLGSPGI	COL4A3	Collagen alpha-3(IV) chain	3.97E-03	1.82E-02	1183.98	1074.49	1.10	64.71	52.41
e09656	1964.99	24.86	KpGTPGPAGEKGNKGSKGEPG	COL4A3	Collagen alpha-3(IV) chain	1.45E-02	4.98E-02	986.81	405.93	2.43	52.10	46.02
e10747	2115.92	32.77	PGTpGNEGLDGpRGDPGQPGpP	COL4A3	Collagen alpha-3(IV) chain	9.23E-03	3.51E-02	59.12	21.19	2.79	22.69	14.67
e18186	3502.75	19.29	PGPAGEKGNKGSKGEPGpAGSDGLpGLKGKRGDSGSPA	COL4A3	Collagen alpha-3(IV) chain	1.40E-03	7.53E-03	991.13	337.71	2.93	37.82	27.32
e01534	1068.47	26.76	PGDpGSpGSpGP	COL4A3	Collagen alpha-3(IV) chain	2.70E-03	1.32E-02	101.03	19.41	5.21	10.08	4.03
e18343	3548.62	26.82	LPGDMGKKGEmGQPGPpGHLGpAGPEGAPGSPGSpGLP	COL4A3	Collagen alpha-3(IV) chain	2.17E-03	1.09E-02	49.14	4.97	9.90	11.76	5.04
e17817	3398.57	31.83	ELGEAGPSGEpGVPGDAGMPGERGEAGHRGSAGALGP	COL9A3	Collagen alpha-3(IX) chain	1.37E-06	1.88E-05	16.41	99.22	0.17	10.92	31.80
e18022	3454.57	31.88	PGpRGNQGDRGDKGAAGAGLDGpEGDQGPQGPQGVPG	COL9A3	Collagen alpha-3(IX) chain	1.14E-03	6.34E-03	27.34	94.64	0.29	10.08	23.07
e19394	3926.83	33.54	GDKGAAGAGLD-GPEGDQGpQGpQGVpGTSKDGQDGAPGEPGPP	COL9A3	Collagen alpha-3(IX) chain	2.57E-04	1.81E-03	12.57	36.28	0.35	13.45	28.89
e11916	2283.98	27.27	QGDRGDKGAAGAGLDGpEGDQGpQ	COL9A3	Collagen alpha-3(IX) chain	1.94E-04	1.43E-03	119.80	185.95	0.64	31.93	50.73
e12993	2431.28	26.91	LpGPPGPKGAPGKPGKPGEAGLPGLPG	COL9A3	Collagen alpha-3(IX) chain	9.07E-04	5.19E-03	66.82	40.56	1.65	13.45	5.49
e13568	2541.23	22.96	GpTGYKGEQGEVGKDGEKGDPGPpGP	COL9A3	Collagen alpha-3(IX) chain	2.79E-07	5.02E-06	1629.80	617.22	2.64	46.22	26.76
e09669	1965.99	24.88	LGRPGPKGTPGVAGpSGEpGm	COL9A3	Collagen alpha-3(IX) chain	5.18E-04	3.32E-03	510.13	161.94	3.15	36.97	25.64
e15047	2795.42	21.53	RDGpPGpKGApGERGSLGPPGpPGLGGKGL	COL9A3	Collagen alpha-3(IX) chain	2.96E-09	1.21E-07	913.61	70.47	12.96	17.65	4.37
e10547	2085.03	22.00	GPpGPPGSIGHpGARGPpGYRG	COL9A3	Collagen alpha-3(IX) chain	1.14E-06	1.63E-05	86.27	3.12	27.68	13.45	3.58
e17754	3376.63	31.72	IDGSpGEKGDPGDVGGPGPPGASGEPGAPGPPGKRGPS	COL5A3	Collagen alpha-3(V) chain	1.07E-08	3.83E-07	65.22	310.53	0.21	26.89	50.50
e10797	2123.88	21.88	DGSpGEKGDpGDVGGPGPPGASGE	COL5A3	Collagen alpha-3(V) chain	1.94E-07	3.80E-06	1453.08	2001.57	0.73	68.91	90.48
e01666	1084.52	21.20	GSKGDKGDAGPP	COL5A3	Collagen alpha-3(V) chain	3.19E-04	2.19E-03	104.27	52.77	1.98	19.33	36.28
e18537	3603.77	19.35	GAQGpPGSAGPPGYPGPRGVKGTSGNRGLQGEKGEKGE	COL5A3	Collagen alpha-3(V) chain	2.34E-03	1.16E-02	518.52	148.23	3.50	32.77	21.95
e11834	2273.97	26.85	SPGEKGDPGDVGGPGPpGASGEpGAP	COL5A3	Collagen alpha-3(V) chain	9.20E-03	3.51E-02	174.69	27.73	6.30	21.85	13.89
e05952	1555.68	29.36	VGPPGEpGPpGQQGNH	COL5A3	Collagen alpha-3(V) chain	6.77E-04	4.10E-03	541.40	76.88	7.04	47.06	38.75
e19151	3818.73	37.77	GDLGpPGDpGVSGIDGSPGEKGDPGDVGGPGPP-GASGEPGAPGP	COL5A3	Collagen alpha-3(V) chain	3.48E-03	1.63E-02	414.58	31.96	12.97	21.01	12.09
e06294	1593.71	22.82	PFGDDGLpGppGPkGP	COL4A4	Collagen alpha-4(IV) chain	1.87E-04	1.39E-03	55.66	128.31	0.43	15.97	33.03
e05380	1486.69	21.29	PGDQGPPGpDGPRGAP	COL4A4	Collagen alpha-4(IV) chain	5.93E-05	5.44E-04	87.61	125.54	0.70	37.82	57.00
e08406	1818.83	24.27	HEDATpGGKGFPGPLGpPG	COL4A4	Collagen alpha-4(IV) chain	6.99E-03	2.83E-02	397.64	127.76	3.11	60.50	49.38
e08096	1782.76	40.37	GFpGLPGDQGEPGSpGppG	COL4A4	Collagen alpha-4(IV) chain	1.12E-02	4.09E-02	47.95	11.39	4.21	19.33	11.76
e08942	1878.85	32.22	pGFPGTpGLpGmPGHDGAPG	COL4A5	Collagen alpha-5(IV) chain	4.17E-04	2.74E-03	99.97	103.55	0.97	28.57	46.81
e10156	2032.95	25.01	DGIPGPAGQKGEPGQPGFGNPG	COL4A5	Collagen alpha-5(IV) chain	1.19E-02	4.30E-02	24.51	16.56	1.48	14.29	7.50
e06269	1590.67	40.03	GLDGppGpDGLQGpPGP	COL4A5	Collagen alpha-5(IV) chain	3.04E-03	1.45E-02	371.05	191.42	1.94	57.98	47.70

Pep-tide ID	Mass [Da]	CE-time [Min]	Sequence	Symbol	Protein name	p-value	Adj. p-value (BH)	Average cases	Average controls	Fold change	Freq. cases	Freq. controls
e00320	880.39	23.62	RGLDGPpGP	COL4A5	Collagen alpha-5(IV) chain	4.20E-03	1.91E-02	64.33	18.34	3.51	25.21	15.34
e07367	1706.77	40.58	GDVGPNGQpGPmGPpGLp	COL4A5	Collagen alpha-5(IV) chain	3.94E-06	4.68E-05	151.99	42.25	3.60	35.29	18.14
e18802	3695.77	27.01	GIPGLPGDPGYPGEPGRDGEKGQKGDTGppGppGLVIP	COL4A5	Collagen alpha-5(IV) chain	3.72E-12	4.74E-10	242.78	61.69	3.94	47.06	20.04
e12852	2407.21	22.70	GpSITGVpGpAGLPGPKGEKGYPGIG	COL4A6	Collagen alpha-6(IV) chain	2.48E-04	1.76E-03	165.39	94.35	1.75	29.41	16.57
e09844	1987.97	25.15	GLDGERGRPGPAGppGppGPS	COL4A6	Collagen alpha-6(IV) chain	6.87E-03	2.79E-02	1122.72	575.45	1.95	73.11	67.53

p Hydroxylation of proline; *m* oxidation of methionine.

Supplementary Table 5: List of proteases responsible for generating the collagen peptides associated with mortality. The analysis was performed using the Proteasix tool [1] (observed mode), including only proteases for which cleavage-site associations have been reported in the literature. Only human proteases were considered, and cleavage observed in substrates other than the parental proteins were excluded. The number of cleavage sites and the regulation of peptides (increased or decreased in mortality) are reported.

Symbol	Uniprot Accession	607 collagen peptides			210 collagen peptides		
		**# CS**	**# ↓**	**# ↑**	**# CS**	**# ↓**	**# ↑**
MMP13	P45452	59	38	21	18	14	4
MMP9	P14780	46	27	19	16	14	2
MMP25	Q9NPA2	44	23	21	20	15	5
MMP8	P22894	19	16	3	9	9	0
MMP2	P08253	19	14	5	9	7	2
MMP12	P39900	17	14	3	7	7	0
MMP1	P03956	14	14	0	7	7	0
MMP14	P50281	13	8	5	5	3	2
MMP20	O60882	12	7	5	2	1	1
MMP16	P51512	11	11	0	5	5	0
MMP3	P08254	10	6	4	4	4	0
ADAMTS5	Q9UNA0	3	1	2			
MMP7	P09237	2	0	2			
ADAMTS2	O95450	2	1	1	1	1	0
CTSB	P07858	2	1	1	1	1	0
BMP1	P13497	1	0	1			
CTSL	P07711	1	0	1			
CTSK	P43235	1	0	1			

Abbreviations: CS = cleavage site

Supplementary Table 6: Prediction of short-term mortality in patients with critical condition stratified based on the COL210 score range.

**Characteristic**	**HR**[1]	**95% CI**[1]	**p-value**
COL210			
(-6,-2]	—	—	
(-2,-0.5]	1.10	0.73, 1.67	0.6
(-0.5,1]	1.58	1.07, 2.34	0.021
(1,6]	1.76	1.19, 2.60	0.004
Gender			
Male	—	—	
Female	0.84	0.71, 1.00	0.056
Kidney disease			
no	—	—	
yes	1.63	1.36, 1.95	<0.001
Diabetes			
no	—	—	
yes	0.96	0.78, 1.19	0.7

**Characteristic**	**HR[1]**	**95% CI[1]**	**p-value**
CVD			
no	—	—	
yes	1.25	0.93, 1.68	0.14
ht			
no	—	—	
yes	0.97	0.81, 1.17	0.8
n = 1,713; N events = 574; statistic.log = 70.4; p.value.log = 0.000; statistic.sc = 69.0; p.value.sc = 0.000; statistic.wald = 67.3; p.value.wald = 0.000; statistic.robust = NA; p.value.robust = NA; R^2 = 0.040; r.squared.max = 0.966; c-index = 0.625; c-index SE = 0.013; Log-likelihood = -2,854; AIC = 5,723; BIC = 5,758; No. Obs. = 1,713			

Supplementary Table 7: Risk groups based on COL210 score quintiles in the ICU cohort. Days to 50% risk of death were estimated based on the established regression analysis.

Score group (range)	Days to 50% risk of death in ICU (median, 95% CI)
5 (1.767 to 5.935)	231.48 (122.01- 278.28)
4 (0.768 to 1.758)	322.74 (282.60 - 370.70)
3 (-0.175 to 0.766)	421.68 (374.64 - 485.02)
2 (-1.299 to -0.180)	559.09 (491.04 - 665.29)
1 (-4.480 to -1.301)	816.72 (679.84 - 1375.10)

Supplementary Table 8: Prediction of mortality in patients with non-critical condition.

**Characteristic**	**HR**[1]	**95% CI**[1]	**p-value**
COL210			
(-6,-2]	—	—	
(-2,-0.5]	1.69	1.40, 2.03	<0.001
(-0.5,1]	1.91	1.48, 2.45	<0.001
(1,6]	2.20	1.56, 3.10	<0.001
Gender			
Male	—	—	
Female	0.74	0.63, 0.88	<0.001
Kidney disease			
no	—	—	
Yes	1.05	0.80, 1.38	0.7
Diabetes			
no	—	—	
yes	1.39	1.13, 1.70	0.001
CVD			

Characteristic	HR[1]	95% CI[1]	p-value
no	—	—	
yes	3.15	2.62, 3.78	<0.001
ht			
no	—	—	
yes	1.22	0.99, 1.51	0.062
map	0.99	0.98, 0.99	<0.001
egfr	0.99	0.99, 1.00	0.020
n = 7,474; N events = 625; statistic.log = 295; p.value.log = 0.000; statistic.sc = 302; p.value.sc = 0.000; statistic.wald = 284; p.value.wald = 0.000; statistic.robust = NA; p.value.robust = NA; R^2 = 0.039; r.squared.max = 0.605; c-index = 0.687; c-index SE = 0.016; Log-likelihood = -3,319; AIC = 6,658; BIC = 6,703; No. Obs. = 7,474			

Supplementary Table 9: Risk groups based on COL210 score quintiles in the non-ICU cohort. Days to 50% risk of death were estimated based on the establis-hed regression analysis.

Score group (range)	Days to 50% risk of death in non-ICU (median, 95% CI)
5 (-0.754 to 5.270)	5039.78 (1644.89 to 7114.14)
4 (-1.706 to -0.755)	8970.25 (7308.42 to 10501.92)
3 (-2.369 to -1.707)	12137.51 (10650.63 to 13698.77)
2 (-3.105 to -2.370)	15946.76 (13903.96 to 18467.53)
1 (-5.999 to -3.106)	≥ 18602.04 **

** No detectable risk of death in a reasonable time frame.

Supplementary Table 10: Age-stratified Cox regression analyses among ICU and non-ICU patients. For each age stratum, unadjusted models were first fitted to estimate the crude association between the COL210 score and mortality. Adjusted models were then fitted, including sex, presence of kidney disease, cardiovascular disease, hypertension, mean arterial pressure, and estimated glomerular filtration rate as covariates (*). Hazard ratios (HRs) and 95% confidence intervals (CIs) are reported.

Age group	n	events	HR	LCI	UCI	p-value	HR_adj*	LCI_adj*	UCI_adj*	p-value _adj*
**ICU:**										
(15-45]	322	40	1.24	1.05	1.46	1.00E-02	1.23	1.02	1.48	2.75E-02
(45-60]	460	103	1.31	1.16	1.47	1.07E-05	1.23	1.08	1.40	2.17E-03
(60-75]	575	242	1.19	1.10	1.29	8.36E-06	1.15	1.06	1.25	1.15E-03
(75-100]	356	189	1.05	0.97	1.15	2.35E-01	1.04	0.95	1.14	4.19E-01
**Non-ICU:**										
(15-45]	1580	11	1.07	0.67	1.72	7.65E-01	1.12	0.66	1.90	6.79E-01
(45-60]	2337	78	1.34	1.18	1.53	1.14E-05	1.24	1.06	1.46	8.04E-03
(60-75]	2965	289	1.30	1.21	1.40	1.13E-12	1.21	1.11	1.32	6.49E-06
(75-100]	592	247	1.27	1.17	1.38	1.01E-08	1.20	1.10	1.31	8.25E-05

Abbreviations: adj.= adjusted Cox model, HR= hazard ratio, ICU= intensive care unit, LCI= lower confidence interval, UCI= upper confidence interval

Supplementary Table 11: Association between COL210 and mortality in ICU and non-ICU patients. To formally assess whether the association between COL210 and mortality differed by age group, interaction analyses were conducted using Cox models that included a multiplicative interaction term between COL210 and age group. For both unadjusted and adjusted analyses, models with and without the interaction term were compared using likelihood ratio tests. Adjusted models included sex, presence of kidney disease, cardiovascular disease, hypertension, mean arterial pressure, and estimated glomerular filtration rate as covariates (*).

**Analysis**	**Model comparison**	**$\Delta\chi^2$**	**df**	**p-value**
**ICU:**				
Unadjusted	COL210 + age group vs. COL210 × age group	9.28	3	0.026
Adjusted*	COL210 + age group + covariates vs. COL210 × age group + covariates	11.09	3	0.011
**Non-ICU:**				
Unadjusted	COL210 + age group vs. COL210 × age group	1.62	3	0.66
Adjusted*	COL210 + age group + covariates vs. COL210 × age group + covariates	1.18	3	0.76

Abbreviations: df= degree of freedome, ICU= intensive care unit, **$\Delta\chi^2$ =** Change in chi-square statistic

Supplementary Table 12: Underlying comorbidity stratified Cox regression analyses among non-ICU patients. Hazard ratios (HRs) and 95% confidence intervals (CIs) are reported.

Disease	n	events	HR	LCI	UCI	p-value
Diabetes	4101	163	1.6288	1.4814	1.7909	<0.0001
Kidney disease	2212	314	1.1878	1.1128	1.2679	<0.0001
Hypertension	3090	332	1.3457	1.2634	1.4335	<0.0001
Cardiovascular disease	1357	374	1.2185	1.1402	1.3021	<0.0001

Abbreviations: HR= hazard ratio, ICU= intensive care unit, LCI= lower confidence interval, UCI= upper confidence interval

Supplementary Table 13: Correlation of the COL210 score with IFTA stratified by CKD aetiology. Spearman correlation analyses were conducted for the most representative CKD aetiologies (n ≥ 50).

CKD etiology	sample size	rho	p-value	95%CI
ATN	58	0.162	0.2231	-0.100 to 0.0404
DKD	81	0.243	0.0287	0.0263 to 0.438
Vasculitis	84	0.259	0.0172	0.0477 to 0.449
IgAN	186	0.243	0.0008	0,103 to 0.374
FSGS	85	0.275	0.0108	0.0659 to 0.461
HTN	219	0.205	0.0023	0.0748 to 0.329
INTN	47	0.519	0.0001	0.272 to 0.701
MGN	50	0.378	0.0068	0.111 to 0.594

Abbreviations: CI = confidence interval, Abbreviations: ATN = acute tubular necrosis, DKD = diabetic kidney disease, FSGS = focal segmental glomerulosclerosis, HTN = hypersentsive nephropathy, INTN = Interstitial nephritis, IgAN = IgA nephropathy, MGN = membranous glomerulonephritis

Supplementary Table 14: Fisher's r-to-z comparison of independent correlations between selected CKD aetiologies.

Aetiology 1	Aetiology 2	z-value	p-value
ATN	DKD	0.480	0.631
ATN	Vasculitis	0.581	0.561
ATN	IgAN	0.550	0.583
ATN	FSGS	0.682	0.495
ATN	HTN	0.295	0.768
ATN	MGN	1.179	0.238
DKD	Vasculitis	0.108	0.914
DKD	IgAN	0.000	1.000
DKD	FSGS	0.217	0.828
DKD	HTN	-0.303	0.762
DKD	MGN	0.811	0.417
Vasculitis	IgAN	-0.128	0.898
Vasculitis	FSGS	0.110	0.912
Vasculitis	HTN	-0.438	0.661
Vasculitis	MGN	0.724	0.469
IgAN	FSGS	0.258	0.796
IgAN	HTN	-0.398	0.690
IgAN	MGN	0.916	0.360
FSGS	HTN	-0.573	0.567
FSGS	MGN	0.631	0.528
HTN	MGN	1.179	0.238

Abbreviations: ATN = acute tubular necrosis, DKD = diabetic kidney disease, FSGS = focal segmental glomerulosclerosis, HTN = hypersentsive nephropathy, IgAN = IgA nephropathy, MGN = membranous glomerulonephritis

References:

[1] Klein, J., Eales, J., Zurbig, P., Vlahou, A., *et al.*, Proteasix: a tool for automated and large-scale prediction of proteases involved in naturally occurring peptide generation. *Proteomics* 2013, *13*, 1077-1082.
